# A Hybrid Docking and Machine Learning Approach to Enhance the Performance of Virtual Screening Carried out on Protein–Protein Interfaces

**DOI:** 10.3390/ijms232214364

**Published:** 2022-11-18

**Authors:** Natesh Singh, Bruno O. Villoutreix

**Affiliations:** NeuroDiderot Department, Université de Paris, Inserm UMR 1141, Robert-Debré Hospital, 75019 Paris, France

**Keywords:** virtual screening, docking, scoring, protein-protein interaction inhibitors, machine learning

## Abstract

The modulation of protein–protein interactions (PPIs) by small chemical compounds is challenging. PPIs play a critical role in most cellular processes and are involved in numerous disease pathways. As such, novel strategies that assist the design of PPI inhibitors are of major importance. We previously reported that the knowledge-based DLIGAND2 scoring tool was the best-rescoring function for improving receptor-based virtual screening (VS) performed with the Surflex docking engine applied to several PPI targets with experimentally known active and inactive compounds. Here, we extend our investigation by assessing the vs. potential of other types of scoring functions with an emphasis on docking-pose derived solvent accessible surface area (SASA) descriptors, with or without the use of machine learning (ML) classifiers. First, we explored rescoring strategies of Surflex-generated docking poses with five GOLD scoring functions (GoldScore, ChemScore, ASP, ChemPLP, ChemScore with Receptor Depth Scaling) and with consensus scoring. The top-ranked poses were post-processed to derive a set of protein and ligand SASA descriptors in the bound and unbound states, which were combined to derive descriptors of the docked protein-ligand complexes. Further, eight ML models (tree, bagged forest, random forest, Bayesian, support vector machine, logistic regression, neural network, and neural network with bagging) were trained using the derivatized SASA descriptors and validated on test sets. The results show that many SASA descriptors are better than Surflex and GOLD scoring functions in terms of overall performance and early recovery success on the used dataset. The ML models were superior to all scoring functions and rescoring approaches for most targets yielding up to a seven-fold increase in enrichment factors at 1% of the screened collections. In particular, the neural networks and random forest-based ML emerged as the best techniques for this PPI dataset, making them robust and attractive vs. tools for hit-finding efforts. The presented results suggest that exploring further docking-pose derived SASA descriptors could be valuable for structure-based virtual screening projects, and in the present case, to assist the rational design of small-molecule PPI inhibitors.

## 1. Introduction

Virtual screening (VS) is one popular set of techniques to extract a list of potentially bioactive molecules from input chemical libraries [1,2,3,4,5,6,7]. Structure-based methods are of high interest to investigate novel mechanisms, such as protein–protein interactions, even more so in the era of AI-powered protein structures predictions [8,9,10,11] and major advances in the field of structural biology, such as Cryo-EM [12,13,14]. Structure-based virtual screening (SBVS) has been shown efficient in suggesting hit compounds on numerous targets, but the approach is not without pitfalls [15,16,17,18,19]. An important problem in virtual screening is that the methods are usually target-dependent, and as such, one method cannot fit all [15,17,18,19,20,21]. In SBVS studies, the compounds are first positioned in a binding pocket and then scored. Several docking programs have been shown proficient in reasonably sampling the binding site space and in generating reliable poses [20], but a key problem is scoring, resulting in a high number of inactive compounds ranked high in the scoring list or of active molecules lost. There are many sources of errors in scoring [15,16,17,18,19], including the difficulties of implementing complex energy terms in fast scoring functions and the obviously related challenges of taking into consideration water molecules and protein flexibility, among others. Several strategies have been proposed to try to improve the process, such as the use of more sophisticated CPU/GPU intensive energy computations, but the advantages are not obvious and are also target-dependent [15]. Molecular interaction fingerprints, machine learning (ML) scoring functions, target-specific scoring functions, and consensus scoring represent other approaches that have been shown valuable in some circumstances [22,23,24,25,26,27,28,29,30,31,32,33,34].

Protein–protein interactions (PPIs) regulate diverse types of biological activities both in healthy and disease states [35]. It is estimated that the human interactome comprises about 130,000–650,000 types of PPI [36,37]. The crucial importance of PPIs makes them a rich source of putative targets for the development of a new generation of therapeutics or for the design of chemical probes that could be used to validate further the importance of some specific PPIs. However, the nature of the PPI interfaces is in general significantly different from that of classical drug targets, which have well-defined pockets allowing the tight binding of small molecules [38]. The PPI binding interface is usually large (~1500–3000 Å^2^) and flat (often exposed to solvent) [39], posing substantial problems in ligand discovery using structure-based approaches. Our last study revealed DLIGAND2 as a best-rescoring function that significantly improved the vs. performance of Surflex docking for various PPI targets. DLIGAND2 is a knowledge-based scoring function that predicts protein–ligand binding affinity based on a distance-scaled, finite, ideal-gas reference (DFIRE) state [40]. Here, we extend our previous research by assessing the vs. potential of rescoring using GOLD scoring functions (GoldScore, ChemScore, Astex Statistical Potential (ASP), ChemPLP, ChemScore with Receptor Depth Scaling (RDS)). In addition, the poses were also scored using a consensus method involving all scoring functions. The best-predicted poses sorted on the Surflex docking score were further post-processed to derive 3D SASA descriptors of both the targets and the ligands in their bound and unbound states. The protein and ligand SASA descriptors were combined to derive a set of protein–ligand SASA descriptors. These descriptors were independently assessed to measure any possible impact on vs. metrics. Finally, these descriptors were employed for building eight diverse ML models: tree, bagged forest, random forest, Bayesian, support vector machine (SVM), logistic regression, neural network, and neural network with bagging. Several studies have been performed by exploiting chemical fingerprints to build ML-based quantitative structure–activity relationship (QSAR) models to separate true inhibitors of PPIs from inactive molecules [41,42,43,44]. These studies essentially aimed at the investigation of the physicochemical property ranges of compounds acting as inhibitors of PPIs. The identified properties were then used to build statistical models enabling the generation of compound collections likely enriched in inhibitors of PPIs [45,46,47]. However, the potential and effectiveness of descriptors calculated from the experimental 3D structure of a protein–ligand complex or of docking pose-generated 3D descriptors have not been fully investigated so far either for rescoring and ranking PPI compounds or for building classification models. The primary assumption in using these topological 3D descriptors to rank the poses is that the docking programs are capable of correctly sampling the binding site pockets and generating reliable poses, but they are not always accurate at scoring them for a wide range of protein targets. Here, we are interested in the evaluation of several post-processing strategies. First, we investigated the impact of using different types of scoring functions and a consensus scoring method on several PPI targets. Then, we investigated the potential benefit of using SASA data to rescore the molecules. In this case, we used the best docking poses generated by a fast method available in Surflex (assumed to be reasonable) and grades them using the derivatized SASA score. Then, we explored further the SASA descriptors, through various ML approaches, with the aims of generating some predictive classification models and identifying key SASA descriptors capable of discriminating between true active and true inactive ligands. The vs. performance on different targets was assessed by computing receiver operating characteristics (ROC) graphs and calculating the area under the curve (AUC) and the early recognition metrics (BEDROC, enrichment factors at 1% and 5%) to determine the ability of different rescoring methods to rank the active compounds in the top positions. The performance of ML models was estimated by calculating AUC, sensitivity, specificity, precision, and concordance. The results indicate that among the different GOLD scoring functions, GoldScore and ASP were found valuable for several targets while many SASA descriptors performed equivalently to or better than the default Surflex and GOLD scoring functions for most targets. Among the ML models, neural network and random forest classification models yielded superior vs. results for most targets. The present study provides valuable insights regarding rescoring strategies, and ML methods on PPIs. The presented protocols could assist the identification of novel drug-like orthosteric PPI inhibitors. This can be of significant interest with applications on novel targets acting in different diseases or to replace some monoclonal antibodies or peptides inhibiting already known targets.

## 2. Results

The workflow describing the hybrid computational approach developed in this study by employing docking-based VS, rescoring, and ML is shown in Figure S1. The ten PPI targets used here include the Bromodomain Adjacent to Zinc Finger Domain 2B (BAZ2B), Apoptosis regulator Bcl-2; Apoptosis regulator Bcl-xL, BRD4 bromodomain 1 BRD4-1, CREB-binding protein (CREBBP), HIV Integrase (HIV IN), Inhibitor of apoptosis protein 3 (XIAP), Induced myeloid leukemia cell differentiation protein Mcl-1, and E3 ubiquitin-protein ligase Mdm2, and Menin (Table 1, Figure S2). The Ephrin type-A receptor 4 (EphA4) was excluded from this work as there were not sufficient compounds to build ML models. The vs. performance of rescoring and ML models was assessed on two protein structures per target, which were selected based on the diversity of the binding sites (Figure S2). The target compounds were collected from the ChEMBL [48] and PubChem [49] databases. The chemical space of the PPI datasets was analyzed by performing Principal Component Analysis (PCA) using simple physicochemical properties of the compounds (Figure S3). For more details on the methodology used for selecting the protein structures, curation of target datasets, and PCA analysis, see ref. [50].

### 2.1. Virtual Screening Performance of GOLD Scoring Functions

We observed in our previous study that the fast “pscreen” Surflex docking approach, on PPI targets, displayed variable screening performances with excellent AUC values (>0.8) for 3 targets (Bcl-2, Bcl-xl, XIAP); moderate AUCs (0.6–0.7) for four targets (BRD4-1, CREBBP, HIV IN, Mdm2); and poor AUC values (0.4–0.6) for four targets (BAZ2B, Mcl-1, Menin and EphA4) [50]. We now wanted to investigate the performance of other types of re-scoring functions on this dataset. We decided to use the GOLD package for rescoring purposes as it offers different types of scoring functions. For example, the GoldScore function combines force-field terms with empirical terms to account for some of the deficiencies in pure force-field-based scoring functions [51]. ASP is a knowledge-based scoring function that uses information about the frequency of interaction between ligand and protein atoms. It is gathered by analyzing existing ligand-protein structures in the PDB and this information is then used to generate the statistical potentials. ChemPLP and ChemScore are pure empirical scoring functions that estimate the binding affinity of a complex based on a set of weighted energy terms whose coefficients are determined by fitting the binding affinity data of a training set of protein–ligand complexes with known 3D structures, using least squares fitting (see Methods for more detail on GOLD scoring functions). Table 2 shows the comparison of vs. performance between Surflex and different GOLD scoring functions (GoldScore, ChemScore, ASP, ChemPLP, ChemScore RDS) in terms of AUC and BEDROC for the different PPI targets. The AUC calculations were performed after sorting the global pose space and retaining the best pose for each compound after rescoring with GOLD scoring functions and via a consensus approach. Table S1 shows the corresponding early enrichment values for the targets at 1% and 5% levels for the best-predicted poses obtained from rescoring the global pose space. The rescoring of the different data sets revealed that among all the GOLD scoring functions, ASP and GoldScore displayed the best results by yielding higher AUC values for six targets (with a total of 11 protein structures) and seven targets (10 protein structures), respectively, as compared to Surflex. Importantly, ASP generated significantly improved and highest AUC values for four protein structures (Bcl-2: 2O21; BRD4-1: 5D3L, 5KU3; CREBBP: 5EIC), while GoldScore produced the best AUC values for three structures (Mcl-1: 5MES, Mdm2: 4ODF, 4ZFI). ChemPLP and consensus were the next good performing scoring functions generating higher AUC values than Surflex for eight and nine protein structures respectively. The consensus approach generated an AUC of 0.66 in 5FC4 (Mcl-1), which was the highest among all scoring functions. The BEDROC calculations showed that ChemPLP was the best scoring function producing higher values for 12 target protein structures, followed by GoldScore and consensus scoring for 11 structures as compared to Surflex. ASP and ChemScore generated higher BEDROC values for nine protein structures. GoldScore produced the BEDROC values with a difference greater than 0.05 (ΔBEDROC) for two structures (Mcl-1: 5FC4, 5MES) as compared to Surflex and with a ΔBEDROC of >0.1 for three structures (BRD4-1: 5D3L, 5KU3, Mdm2: 4ZFI), indicating good early recovery of the actives. Similarly, ChemPLP produced ΔBEDROC > 0.05 for two target structures (Bcl-2: 2O21, Mcl-1: 5FC4) and ΔBEDROC > 0.1 for three structures (Bcl-xL: 3WIZ, BRD4-1). Consensus scoring produced a ΔBEDROC of >0.05 for three structures (Bcl-xL: 3WIZ, Mcl-1: 5FC4, CREBBP: 5EIC) and ΔBEDROC > 0.1 for two structures (BRD4-1). Overall, all rescoring functions produced a ΔBEDROC > 0.1 in BRD4-1 indicating a significantly improved performance compared to Surflex on this PPI dataset. In the case of 5KU3, the rescoring functions produced more than a two-fold improvement in the BEDROC score.

Consistent with the preceding observations, ChemPLP and consensus scoring produced high enrichment values in six targets with a total of 12 and 10 protein structures at a 1% level, respectively, as compared to Surflex. Whereas, GoldScore and ASP produced a high EF1% for 6 targets (nine protein structures) and five targets (nine structures), respectively. The results at the 5% level were much better with GoldScore, ChemPLP, and consensus scoring. They all produced EF values better than Surflex in seven targets (ten protein structures for GoldScore, twelve for ChemPLP, and eleven for consensus). Whereas ASP and ChemScore generated high enrichment values for four targets (eight protein structures) and five targets (eight structures), respectively. There were some target proteins for which the enrichment factors were more than three times higher as compared with those obtained with Surflex. For example, Menin (6B41) has a ≥3-fold increase in its EF1 % when rescored with GoldScore, ASP, and ChemPLP. In Mcl-1 (5MES), EF 1% is more than two times using GoldScore. Similarly, in the case of 5KU3 (BRD4-1), GoldScore, ASP, and consensus scoring produced EF 5% > 3-fold, while in the structure 5D3L, EF1% was >2-fold using ChemScore RDS. Overall, it can be summarized that rescoring the Surflex-generated poses with GOLD scoring functions can be advantageous for some targets. In particular, GoldScore and ASP generated highly improved performances in targets, such as Mcl-1, Mdm2, BRD4-1, and Bcl-2, while for other targets, the AUCs were comparable to or slightly better than Surflex. Nevertheless, like Surflex, these scoring functions tend to also be target-dependent.

### 2.2. Post-Docking Derivatization of SASA Descriptors

We showed previously that the relative SASA (rSASA) (see Section 4.2) of the ligand could serve as a robust descriptor for identifying true PPI ligands, which are largely solvent-exposed at the binding interface. The rSASA showed good vs. performance when used independently or in combination with the Surflex scoring function [50]. Encouraged by these results, we set out to explore the capability of several diverse protein, ligand, and protein–ligand SASA descriptors in modulating vs. performance. These descriptors were calculated by measuring the SASA of the receptor–ligand complex (the “bound” surface area), the receptor on its own, and the ligand on its own (the “unbound” surface areas”), and then calculating the difference in surface area between the bound complex and the unbound receptor and ligand (the “delta” surface area). No changes are made to the structures, so relaxation on binding is not included in the calculations. The SASA of the ligand and receptor before and after the receptor–ligand binding can be broken down into different categories based on either pharmacophore types, residue types, or structural features. Following the pharmacophoric approach, the SASA in the specified state of all receptor or ligand atoms within the system is divided individually into six basic types: Hydrophobic (H), Aromatic (Ar), donor (D), Acceptor (Ac), Positive (P), Negative (N). Those atoms not matching any of the types were classified as the type “other” (O). By using this approach, we extracted the seven SASA descriptor subtypes for the ligand and the receptor in the bound and unbound state. We next calculated the delta and relative SASA for each SASA category for the receptor and the ligand. In the end, four global ligand and receptor SASA descriptors were derived: total (bound and unbound), total delta, and relative total (see Methods). In this way, we derived

**32 receptor SASA descriptors**: total unbound (T_RU_), total bound (T_RB_), total delta (ΔT_R_), relative total (rel. T_R_), hydrophobic unbound (H_RU_), hydrophobic bound (H_RB_), hydrophobic delta (ΔH_R_), relative hydrophobic (rel. H_R_), aromatic unbound (Ar_RU_), aromatic bound (Ar_RB_), aromatic delta (ΔAr_R_), relative aromatic (rel. Ar_R_), donor unbound (D_RU_), donor bound (D_RB_), donor delta (ΔD_R_), relative donor (rel. D_R_), acceptor unbound (Ac_RU_), acceptor bound (Ac_RB_), acceptor delta (ΔAc_R_), relative acceptor (rel. Ac_R_), positive unbound (P_RU_), positive bound (P_RB_), positive delta (ΔP_R_), relative positive (rel. P_R_), negative unbound (N_RU_), negative bound (N_RB_), negative delta (ΔN_R_), relative negative (rel. N_R_), other unbound (O_RU_), other bound (O_RB_), other delta (ΔO_R_), relative other (rel. O_R_);

**32 ligand SASA descriptors**: total unbound (T_LU_), total bound (T_LB_), total delta (ΔT_L_), relative total (rel. T_L_), hydrophobic unbound (H_LU_), hydrophobic bound (H_LB_), hydrophobic delta (ΔH_L_), relative hydrophobic (rel. H_L_), aromatic unbound (Ar_LU_), aromatic bound (Ar_LB_), aromatic delta (ΔAr_L_), relative aromatic (rel. Ar_L_), donor unbound (D_LU_), donor bound (D_LB_), donor delta (ΔD_L_), relative donor (rel. D_L_), acceptor unbound (Ac_LU_), acceptor bound (Ac_LB_), acceptor delta (ΔAc_L_), relative acceptor (rel. Ac_L_), positive unbound (P_LU_), positive bound (P_LB_), positive delta (ΔP_L_), relative positive (rel. P_L_), negative unbound (N_LU_), negative bound (N_LB_), negative delta (ΔN_L_), relative negative (rel. N_L_), other unbound (O_LU_), other bound (O_LB_), other delta (ΔO_L_), relative other (rel. O_L_). 

With the protein and ligand SASA descriptors in hand, we derived **16 protein–ligand SASA descriptors** for each SASA category: total delta (ΔT_RL_), relative total (rel. T_RL_), hydrophobic delta (ΔH_RL_), relative hydrophobic (rel. H_RL_), aromatic delta (ΔAr_RL_), relative aromatic (rel. Ar_RL_), donor delta (ΔD_RL_), relative donor (rel. D_RL_), acceptor delta (ΔAc_RL_), relative acceptor (rel. Ac_RL_), positive delta (ΔP_RL_), positive (rel. P_RL_), negative delta (ΔN_RL_), relative negative (rel. N_RL_), other delta (ΔO_RL_), relative other (rel. O_RL_) (see methods).

### 2.3. Virtual Screening Performance of SASA Descriptors

**Figure 1:**
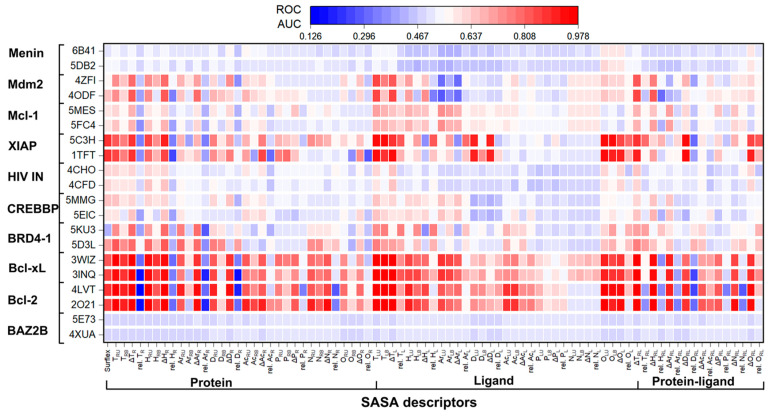
Heat map plot showing the AUC values for the ten PPI targets and their protein structures (*y*-axis) obtained using 32 protein SASA descriptors, 32 ligand SASA descriptors, and 16 protein-ligand SASA descriptors. The calculations were performed for each target-specific set of active and inactive binders.

The AUC values calculated for the PPI datasets screened using SASA descriptors are shown as a heat map in Figure 1 and Table S2 in the supporting information. The combined analysis of AUCs for the different descriptors is shown in Figure S4. The percent change in the AUC values, BEDROC scores, and database enrichments EF1%/EF5% are shown in Tables S3–S6, respectively. The results indicate that many post-docking calculated SASA descriptors of proteins, ligands, and protein–ligand complexes generated AUCs that were comparable to or better than the default Surflex scores in many targets. For instance, among the receptor SASA descriptors, T_RU_, ΔT_R_, ΔH_RU_, ΔH_R_, and ΔAr_R_ were the best descriptors that achieved excellent performance (AUC > 0.8) in seven, nine, six, eight, and five protein structures, respectively, and reasonably good performance (AUC: 0.7–0.8) in another three, one, four, two and three target structures, respectively. Bcl-2, Bcl-xL, BRD4-1, XIAP, and Mdm2 were the common test systems in which T_RU_, ΔT_R_, ΔH_RU_, and ΔH_R_ descriptors showed good performance. Whereas, ΔAr_R_ showed excellent performance in the aforementioned targets except for XIAP. When compared to the performance of Surflex, T_RU_, ΔT_R_, H_RU_, ΔH_R_, ΔAr_R_, D_RU_, ΔD_R_, Ac_RU_, and ΔAc_R_ generated improved AUC values in 12, 13, 14, 15, 11, 10, 10, 10, and nine protein structures respectively (Table S3). Among the ligand SASA descriptors, T_LU_, T_LB_, ΔT_L_, O_LU_, O_LB_, and ΔO_L_ achieved excellent performance (AUC > 0.8) in 8, 6, 8, 6, 6, and 6 protein structures, respectively. Bcl-2, Bcl-xL, BRD4-1, XIAP, and Mdm2 were the common targets in which these ligand descriptors performed very well. However, the performance of O_LU_, O_LB_, and ΔO_L_ was moderate in the targets BRD4-1 and XIAP. When considering overall performance, T_LU_, T_LB_, ΔT_L_, H_LU_, O_LU,_ O_LB,_ and ΔO_L_ were the best descriptors that generated higher AUC values as compared to Surflex in 15, 15, 16, 11, 11, 12, and nine protein target structures, respectively (Table S3). Among the receptor–-ligand SASA descriptors, ΔT_RL_, ΔH_RL_, ΔD_RL_, and ΔO_RL_ produced excellent results (AUC > 0.8) in eight, five, six, and six protein structures, respectively. Whereas ΔT_RL_ and ΔH_RL_ showed good performance in another target structure. Bcl-2, Bcl-xL, and XIAP were again the common targets in which ΔT_RL_, ΔD_RL_, and ΔO_RL_ showed improved performance. ΔT_RL_ also showed high performance in BRD4-1, while ΔH_RL_ showed excellent results in Bcl-2, Bcl-xL, and BRD4-1. Based on the results obtained against all targets, it can be inferred that ΔT_RL_, ΔH_RL_, ΔA_RL_, ΔD_RL_, ΔN_RL_, and ΔO_RL_ were the best descriptors generating higher AUC values as compared to Surflex in 16, 12, 10, 10, nine, and nine protein structures respectively. Based on the average AUCs over the entire dataset, the following trend was observed: T_LU_ > ΔT_R_ > ΔT_RL_ > ΔH_R_ > ΔT_L_ > T_RU_ > T_LB_ > H_RU_ > O_LU_ > ΔH_RL_ > O_LB_ > ΔAr_R_ > Surflex > others (Figure S4). Besides the good vs. performance, as evident by the enhanced AUC values (Figure 1 and Table S2), the SASA descriptors-based scoring metrics also exhibited high early enrichments and BEDROC scores in comparison to Surflex. The SASA descriptors T_RU_, ΔT_R,_ H_RU_, ΔH_R_, T_LU_, T_LB_, ΔT_L_, O_LU_, O_LB_, ΔT_RL_, ΔH_RL_, and ΔAr_RL_ generated higher EF 1% values in eight targets (with a total of 12 protein structures), eight targets (11 structures), six (nine structures), eight targets (11 structures), nine targets (14 structures), eight targets (11 structures), eight targets (12 structures), six targets (nine structures), seven targets (10 structures), eight targets (12 structures), seven targets (10 structures), and five targets (nine structures), respectively. There were some target proteins for which the EF1% is 2–6 times higher than those obtained with Surflex. For example, Mcl-1 (5MES) has a ~4-fold increase in its EF1% for T_LB_, ΔT_RL_; and >4-fold and >5-fold when scored using T_R_, and T_LU_, respectively. Similarly, Menin (6B41) has a >4-fold increase in its EF1% for T_R_ and D_R_. Whereas, T_RU_, H_R_, Ac_RU_, Ac_R_, N_R_, T_LU_, T_L_, O_LU_, ΔO_L_, and ΔO_RL_ produced increased enrichment (between 3–4%) for the same protein. In the case of BRD4-1 (5KU3), rel. H_L_ showed a ~3-fold increase in EF1% value. T_R_ and ΔT_RL_ produced ~2 times higher EF1% value for Bcl-xL (3INQ). When considering EF5%, T_RU_ showed higher enrichment values in six targets (with a total of 11 protein structures); ΔT_R_ in eight targets (14 protein structures); H_RU_ in six targets (11 structures); ΔH_R_ in eight targets (14 structures); ΔAr_R_ in six targets (10 structures); T_LU_ in eight targets (13 structures); T_LB_ in eight targets (12 structures); T_L_ in seven targets (11 structures); O_LB_ in six targets (11 structures); T_RL_ in eight targets (13 structures); and ΔH_RL_ in eight targets (12 structures). There were some target proteins for which the EF5% is up to four times higher than those obtained from Surflex docking. For example, rel. H_L_ showed a four-fold increase of EF5% in BRD4-1 (5KU3). In the case of Bcl-xL (3WIZ), T_LU_ generated EF5% ~2-fold higher as compared to Surflex. T_LU_ and T_LB_ generated more than two-fold higher EF5% in 5MES (Mcl-1). Whereas ΔT_R_, ΔT_R_, and ΔT_RL_ produced EF5% > two-times in Menin (6B41). Collectively, among all descriptors, T_RU_, ΔT_R_, ΔH_R_, T_LU_, T_LB_, ΔT_L_, O_LU_, and ΔT_RL_ were the major SASA scoring metrics that produced higher average EF1% and EF5% values as compared to Surflex (Figure S5). The vs. performance in terms of BEDROC score (α = 20) for the SASA descriptors showed a similar trend as that observed for the early enrichment factors (Figure S6). There were some target proteins for which the BEDROC scores were 2–3 times higher than those obtained from the default Surflex-dock scores. For instance, H_LB_ and rel. H_L_ generated BEDROC score ~3 times higher for BRD4-1 (5KU3) as compared to Surflex. T_R_, T_LU_, T_LB_, and T_RL_ increased the BEDROC value by ~2-fold in Mcl-1 (5MES). While T_LU_ produced ~2 times higher BEDROC score in the case of 5FC4 (Mcl-1). Thus, it can be deduced that the performance of some topological descriptors is superior to Surflex as well as GOLD rescoring functions. This suggests that these 3D descriptors could be used as scoring metrics for screening compound collections after performing docking and for instance a first rescoring post-processing step.

### 2.4. Building and Validating Machine Learning Models Using SASA Descriptors

To investigate whether it is possible to discriminate the two classes of PPI inhibitors (active: 1 and inactive: 0) using the ML approaches, we built several classification ML models relying on the derivatized SASA descriptors from the docking poses for each PPI target and the corresponding protein structures. The dataset (poses) of each target was split into a training set (70%) and a test set (30%) in a stratified fashion to have the same proportion of class labels in the training and test subsets as the input data set (Table 1). We selected eight binary classifiers, tree, bagged forest, random forest, Bayesian, SVM, logistic regression, neural net, and neural net (with bagging) implemented in BIOVIA pipeline pilot v18.1 software. The models were constructed with default hyperparameters. The model performance was assessed by computing ROC AUC, sensitivity, specificity, precision, and concordance (accuracy) for the training and test sets. Table 3 shows the 10-fold cross-validation ROC AUC values attained for ten PPI targets using eight ML methods. Other model statistics obtained from the cross-validation are provided in Table S7. Based on the results, the neural network (bagging) outperformed all ML techniques by producing the highest cross-validated AUC values in nine out of 10 targets (with a total of 17 protein structures). SVM produced the best AUC values in both structures of BAZ2B and in one structure of CREBBP (5MMG). Both neural network (bagging) and SVM were equally effective in the case of 3WIZ (Bcl-xL) and 5EIC (CREBBP). Similarly, random forest and neural network (bagging) showed the best performance in the case of Bcl-2 (2O21). Taken together, neural net (bagging), random forest, and SVM emerged as the best classifiers by producing AUC > 0.8 in 18 structures; Bayesian and neural network in 14 structures; and tree, bagged forest, logistic regression in 12 structures. Table 4 shows the AUC values obtained by the different models on the test sets. Whereas other statistics obtained on test sets are provided in Tables S8 and S9. Neural network with bagging produced the best AUC values in eight out of 10 targets with a total of 14 structures. The random forest algorithm produced the best AUC values in two targets (Bcl-2 and Menin; four structures). Tree with or without bagging generated the highest AUC value in BRD4-1 (5D3L). The neural network without bagging showed the highest AUC values in Bcl-xL (3INQ) and XIAP (5C3H). With respect to overall performance, neural networks with bagging achieved excellent performance (AUC > 0.8) in 14 structures; random forest in 13 structures; trees with bagging, SVM, and logistic regression, and neural network without bagging in 12 structures each, tree without bagging in 11 structures; and Bayesian in 10 structures. Considering the performances of ML classifiers on the test sets, the different algorithms can be graded in the following order: neural network (bagging) > random forest > bagged forest ~ SVM ~ logistic regression ~ neural network > tree > Bayesian. A similar trend can be deduced when the sensitivity, specificity, precision, and concordance data are compared across the different ML models. For instance, the neural net (bagging) model produced sensitivity > 80% in six targets (with a total of 12 protein structures). SVM, Bayesian, random forest, and neural network produced sensitivity > 0.8 in six targets (11 structures), five targets (10 structures), five targets (nine protein structures), and five targets (nine structures), respectively (Table S8). However, most of the ML models failed in terms of sensitivity in the targets BAZ2B and Menin.

### 2.5. Enrichment Factors Estimation of Machine Learning Models

All of the ML models were used to calculate the enrichment factors (EF1% and EF5%) for the test sets (Table S10). Consistent with the highly improved AUC values, the ML models also showed high early enrichment values for the test sets in several target proteins. In the case of BAZ2B, the best enrichment in the hit rate for the top-scoring 1% of compounds was 2 for the protein 4XUA produced by neural net (bagging), and for 5E73 SVM produced EF1% of 2.2. For Bcl-2 (2O21), random forest, SVM, and neural networks were equally effective by producing the best EF1% value of 28.72. In the case of 4LVT, these models, including Bayesian, produced the same EF1% value of 28.72. For Bcl-xL (3INQ), neural network (bagging) produced the best EF1% of 52. While in the case of 3WIZ, neural networks and random forest produced the same EF1% value. For BRD4-1 (5D3L), all ML models, except Bayesian, produced the EF1% of 2.162. In comparison, all models produced the same result in the case of 5KU3. For CREBBP, the neural network (bagging) exhibited the best EF1% value of 12.38 and 9.03 in 5EIC and 5MMG, respectively. For HIV IN, all methods exhibited the EF1% value of 2.4 in 4CFD. While in 4CHO, bagged forest, random forest, and neural networks showed the same EF1% value. For XIAP, random forest and neural network (bagging) produced the best EF1% value of 43.934 in 1TFT and 5C3H. For Mcl-1, SVM and neural network (bagging) displayed the best enrichment of 23.805 and 23.57 in 5FC4 and 5MES, respectively. For Mdm2, all models, except Bayesian and logistic regression, exhibited the EF1% value of 2.9. In the case of 4ZFI, random forest, SVM, logistic regression, and neural network (without bagging) produced the same EF1%. For Menin, SVM produced the best EF1% value of 6.68 in both 5DB2 and 6B41. Overall, the best performance was exhibited by the neural network by yielding the best EF1% value in nine targets with a total of 14 structures and an average EF1% of 17.23. The second-best ML method was SVM which produced the best EF1% value in seven targets with a total of 11 structures and an average EF1% of 17.01. Random forest stood in third place by producing the best EF1% value in seven targets with a total of 10 structures and an average EF1% of 16.8. With respect to the enrichment factor at 5%, the neural network (bagging) outperformed other techniques by producing the best EF5% value in all targets with a total of 14 structures and an average EF5% of 8.5. Random forest was the second-best method that generated the high EF5% value in six targets with a total of seven structures and an average EF5% value of 8.3. SVM produced a high EF5% value in four targets (four structures) and an average EF5% of 8.134. Our analysis of additional test statistics (Cohen’s Kappa κ, Matthew’s correlation coefficient MCC, Youden’s Index J, and F1 score) is in agreement with the conclusions drawn here. For example, the Neural network with bagging performed better than other methods based on the aforementioned parameters in most of the targets. Whereas, Bayesian and SVM produced some improvement in the targets BAZZ2B and Menin, in which other methods failed (Figure S7). We also compared the early enrichments of ML models to Surflex and the best-performing receptor, ligand, and receptor–ligand SASA descriptors (Figure S8). The overall mean of EF1% and EF5% for the ML models were much higher than the Surflex and SASA descriptors. For instance, neural network (bagging) models produced EF1% ~7 times better as compared to Surflex in targets Mcl-1 (5FC4) and CERRBP (5EIC). Whereas the early recovery values obtained using SASA descriptors were better than Surflex. These results indicate that the docking scoring function may not be efficient when used solely for performing VS. Rather, the use of docking pose-derived descriptors in combination with ML techniques could be valuable in identifying active PPI ligands. We also analyzed the predictor fields to understand which features matter most and which are of least importance based on the relative importance of each predictor in estimating the model. Figure S9 shows the distribution of descriptor importance values obtained from the 20 neural net (bagging) models trained on different data sets. ΔH_R_, ΔT_L_, ΔT_R_, T_RU_, ΔT_RL_, H_RU_, T_LU_, T_LB_, T_RB_, and ΔH_RL_ descriptors were predicted to be of high importance based on the Neural net models. All these descriptors also showed good vs. performance when assessed independently.

## 3. Discussion

With the discovery of novel therapeutically important PPIs, there is an urgent need to develop drug candidates modulating these interactions to supplement the currently limited clinical pipeline. Alternatively, small molecules that perturb specifically some PPIs could be valuable to understand the importance of a protein complex in a disease state and thus help de-risk target selection. However, the rational design of selective and potent small molecule inhibitors of PPIs is in general very challenging compared to traditional targets, such as kinases, proteases, or G protein-coupled receptors [52,53,54]. The present study is built on our previous fast docking and rescoring-based vs. investigation performed on inhibitors of PPIs and aims at gaining insights about the pertinence of using hybrid methods that combine docking pose-derived SASA descriptors with scoring functions and/or with ML techniques (i.e., when enough data are available for the target of interest) so as to facilitate the discovery of novel PPI inhibitors. This work is also encouraged by the research performed by Núñez et al. in which they applied a similar strategy to traditional drug targets: adenosine deaminase, and estrogen receptor alpha [55]. In that study, after docking with GOLD and Glide, they post-processed the poses to derive a protein–ligand interaction fingerprint (PLIF) metric. Next, the SASA descriptors were computed for each ligand and its respective protein in their bound/unbound states. Subsequently, a Bayesian model was learned with SASA descriptors which was then used to score the remaining ligands in the screening databases. The performance of SASA descriptors was found comparable or superior to those of GOLD and Glide. 

Here, we compared the results of a fast Surflex docking-scoring protocol against rescoring with five GOLD scoring functions. In addition, we evaluated the screening potential of docking-pose derived protein-, ligand-, and protein–ligand-SASA descriptors as well as that of ML models developed with these descriptors while monitoring the impact on (early) enrichment factors. Similar to Surflex, GOLD scoring functions were also found to be target-dependent. Among all GOLD scoring functions, GoldScore produced the best AUC value in one protein structure of Mcl-1 (5MES) and both structures of Mdm2. ASP generated the best results in Bcl-2 (2O21), BRD4-1, and CREBBP (5EIC). The performance of other GOLD scoring functions was lower than the one of Surflex. In contrast to traditional scoring functions, the overall performance and early database enrichments for many SASA descriptors were found superior to Surflex or GOLD scoring functions for a large variety of target classes suggesting that these scoring metrics could be interesting for structure-based screening experiments. For instance, among the protein SASA descriptors, SASA receptor total delta ΔT_R_ was the best-performing descriptor that produced excellent AUC values in nine protein structures. This indicates that the difference in the unbound and the bound SASA area of the binding site is a unique metric for each ligand that could be used to discriminate actives from inactive molecules. Among the ligand SASA descriptors, SASA ligand total unbound T_LU_ produced the best results in eight target structures. This is consistent with previous studies which suggested that the rSASA score of the ligand, which is derived from the bound and the unbound fraction of the ligand SASA, can be used for finding ligands that are largely solvent-exposed at the PPI sites [41,50]. The rSASA of the active PPI ligand is relatively lower as compared to that of the inactive compound. Among the protein–ligand SASA descriptor, SASA receptor–ligand total delta ΔT_RL_ generated excellent results in eight target structures. This suggests that the net SASA of the protein–ligand complex is another distinctive feature of an active ligand that can be valuable for screening purposes. The vs. performance was further enhanced when we used the descriptors for training ML models and validated them on test sets. Among the ML models and the present PPI dataset, neural networks and random forest achieved the best results in most of the targets except BAZ2B, for which all methods could not yield desirable results. In the case of Menin, the results were only satisfactory using random forest models. The visual inspection of the binding sites of BAZ2B structures showed relatively tight subpockets that are formed by hydrophobic/aromatic residues and with a conserved water molecule. In the case of Menin structures, the binding site is large and substantially flatter compared to the other PPIs. These features pose a significant challenge in ligand docking and it is possible that the poses generated for the compounds on these targets are not accurate enough. This in turn leads to the calculation of incorrect SASA descriptors that could not help train good ML models. Importantly, a consistent trend is observed where all methods (native docking, rescoring, SASA metrics, and ML models) failed on these targets which likely points to the fact that docking poses are unreliable and/or that some possible experimental errors could be present. 

As more information is being used when developing ML models as compared to only relying on a docking score, as expected, the results obtained from the ML approaches are superior to the best-rescoring function DLIGAND2 identified in our last study [50] both in terms of AUC and early enrichments. Additionally, we would like to emphasize that in the future we would be working in a more flexible ML platform that could allow automatic tuning of hyperparameters. Since we are dealing with large datasets, different targets, and ML techniques, it was computationally expensive to manually explore all hyperparameters in Pipeline Pilot. However, it became clear from our experiments that adopting default hyperparameters showed a consistently good performance in most of the targets. Yet we anticipate that the performances observed could be further improved (particularly sensitivity and early enrichments) after tuning hyperparameters. 

In trying to rationalize why SASA descriptors would help in discriminating between good and bad binders, we already mentioned that the method does not seem to favor high MW molecules [50]. In fact, it would seem that the SASA descriptors as used here assist the selection of molecules that fill better the binding site most likely important for PPIs as it has been shown that ligand binding pockets within a PPI interface are very different from traditional ligand binding pockets and tend to contain three to five binding sub-pockets of low volume [56,57]. It is also possible that SASA descriptors are fuzzier than interaction fingerprints or scoring functions and as such less sensitive to possible docking errors likely to occur due to the plasticity of most PPI interfaces. Thus, they can be interesting to combine with such approaches while important work will be required to fully investigate this question. For the time being, combining SASA descriptors with rescoring is still target-dependent, but the present results suggest that the approach could be interesting to circumvent in part this problem.

The protocol presented here, which involves docking compounds, extracting 3D descriptors from the docking poses, and using them for building ML models, seems interesting to investigate further with regard to the generation of compound collections potentially enriched in PPI modulators. We suggest that pose-derived SASA descriptors or pose-derived SASA descriptors combined with ML techniques could be a useful adjunct to other methods. For example, most of the work done so far to design dedicated PPI collections are mainly ligand-based. For instance, Hamon et al. built an SVM model with DRAGON molecular descriptors for small molecules using the 2P2I database (https://2p2idb.marseille.inserm.fr, accessed on 14 November 2022) to define a physicochemical profile of orthosteric inhibitors. The model was successfully used to mine PPI-like compounds from external datasets from PubChem BioAssay and in-house small molecule collections [47]. In another study, Reynes et al., by constructing decision trees on a dataset of known PPI inhibitors and regular drugs, determined a global physicochemical profile for the putative PPI inhibitors. The statistical analysis revealed two important molecular descriptors for PPI inhibitors characterizing specific molecular shapes and the presence of a privileged number of aromatic bonds. The best model was transposed into a computer program, PPI-HitProfiler, that can output from any drug-like compound collection a focused chemical library enriched in putative PPI inhibitors [58]. In a recent study, Bosc et al. constructed different ML models based on molecular descriptors using databases of experimentally confirmed PPI inhibitors. The predictive models were used to curate the putative PPI inhibitors from large commercial compound collections. The curated collection is available on-demand to the scientific community in 384-well plates as the Fr-PPIChem library. The compounds from the library showed a 46-fold activity rate enhancement compared to a non-iPPI-enriched diversity library in high-throughput screening against the CD47-SIRPα PPI [59]. It seems possible that SASA descriptors generated from docking poses could also be valuable to assist the design of PPI collections. 

In summary, we showcased a proof-of-concept study highlighting the potential of GOLD scoring functions; several post-docking derivatized SASA descriptors of protein, ligand, and protein–ligand complex; and ML models to enhance the performance of fast docking computations performed with Surflex (“pscreen” mode) as monitored by ROC AUC and early database enrichment efficiency using experimentally known inhibitors of PPI targets. Significantly higher AUC values and enrichment factors were obtained for several targets using 3D SASA descriptors combined with rescoring or combined with ML as compared to default “pscreen” Surflex computations or rescoring using only GOLD functions, indicating an improvement in both the global ranking of compounds and also, in the early enrichment stages. The latter significant issue is additionally manifested in ligand enrichments at 1%, which were augmented by up to seven times in several proteins using neural network models. Collectively, our results strongly encourage the use of docking in combination with the use of pose-derived SASA descriptors or with ML techniques when a sufficient number of bioactivity data are available, to screen large molecular databases in search of new PPI inhibitors.

## 4. Materials and Methods

### 4.1. Rescoring with GOLD Scoring Functions and Consensus Approach

The procedure of docking PPI datasets to the respective protein structures is described in ref [50]. The docking poses obtained from each protein structure were rescored using five different scoring functions with default settings: (i) GoldScore: It is the original scoring function provided with GOLD [51,60]. It has been optimized for the prediction of ligand binding positions and takes into account factors such as hydrogen bonding energy, van der Waals energy, and ligand torsion strain; (ii) ChemPLP: piecewise linear potential is an empirical fitness function optimized for pose prediction. The piecewise linear potential (PLP) is used to model the steric complementarity between protein and ligand, but additionally, in ChemPLP, the distance- and angle-dependent hydrogen and metal bonding terms from Chemscore are considered. ChemPLP is slightly faster than ChemScore and up to four times faster than GoldScore. Several validation tests have shown it to be generally more effective than the other scoring functions for both pose prediction and virtual screening; (iii) Astex Statistical Potential (ASP): ASP is an atom-atom potential derived from a database of protein–ligand complexes and can be compared to other knowledge-based scoring potentials, e.g., PMF and DrugScore. ASP has comparable accuracy to ChemScore and GoldScore; (iv) ChemScore: It estimates the total free energy change that occurs on ligand binding and was trained by regression against binding affinity data for 82 complexes. The ChemScore fitness function also incorporates a protein–ligand atom clash term and an internal energy term. ChemScore takes into account hydrophobic-hydrophobic contact area, hydrogen bonding, ligand flexibility, and metal interaction; and (v) ChemScore (RDS), a modified form of ChemScore where the effect of scaling hydrogen bonds, metal–ligand, and hydrophobic interactions are systematically investigated based on the burriedness of intermolecular interactions in protein–ligand complexes. Burriedness is expressed using receptor density scaling (RDS), as defined by the number of heavy atoms around an interaction. Optimization of scaled ChemScore terms using burriedness from experimental X-ray data and docked inactive ligands has shown to improve the ability to discriminate actives from inactives significantly. These modified ChemScore terms penalize interactions with flexible outer walls or loops of the binding site; they adjust hydrogen-bond strengths in solvent-exposed surface areas when there is competition with solvent for favorable hydrogen bond interactions [51]. 

Consensus scoring is a method in which the predicted binding affinities or scores of each compound for a binding pocket are predicted by using more than one scoring method. In this study, a consensus scoring approach using the ‘rank-by-rank’ method [33] was applied to evaluate the hits obtained from high throughput docking. All the candidates were ranked by the average ranks predicted by all the scoring functions. This strategy uses relative ranks rather than absolute binding affinities for ranking. Since the compound docking scores obtained from Surflex and GOLD rescoring are of different nature and type, data normalization was performed to bring all the scores to a common scale ranging from 0 to 1. Data normalization was performed using Equations (1) and (2).

(1) $$Normalized\;score=\frac{Docking\;score-Docking\;score_{min}}{Docking\;score_{max}-Docking\;score_{min}}\;$$

The compounds were then ranked based on the normalized docking scores from the five scoring functions. Finally, the results from different scoring functions were combined by averaging the rank of each molecule obtained from the individual scoring function (Equation (2)). These consensus scores were then utilized for rescoring and vs. assessment. The compounds were ranked from best to worst based on their consensus rank.

(2) $$Consensus\;Rank=\frac{Surflex_{rank}+GoldScore_{rank}+PLP_{rank}+ASP_{rank\;}+ChemScore_{rank}+ChemScore-RDS_{rank}}{6}\;$$

### 4.2. Solvent Accessible Surface Area (SASA) Calculations and Derivatization of SASA Descriptors

The SASA of the ligand and corresponding receptor-binding site in their bound/unbound states was calculated for each docking pose using the “binding_sasa.py” script from Schrödinger. More details on the procedure of calculation of SASA descriptors are described in ref [50]. Briefly, seven SASA descriptor subtypes (Hydrophobic (H), Aromatic (Ar), Donor (D), Acceptor (Ac), Positive (P), Negative (N), and Others (O)) were calculated for the protein and the ligand in the bound and unbound state. The protein and ligand descriptors were combined to generate the corresponding protein–ligand SASA descriptors. The total delta and relative total SASA for the receptor, ligand, and receptor–ligand were calculated as described in Equations (3)–(8). The relative SASA for each SASA category was calculated for ligand, protein, and protein–ligand using Equations (9)–(11). To evaluate the vs. performance of rescoring with GOLD scoring functions, consensus method, and SASA descriptors, we followed the protocol defined in Figure S1 for each PPI target. The performance was assessed by computing ROC graphs and calculating the AUC, EF1%, EF5%, and BEDROC (α = 20), demonstrating the ability of the vs. method to distinguish known ligands among the set of inactive compounds or predominance of active ligands in the top positions of the ranked list [61,62,63,64,65].

(3) $$SASA\;total\;ligand\;delta\;\left(T_{L}\right)=SASA\;total\;ligand\;\;unbound\;\left(T_{LU}\right)-SASA\;total\;ligand\;bound\;\left(T_{LB}\right)\;\;$$

(4) $$SASA\;total\;receptor\;delta\;\left(\Delta T_{R}\right)=SASA\;total\;receptor\;unbound\;\left(T_{RU}\right)-SASA\;total\;receptor\;bound\;\left(T_{RB}\right)\;\;$$

(5) $$\begin{array}{cc} SASA\;total\;receptor & -ligand\;delta\;\left(\Delta T_{RL}\right)\\ & =\left\{SASA\;total\;receptor\;unbound\;\left(T_{RU}\right)+SASA\;total\;ligand\;\;unbound\;\left(T_{LU}\right)\right\}\\ & -\left\{SASA\;total\;receptor\;bound\;\left(T_{RB}\right)+SASA\;total\;receptor\;bound\;\left(T_{LB}\right)\right\} \end{array}$$

(6) $$relative\;SASA\;Total\;ligand\;\left(rel.\;T_{L}\right)=\frac{T_{LB}}{T_{LU}}\;\;$$

(7) $$relative\;SASA\;Total\;receptor\;\left(rel.\;T_{R}\right)=\frac{T_{RB}}{T_{RU}}$$

(8) $$relative\;SASA\;total\;receptor\;\;ligand\;delta\;\left(rel.\;\;T_{RL}\right)=\frac{T_{RB}+T_{LB}\;}{T_{RU}+T_{LU}}\;\;\;$$

(9) $$\begin{array}{c} relative\;SASA\;X\;ligand\;\left(rel.\;X_{L}\right)\\ =\frac{X_{LB}}{X_{LU}}\;\;\;\;\left\{Where\;X:Hydrophobic\;\left(H\right),\;Aromatic\;\left(Ar\right),\;Donor\;\left(D\right),\;Acceptor\;\left(Ac\right),\;Positive\;\left(P\right),\;Negative\;\left(N\right)and\;Others\;\left(O\right)\right\} \end{array}$$

(10) $$\begin{array}{c} relative\;SASA\;X\;Receptor\;\left(rel.\;X_{R}\right)\\ =\frac{X_{RB}}{X_{RU}}\;\;\;\;\left\{Where\;X:Hydrophobic\;\left(H\right),\;Aromatic\;\left(Ar\right),\;Donor\;\left(D\right),\;Acceptor\;\left(Ac\right),\;Positive\;\left(P\right),\;Negative\;\left(N\right)and\;Others\;\left(O\right)\right\} \end{array}$$

(11) $$\begin{array}{cc} relative\;SASA & X\;receptor\;-ligand\;delta\left(\Delta T_{RL}\right)\\ & =\frac{X_{RB}+X_{LB}}{X_{RU}+X_{LU}}\left\{\begin{array}{c} Where\;X:Hydrophobic\;\left(H\right),\;Aromatic\;\left(Ar\right),\;Donor\;\left(D\right),\\ \;Acceptor\;\left(Ac\right),\;Positive\;\left(P\right),\;Negative\;\left(N\right)\;and\;Others\;\left(O\right) \end{array}\right\} \end{array}$$

### 4.3. Machine Learning Models Generation

All ML models were generated using the BIOVIA pipeline pilot v18.1 interfaced with the R package V 3.4.1 [66,67] using the recommended standard protocol. All models were developed using the same set of SASA descriptors. The SASA scores were normalized before using them for building ML models. To address the dataset imbalance, the weighting method was set to “by class” during the model’s construction. Three types of Recursive Partitioning (RP) models were constructed: tree, bagged forest, and random forest. For creating RP single decision tree models, following parameters were used: model type = single tree; Minimum samples per node = 10; Maximum tree depth = 20; Split method = Gini; Weighting Method = By Class; Maximum Knots per property = 20; Maximum look ahead depth = 0; Maximum generic depth = 0. RP Bagged forest models are an extension of the decision tree approach that minimizes errors from over-fitting by generating multiple trees and randomizing the training sets (without resampling the descriptors) that each tree utilizes to reproduce the trends in the training set. “Bagging” (bootstrap aggregation) with replacement is used to generate a new, modified version of the training set for each tree: each tree tends to get a random subset of the original training set, with ~2/3 unique compounds and ~1/3 duplicates. In the case of RP bagged forest models, ten trees (model type = Forest) were created with “bagging” with the same settings as mentioned above. In the case of the forest of random trees (random forest), 500 trees were created with “bagging” with a number of randomly preselected variables set to 9 to be considered as splitting criteria for each node. In a Random forest, when each tree is “induced” (created), it receives a different subset of the descriptors and the algorithm applies different weights on the descriptors, which can also be used in different orders in the different hierarchies of the levels of nodes within each tree. Each tree then develops a different model to reproduce its training set. Each tree’s classification of the test instance is recorded as a vote. The votes from all trees are aggregated and the test instance is assigned to the class that receives the maximum vote. We also generated SVM-based models which are based on the principle of finding a plane that best separates a description of the two classes of compounds in the training set. For complicated datasets, algorithms called kernel functions are used to transform the descriptors of a dataset into a higher dimensional space, to better separate the “good” and “bad” compounds. Support vectors are constructed that use a minimal number of the “good” and “bad” compounds to define a boundary to the hyper plane (the multi-dimensional plane that separates the transformed descriptions of the compounds in higher dimensional space), in a way that attempts to minimize error while maximizing generalizability. The parameters used were as follows: SVM-Type = C-classification; SVM-Kernel = radial; Cost = 1, 2; Weighting Method = By Class; Gamma = 0.0125. The logistic regression models were constructed using the GLM method (Generalized linear model). GLM performs a logistic regression with no bias correction. The deep neural net models were constructed with two hidden layers and each layer with 80 nodes. The learning rate of every epoch was 0.1 with a momentum of 0.9, and the maximum number of iterations for network training was 5000. To prevent the model from over-fitting, the fraction of the hidden layer to be dropped out for model training was set to 0.25. Bayesian models build a binary classifier by uniting different sets of descriptors, with different weights on each descriptor, in diverse ways, to build a model that best reproduces the known trends in the training set. The Bayesian models were constructed using the naïve Bayes algorithm. Naïve Bayes is a fast, scalable algorithm that calculates conditional probabilities for combinations of attributes and the target attribute. From the training data, an independent probability is established. This probability gives the likelihood of each target class, given the occurrence of each value category from each input variable. It is known to perform well on large datasets and has very fast processing times. In all cases, 10-fold cross-validation was used to calculate the ROC curve. In addition, we calculated several metrics, such as Sensitivity (Equation (12)), specificity (Equation (13)), precision (Equation (14)), and concordance (Equation (15)), to evaluate the performances of classifiers. Sensitivity represents the percentage of correctly identified active compounds. Specificity signifies the percentage of correctly identified inactive compounds. Concordance corresponds to the overall accuracy. Precision signifies the percentage of identified positive compounds or positive predictive value (PPV). In addition, we calculated Youden’s J statistic (Equation (16)), Matthew’s correlation coefficient (MCC) (Equation (17)), the F_1_ score (Equation (18)), and Cohen’s Kappa statistic (C_κ_ or κ) (Equation (19)). Youden’s index (J) is a performance metric that evaluates the performance of a binary classification model. Its value ranges from 0 to 1. When the value is at its minimum (i.e., zero), the model is useless. When its value is 1, there are no false negatives or false positives, and the predictions are perfect. MCC, also referred to as the phi coefficient, is a chance-corrected statistic where MCC = 1 indicates perfect agreement, MCC = −1 indicates total disagreement, and MCC = 0 indicates that the model is no better than random. The F1 score is the harmonic mean of precision (i.e., the positive predictive value hit rate) and sensitivity. The best F1 score is 1, while the worst score possible is 0. Cohen’s Kappa (κ) is a chance-corrected statistic that uses a different method to calculate the random likelihood of making correct predictions for the external set. Kappa < 0 indicates no agreement, Kappa of 0–0.2 indicates slight agreement, Kappa of 0.21–0.40 is fairly predictive, and Kappa of 0.41–0.60 indicates moderate agreement.

(12) $$Sensitivity=\frac{TP}{TP+FN}$$

(13) $$Specificity=\frac{TN}{TN+FP}$$

(14) $$Precision=\frac{TP}{TP+FP}$$

(15) $$Concordance\;\left(Accuracy\right)=\frac{TP+TN}{TP+TN+FP+TN}$$

(16) J=sensitivity+specificity−1

(17) $$MCC=\frac{\left(TP\times TN\right)-\left(FP\times FN\right)}{\sqrt{\left(TP+FP\right)\times \left(TP+FN\right)\times \left(TN+FP\right)\times \left(TN+FN\right)}}$$

(18) $$F_{1}=2\times \frac{precision\times recall}{precision+recall}$$

(19) $$\kappa =\frac{P_{o}-P_{E}}{1-P_{E}}=1-\frac{1-P_{o}}{1-P_{E}}$$

In Equation (15), true positive (*TP*) is the number of active ligands that are predicted correctly; true negative (*TN*) is the number of inactive ligands that are predicted correctly; false negative (*FN*) is the number of active ligands that are predicted as inactive molecules, and false positive (*FP*) is the number of inactive ligands that are predicted to be active. In Equation (19), *P_O_* is the relative observed agreement among raters, and *P_E_* is the hypothetical probability of chance agreement, obtained using the observed data to calculate the probabilities of each observer randomly saying each category. If the raters are in complete agreement, then *κ* = 1. If there is no agreement among the raters other than what would be expected by chance (as given by *P_E_*), *κ* ≤ 0.

## Figures and Tables

**Table 1:** Overview of the ten PPI datasets used in this study. The target names, PDB codes of target protein structures, ChEMBL ID of the targets, the number of compounds categorized as “active” (activity class: 1) and “inactive” (activity class: 0) in the curated datasets, and the number of active and inactive compounds in the training and the test set for each target (after docking of the curated datasets) used in the construction and validation of the classification ML models, are indicated.

Target Name	PDB ID	Target ChEMBL ID	N_actives_	N_inactives_	Training Set (N_actives_)	Training Set (N_inactives_)	Test Set (N_actives_)	Test Set (N_inactives_)
Bromodomain Adjacent to Zinc Finger Domain 2B (BAZ2B)	4XUA	CHEMBL1741220	6852	49,457	4794	34,598	2055	14,827
	5E73							
Apoptosis regulator Bcl-2	2O21	CHEMBL4860	1788	49,082	1240	34,343	531	14,718
	4LVT							
Apoptosis regulator Bcl-xL	3INQ	CHEMBL4625	971	49,190	674	34,420	289	14,751
	3WIZ							
BRD4 bromodomain 1 (BRD4-1)	5D3L	CHEMBL1163125	847	981	592	687	253	294
	5KU3							
CREB-binding protein (CREBBP)	5EIC	CHEMBL5747	1360	48,781	910	34,425	390	13,896
	5MMG							
HIV Integrase (HIV IN)	4CFD	CHEMBL2366505	905	1232	610	855	261	366
	4CHO							
Inhibitor of apoptosis protein 3 (XIAP)	1TFT	CHEMBL4198	1145	49,351	793	34,528	340	14,798
	5C3H							
Induced myeloid leukemia cell differentiation protein Mcl-1	5FC4	CHEMBL4361	1455	49,112	995	34,147	426	14,635
	5MES							
E3 ubiquitin-protein ligase Mdm2	4ODF	CHEMBL5023	2227	4351	1559	3040	668	1303
	4ZFI							
Menin	5DB2	CHEMBL2093861	705	31,510	491	22,020	211	9437
	6B41							

**Table 2:** The AUC and BEDROC scores (α = 20) for the ten PPI targets (BAZ2B: 4XUA, 5E73; Bcl-2: 2O21, 4LVT; Bcl-xL: 3INQ, 3WIZ; BRD4-1: 5D3L, 5KU3; CREBBP: 5EIC, 5MMG; HIV IN: 4CFD, 4CHO; XIAP: 1TFT, 5C3H; Mcl-1: 5FC4, 5MES; Mdm2: 4ODF, 4ZFI; and Menin: 5DB2, 6B41) and their corresponding protein structures obtained after rescoring of Surflex generated poses using five GOLD scoring functions (GoldScore, ChemScore, ASP, ChemPLP, and ChemScore RDS) and via consensus scheme involving all scoring functions. The best AUC and BEDROC values corresponding to each target protein (across rows) are shown in bold.

Target	PDB ID	Surflex		GoldScore		ChemScore		ASP		ChemPLP		ChemScore RDS		Consensus Scoring	
		AUC	BEDROC	AUC	BEDROC	AUC	BEDROC	AUC	BEDROC	AUC	BEDROC	AUC	BEDROC	AUC	BEDROC
BAZ2B	4XUA	0.491	0.120	0.489	0.127	**0.505**	**0.144**	0.496	0.139	0.489	0.127	0.503	0.132	0.495	0.133
	5E73	0.482	0.120	0.482	0.118	**0.507**	**0.140**	0.493	0.132	0.487	0.123	0.506	0.139	0.493	0.127
Bcl-2	2O21	0.857	0.641	0.860	0.640	0.839	0.588	0.883	0.595	0.853	**0.711**	0.590	0.209	0.798	0.548
	4LVT	0.886	0.701	0.877	0.700	0.864	0.690	0.887	0.694	0.869	**0.739**	0.665	0.368	0.886	0.722
Bcl-xL	3INQ	**0.844**	**0.523**	0.799	0.463	0.753	0.408	0.786	0.411	0.776	0.513	0.555	0.188	0.770	0.435
	3WIZ	**0.823**	0.500	0.821	0.521	0.792	0.510	0.816	0.461	0.823	**0.614**	0.541	0.174	0.805	0.573
BRD4-1	5D3L	0.696	0.672	0.778	0.777	0.725	0.808	**0.801**	0.818	0.793	**0.858**	0.671	0.852	0.799	0.849
	5KU3	0.426	0.254	0.652	0.600	0.531	0.516	**0.678**	**0.674**	0.632	0.572	0.503	0.530	0.625	0.643
CREBBP	5EIC	0.604	0.100	0.627	0.114	0.598	0.123	**0.675**	**0.159**	0.643	0.134	0.546	0.082	0.664	0.155
	5MMG	**0.662**	**0.160**	0.654	0.134	0.579	0.113	0.631	0.138	0.613	0.119	0.540	0.084	0.620	0.135
HIV IN	4CFD	**0.627**	**0.604**	0.590	0.356	0.462	0.401	0.614	0.565	0.595	0.476	0.451	0.335	0.567	0.533
	4CHO	**0.631**	**0.639**	0.617	0.425	0.473	0.371	0.620	0.589	0.614	0.537	0.455	0.335	0.571	0.521
XIAP	1TFT	**0.888**	**0.767**	0.851	0.636	0.823	0.582	0.811	0.493	0.845	0.572	0.752	0.535	0.806	0.563
	5C3H	**0.892**	**0.704**	0.676	0.341	0.726	0.492	0.765	0.502	0.778	0.632	0.739	0.551	0.735	0.626
Mcl-1	5FC4	0.585	0.170	0.636	0.228	0.657	0.257	0.658	0.212	0.642	0.243	0.595	0.223	**0.659**	0.241
	5MES	0.59	0.150	**0.630**	**0.205**	0.602	0.167	0.596	0.152	0.605	0.166	0.524	0.130	0.605	0.168
Mdm2	4ODF	0.64	0.842	**0.712**	**0.869**	0.633	0.791	0.706	0.832	0.681	0.822	0.531	0.649	0.661	0.833
	4ZFI	0.57	0.600	**0.657**	**0.744**	0.535	0.442	0.594	0.539	0.554	0.400	0.435	0.242	0.566	0.492
Menin	5DB2	**0.548**	0.104	0.534	**0.136**	0.525	0.099	0.533	0.124	0.536	0.122	0.527	0.087	0.532	0.129
	6B41	0.517	0.065	0.521	**0.104**	0.478	0.066	0.509	0.092	0.527	0.093	0.469	0.059	0.489	0.076

**Table 3:** 10-fold cross-validation ROC AUC values with their standard deviations (SD) attained for ten PPI targets obtained using different ML learning approaches. The best AUC values corresponding to each target protein (across rows) are shown in bold.

Target	PDB ID	Tree	Bagged Forest	Random Forest	Bayesian	SVM	Logistic Regression	Neural Net	Neural Net (Bagging)
BAZ2B	4XUA	0.543 ± 0.013	0.500 ± 0.000	0.601 ± 0.145	0.602 ± 0.001	**0.758 ± 0.005**	0.550 ± 0.018	0.543 ± 0.009	0.651 ± 0.024
	5E73	0.534 ± 0.010	0.500 ± 0.000	0.631 ± 0.132	0.602 ± 0.001	**0.772 ± 0.002**	0.535 ± 0.016	0.542 ± 0.004	0.671 ± 0.021
Bcl-2	2O21	0.965 ± 0.011	0.943 ± 0.001	**0.999 ± 0.117**	0.984 ± 0.000	0.990 ± 0.000	0.973 ± 0.000	0.992 ± 0.001	**0.999 ± 0.000**
	4LVT	0.948 ± 0.004	0.940 ± 0.001	0.998 ± 0.038	0.984 ± 0.000	0.988 ± 0.000	0.979 ± 0.000	0.989 ± 0.001	**0.999 ± 0.000**
Bcl-xL	3INQ	0.971 ± 0.032	0.907 ± 0.004	0.997 ± 0.129	0.983 ± 0.001	0.991 ± 0.001	0.980 ± 0.000	0.987 ± 0.001	**0.999 ± 0.001**
	3WIZ	0.973 ± 0.019	0.906 ± 0.007	0.998 ± 0.011	0.977 ± 0.000	**0.999 ± 0.001**	0.975 ± 0.000	0.988 ± 0.005	**0.999 ± 0.000**
BRD4-1	5D3L	0.962 ± 0.028	0.972 ± 0.007	0.968 ± 0.013	0.936 ± 0.001	0.952 ± 0.001	0.917 ± 0.001	0.947 ± 0.013	**0.999 ± 0.003**
	5KU3	0.972 ± 0.049	0.966 ± 0.005	0.957 ± 0.008	0.924 ± 0.001	0.927 ± 0.001	0.900 ± 0.001	0.928 ± 0.010	**0.999 ± 0.003**
CREBBP	5EIC	0.635 ± 0.042	0.500 ± 0.000	0.887 ± 0.178	0.794 ± 0.001	**0.938 ± 0.003**	0.766 ± 0.000	0.766 ± 0.011	**0.938 ± 0.033**
	5MMG	0.714 ± 0.070	0.500 ± 0.000	0.883 ± 0.153	0.746 ± 0.009	**0.946 ± 0.004**	0.734 ± 0.014	0.760 ± 0.022	0.934 ± 0.028
HIV IN	4CFD	0.970 ± 0.008	0.920 ± 0.017	0.936 ± 0.004	0.827 ± 0.004	0.895 ± 0.002	0.781 ± 0.002	0.831 ± 0.024	**0.999 ± 0.005**
	4CHO	0.931 ± 0.055	0.928 ± 0.009	0.929 ± 0.019	0.832 ± 0.014	0.896 ± 0.003	0.770 ± 0.001	0.833 ± 0.052	**0.999 ± 0.005**
XIAP	1TFT	0.972 ± 0.089	0.974 ± 0.038	0.998 ± 0.127	0.981 ± 0.000	0.997 ± 0.000	0.984 ± 0.000	0.994 ± 0.005	**0.999 ± 0.000**
	5C3H	0.985 ± 0.006	0.984 ± 0.007	0.998 ± 0.116	0.981 ± 0.003	0.998 ± 0.000	0.987 ± 0.000	0.997 ± 0.000	**0.999 ± 0.000**
Mcl-1	5FC4	0.752 ± 0.068	0.578 ± 0.006	0.930 ± 0.143	0.811 ± 0.002	0.835 ± 0.002	0.817 ± 0.001	0.842 ± 0.010	**0.956 ± 0.008**
	5MES	0.780 ± 0.089	0.583 ± 0.003	0.921 ± 0.147	0.819 ± 0.002	0.877 ± 0.004	0.817 ± 0.003	0.843 ± 0.010	**0.957 ± 0.014**
Mdm2	4ODF	0.943 ± 0.003	0.938 ± 0.006	0.977 ± 0.077	0.932 ± 0.000	0.966 ± 0.000	0.942 ± 0.000	0.952 ± 0.005	**0.999 ± 0.003**
	4ZFI	0.952 ± 0.009	0.937 ± 0.005	0.977 ± 0.059	0.926 ± 0.002	0.962 ± 0.000	0.942 ± 0.000	0.956 ± 0.006	**0.999 ± 0.002**
Menin	5DB2	0.500 ± 0.000	0.500 ± 0.000	0.823 ± 0.129	0.758 ± 0.014	0.903 ± 0.004	0.662 ± 0.002	0.680 ± 0.028	**0.969 ± 0.038**
	6B41	0.538 ± 0.012	0.500 ± 0.000	0.828 ± 0.242	0.760 ± 0.025	0.897 ± 0.008	0.617 ± 0.080	0.679 ± 0.028	**0.971 ± 0.033**

**Table 4:** ROC AUC values for ten PPI targets obtained by applying ML models to the test sets. The best AUC value corresponding to each target protein (across rows) is shown in bold.

Target	PDB ID	Tree	Bagged Forest	Random Forest	Bayesian	SVM	Logistic Regression	Neural Net	Neural Net (Bagging)
BAZ2B	4XUA	0.534	0.5	0.537	0.53	0.521	0.532	0.53	**0.54**
	5E73	0.524	0.5	0.546	0.525	0.54	0.52	0.538	**0.553**
Bcl-2	2O21	0.966	0.949	**0.987**	0.978	0.979	0.972	0.981	0.985
	4LVT	0.948	0.948	**0.992**	0.982	0.976	0.974	0.988	0.991
Bcl-xL	3INQ	0.964	0.91	0.989	0.982	0.983	0.985	**0.99**	0.986
	3WIZ	0.979	0.916	0.989	0.975	0.981	0.986	0.988	**0.993**
BRD4-1	5D3L	**0.926**	**0.926**	0.918	0.897	0.915	0.893	0.917	0.918
	5KU3	0.901	0.901	0.911	0.886	0.908	0.887	0.906	**0.927**
CREBBP	5EIC	0.631	0.5	0.769	0.726	0.724	0.744	0.75	**0.772**
	5MMG	0.689	0.5	0.691	0.712	0.732	0.721	0.746	**0.788**
HIV IN	4CFD	0.822	0.827	0.801	0.74	0.805	0.742	0.784	**0.832**
	4CHO	0.792	0.807	0.791	0.73	0.816	0.714	0.753	**0.824**
XIAP	1TFT	0.97	0.972	0.996	0.979	0.99	0.981	0.994	**0.997**
	5C3H	0.973	0.976	0.994	0.974	0.994	0.985	**0.995**	**0.995**
Mcl-1	5FC4	0.742	0.586	0.854	0.783	0.776	0.813	0.843	**0.859**
	5MES	0.755	0.573	0.845	0.786	0.715	0.809	0.835	**0.846**
Mdm2	4ODF	0.947	0.947	0.958	0.922	0.957	0.946	0.95	**0.963**
	4ZFI	0.941	0.94	0.949	0.919	0.954	0.943	0.951	**0.961**
Menin	5DB2	0.5	0.5	**0.674**	0.622	0.614	0.62	0.646	0.646
	6B41	0.536	0.5	**0.669**	0.601	0.608	0.633	0.656	0.637

## Supplementary Materials

The following supporting information can be downloaded at: https://www.mdpi.com/article/10.3390/ijms232214364/s1.

## Abbreviations

PPIs, protein-protein interactions; VS, virtual screening; SBVS, structure-based virtual screening; PDB, protein data bank; ASP, astex statistical potential; RDS, receptor depth scaling; ML, machine learning; SVM, support vector machine, QSAR, quantitative structure-activity relationship; rSASA, relative solvent accessible surface area; ROC, receiver operating characteristic; AUC, area under the ROC curve; BEDROC, Boltzmann-enhanced discrimination of ROC; EF 1%, enrichment factor at 1%; EF 5%, enrichment factor at 5%; BAZ2B, Bromodomain Adjacent to Zinc Finger Domain 2B; BRD4-1, Bromodomain-containing protein 4; CREBBP, CREB-binding protein; HIV IN, HIV Integrase; XIAP, Inhibitor of apoptosis protein 3; EphA4, Ephrin type-A receptor 4; PCA, principal component analysis; PLIF, protein–ligand interaction fingerprint (PLIF).
