# Supplementary material for: A Hybrid Docking and Machine Learning Approach to Enhance the Performance of Virtual Screening Carried out on Protein–Protein Interfaces
Source: Int J Mol Sci. 2022 Nov 18;23(22):14364. doi: 10.3390/ijms232214364 (PMC9694378; doi:10.3390/ijms232214364)
Supplement: Supplementary file 1 [file ijms-23-14364-s001.zip › ijms-1967945-supplementary.pdf]

# Supporting Information

## A hybrid docking and machine learning approach to enhance the performance of virtual screening carried out on protein-protein interfaces

Natesh Singh, Bruno O. Villoutreix*

*Université de Paris, Inserm UMR 1141 NeuroDiderot, Robert-Debré Hospital, 75019 Paris, France.*

*To whom correspondence should be addressed: bruno.villoutreix@inserm.fr

# Table of Contents


**SUPPORTING FIGURES**

Figure S1 .......... 3-4
Figure S2 .......... 5-6
Figure S3 .......... 7
Figure S4 .......... 8
Figure S5 .......... 9
Figure S6 .......... 10
Figure S7 .......... 11
Figure S8 .......... 12
Figure S9 .......... 13

**SUPPORTING TABLES**

Table S1 .......... 14
Table S2 .......... 15-18
Table S3 .......... 19-21
Table S4 .......... 22-24
Table S5 .......... 25-27
Table S6 .......... 28-30
Table S7 .......... 31-36
Table S8 .......... 37-39
Table S9 .......... 39-43
Table S10 .......... 43-44

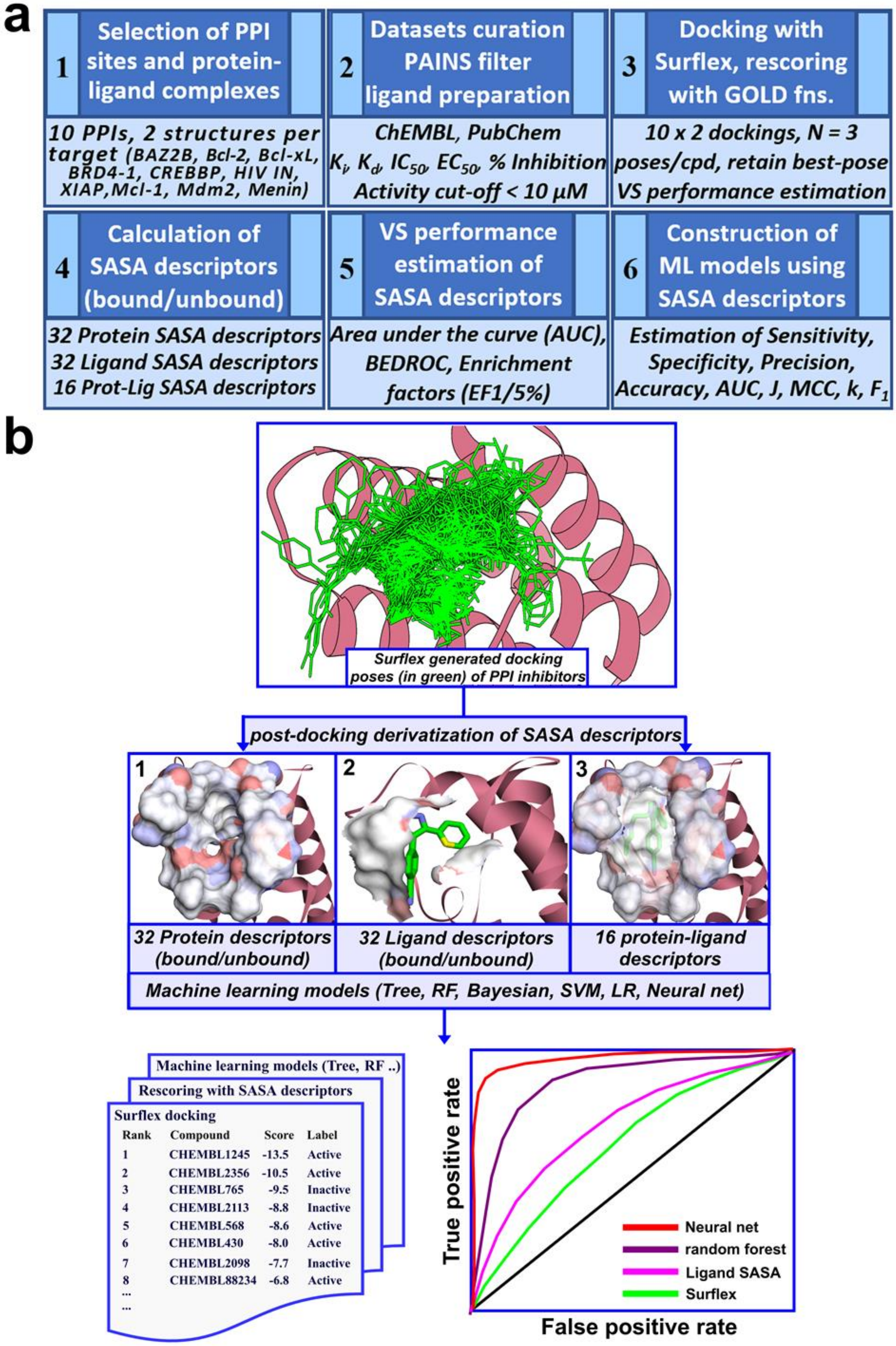
a
1 Selection of PPI sites and protein-ligand complexes
10 PPIs, 2 structures per target (BAZ2B, Bcl-2, Bcl-xL, BRD4-1, CREBBP, HIV IN, XIAP, Mcl-1, Mdm2, Menin)
2 Datasets curation PAINS filter ligand preparation
ChEMBL, PubChem
$K_i$, $K_d$, $IC_{50}$, $EC_{50}$, % Inhibition
Activity cut-off < 10 μM
3 Docking with Surflex, rescoring with GOLD fns.
10 x 2 dockings, N = 3 poses/cpd, retain best-pose
VS performance estimation
4 Calculation of SASA descriptors (bound/unbound)
32 Protein SASA descriptors
32 Ligand SASA descriptors
16 Prot-Lig SASA descriptors
5 VS performance estimation of SASA descriptors
Area under the curve (AUC), BEDROC, Enrichment factors (EF1/5%)
6 Construction of ML models using SASA descriptors
Estimation of Sensitivity, Specificity, Precision, Accuracy, AUC, J, MCC, k, $F_1$
b
Surflex generated docking poses (in green) of PPI inhibitors
post-docking derivatization of SASA descriptors
1
2
3
32 Protein descriptors (bound/unbound)
32 Ligand descriptors (bound/unbound)
16 protein-ligand descriptors
Machine learning models (Tree, RF, Bayesian, SVM, LR, Neural net)
Machine learning models (Tree, RF ..)
Rescoring with SASA descriptors
Surflex docking
Rank Compound Score Label
1 CHEMBL1245 -13.5 Active
2 CHEMBL2356 -10.5 Active
3 CHEMBL765 -9.5 Inactive
4 CHEMBL2113 -8.8 Inactive
5 CHEMBL568 -8.6 Active
6 CHEMBL430 -8.0 Active
7 CHEMBL2098 -7.7 Inactive
8 CHEMBL88234 -6.8 Active
...
...
True positive rate
False positive rate
Neural net
random forest
Ligand SASA
Surflex

**Figure S1.** (**a**) Workflow diagram describing a hybrid computational approach developed in this study that combines structure-based virtual screening, rescoring, and machine learning algorithms: (1) selection of the PPI targets and their protein-ligand structures from the PDB; (2) collecting compounds from ChEMBL and PubChem followed by their categorization into actives or inactives, and then curation and ligand preparation; (3) flexible molecular docking (from the ligand side as two protein structures were used) with the fast ligand docking-scoring protocol of Surflex "pscreen" with several docking poses (e.g., N = 3) generated for each compound. These steps were followed by rescoring of all poses with GOLD scoring functions (GoldScore, ChemScore, ASP, ChemPLP, ChemScore RDS) and through consensus scoring utilizing all scoring functions; (4) post-docking derivatization of SASA descriptors of the protein and the ligand in the bound and unbound states for the top-ranked pose of the compounds obtained from Surflex docking. A set of protein-ligand SASA descriptors were calculated by combining protein and ligand SASA values; (5) VS performance estimation of SASA descriptors; (6) construction of machine learning models (Single tree, Bagged forest, Random forest, Bayesian, SVM, Logistic regression, Neural network, and Neural network with bagging) using SASA descriptors.

(**b**) Schematic representation of the post-processing technique applied to the docking poses and building of the machine learning models. Eighty SASA descriptors were calculated from the top-ranked docking pose of the compounds: (1) 32 protein descriptors; (2) 32 ligand descriptors; and (3) 16 protein-ligand descriptors. These descriptors were used to construct diverse machine learning models. The VS performance of the rescoring methods was assessed by estimating the ROC-based measures such as AUC, log EF1%, EF5%, and BEDROC ($\alpha$ = 20). The performance of the machine learning models was estimated by calculating ROC AUC, sensitivity, specificity, precision, concordance (overall accuracy), J, MCC, $F_1$, and k.

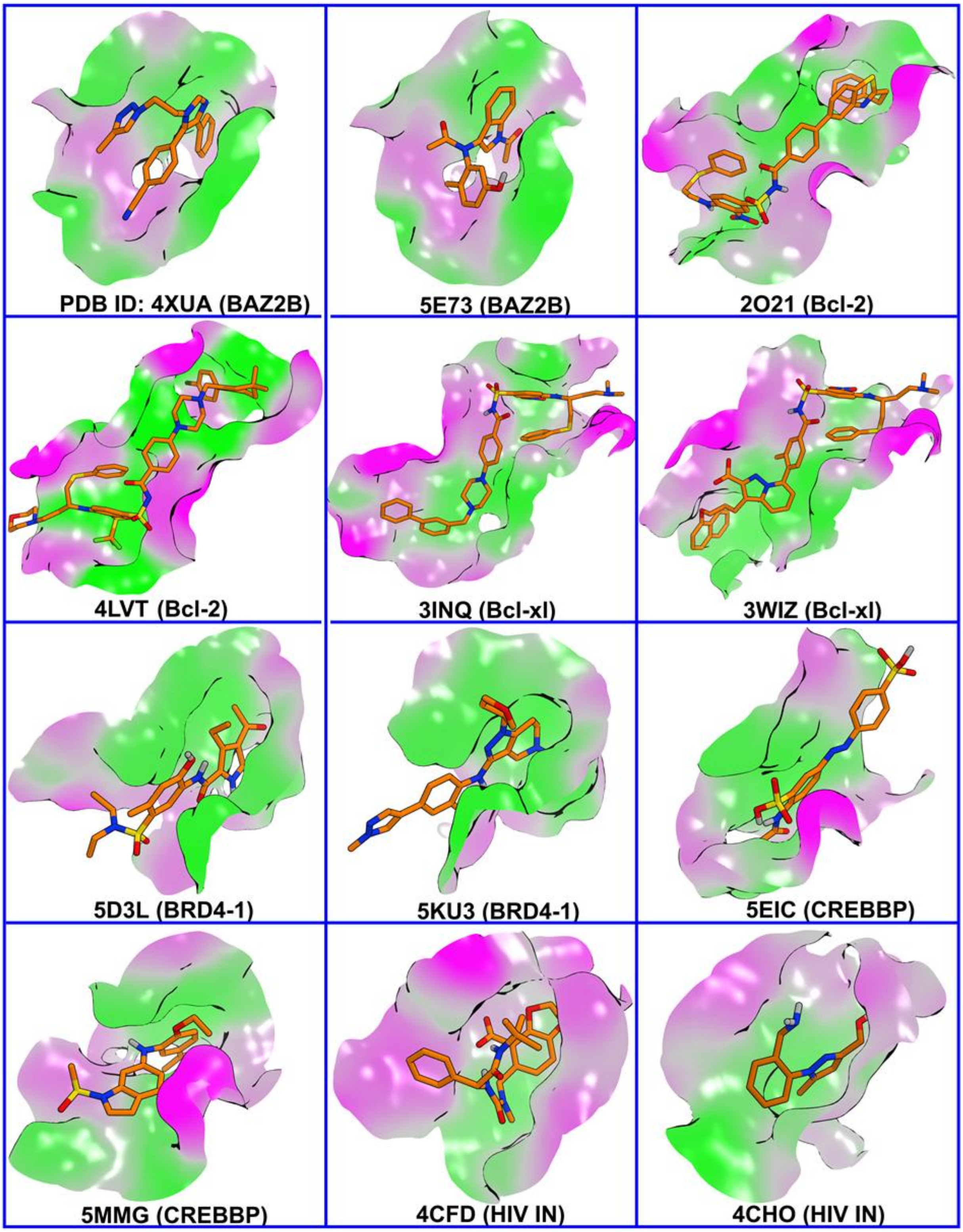
PDB ID: 4XUA (BAZ2B)
5E73 (BAZ2B)
2O21 (Bcl-2)
4LVT (Bcl-2)
3INQ (Bcl-xl)
3WIZ (Bcl-xl)
5D3L (BRD4-1)
5KU3 (BRD4-1)
5EIC (CREBBP)
5MMG (CREBBP)
4CFD (HIV IN)
4CHO (HIV IN)

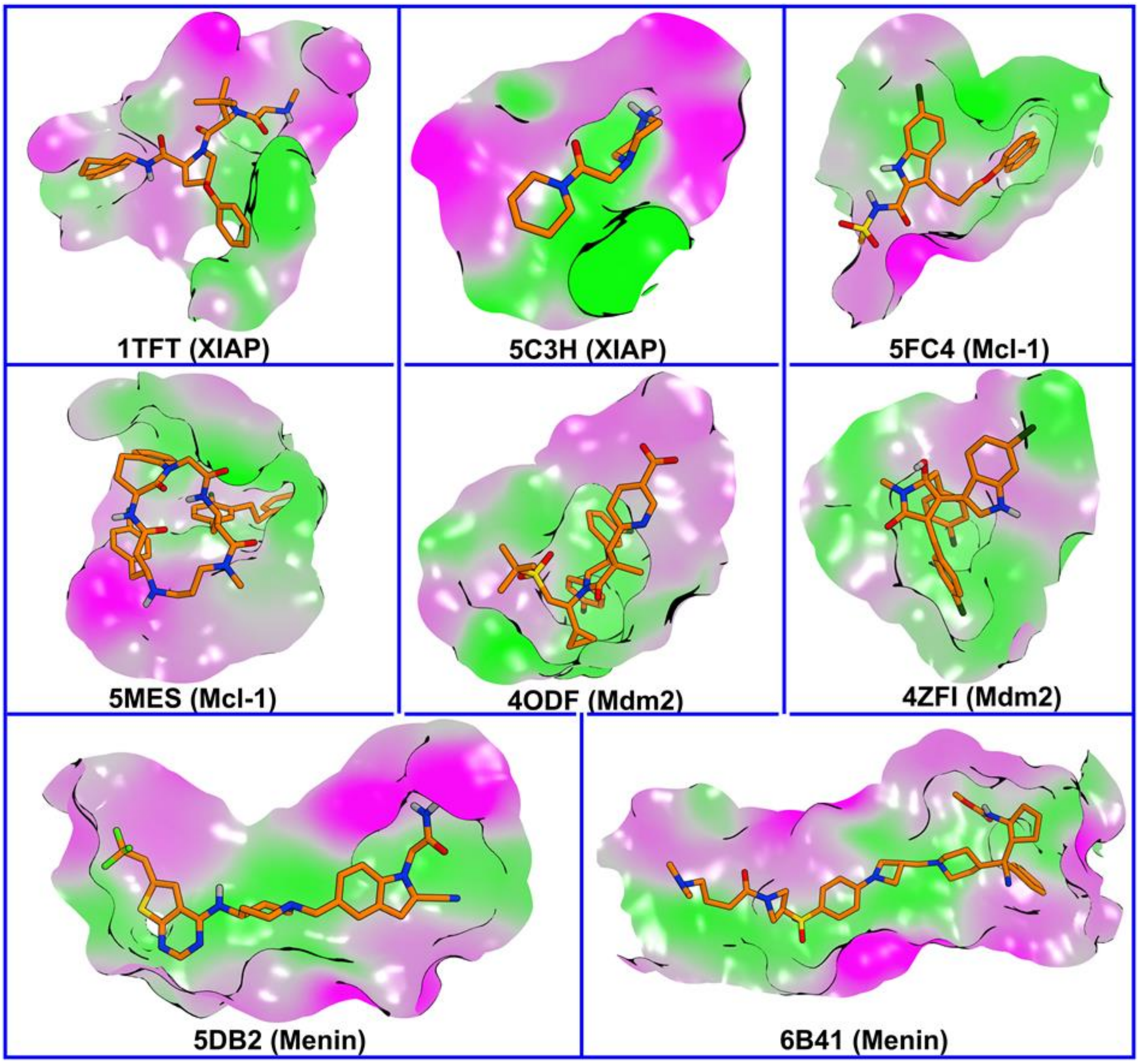


**Figure S2.** The ligand-binding cavities for the cluster protein representatives of the investigated PPI targets: BAZ2B (PDB ID: 4XUA, 5E73), Bcl-2 (2O21, 4LVT), Bcl-xL (3INQ, 3WIZ), BRD4-1 (5D3L, 5KU3), CREBBP (5EIC, 5MMG), HIV IN (4CFD, 4CHO), XIAP (1TFT, 5C3H), Mcl-1 (5FC4, 5MES), Mdm2 (4ODF, 4ZFI), and Menin (5DB2, 6B41). The binding site surface is colored according to the type of residue surrounding the ligand (magenta: hydrophilic residues, green: hydrophobic residues, and grey: neutral areas). The inhibitor bound to the target proteins is shown in stick (orange) representation. The figure was generated using MOE.

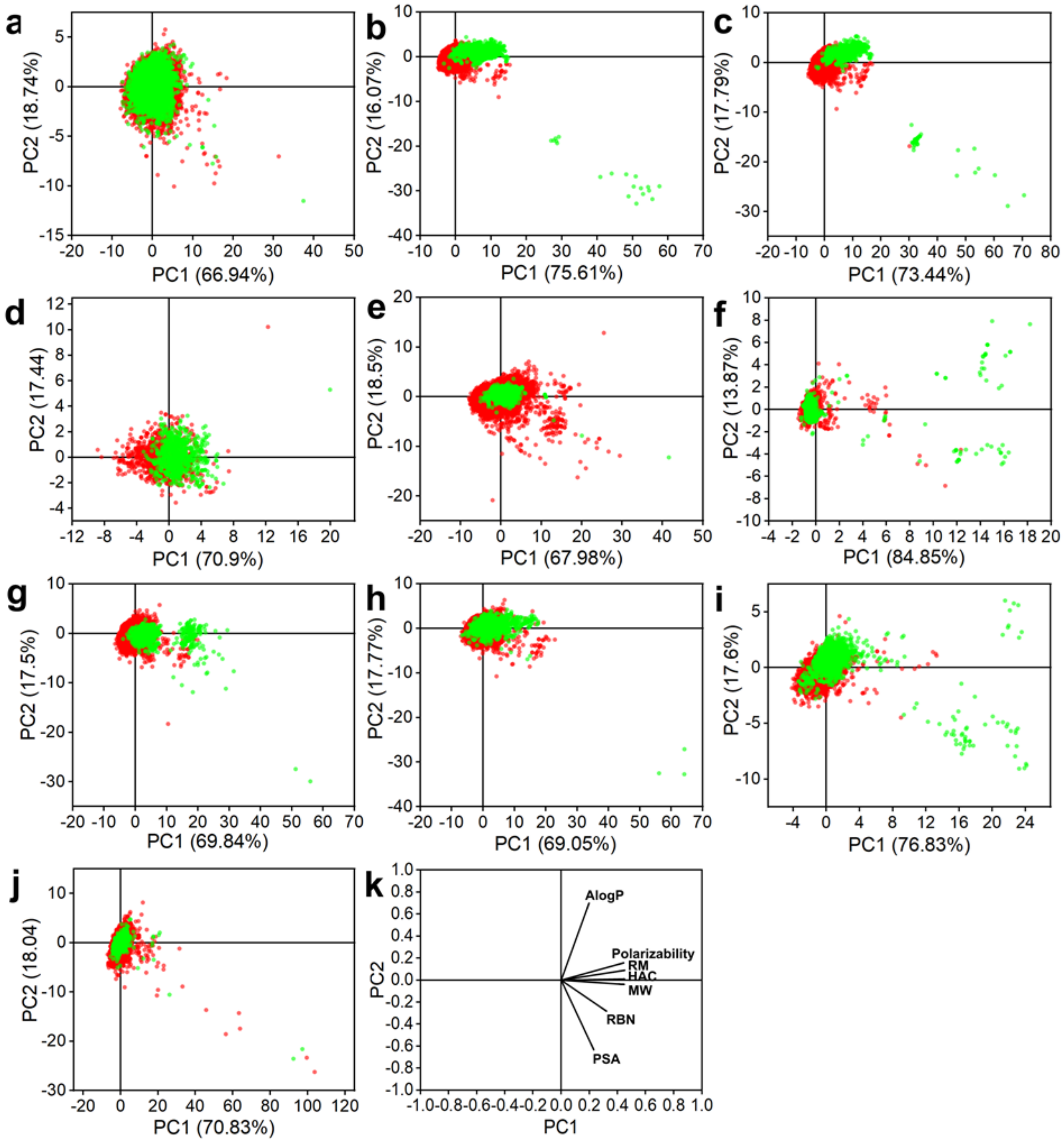


**Figure S3.** PCA score plots of the PPI datasets. Compounds included in the score plots are (**a**) BAZ2B, (**b**) Bcl-2, (**c**) Bcl-xL, (**d**) BRD4-1, (**e**) CREBBP, (**f**) HIV IN, (**g**) XIAP, (**h**) Mcl-1, (**i**) Mdm2, and (**j**) Menin. Figure (**k**) shows the loading plot of descriptors used for PCA analysis. The compounds were described by seven simple descriptors: Number of heavy atoms (HAC), Molecular weight (MW), Number of rotatable bonds (RBN), AlogP, Polar surface area (PSA), Molar refractivity (RM), and Polarizability. Active and inactive compounds are shown in green and red circles, respectively. The first principal component describes in part the size (HAC and MW), and the second gives insights about polarity (PSA) and lipophilicity (AlogP).

**Figure S4.** Comparison of AUC values (y-axis) attained for ten PPI targets (twenty protein structures) using Surflex and different docking-pose derived SASA descriptors (x-axis). Each boxplot shows twenty AUC values for a given scoring method. A marked increase in mean and scoring performance (measured as AUC) can be seen for several SASA scoring metrics compared to Surflex.

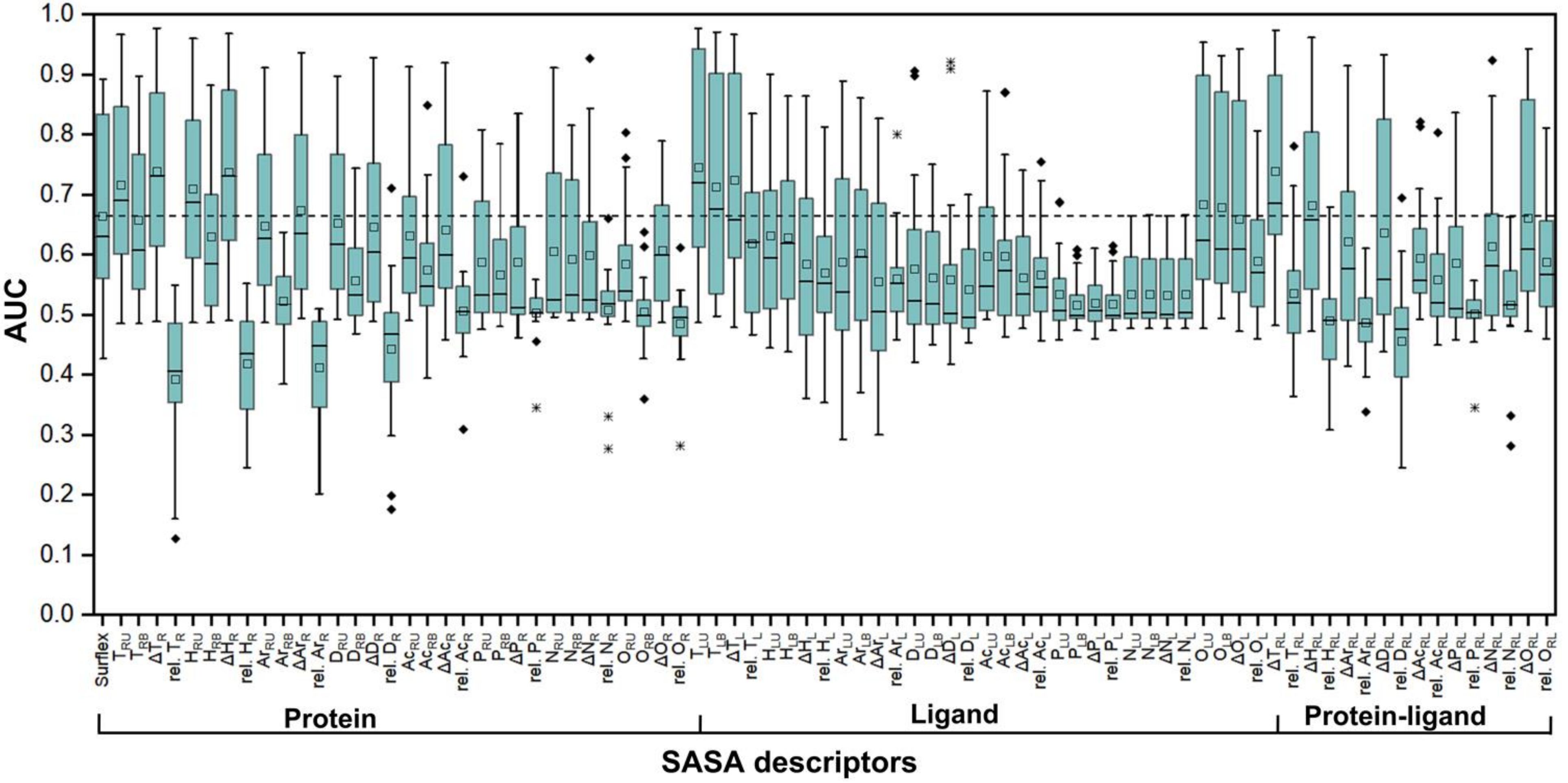

**Figure S5.** Comparison of EF 1% (**a**) and EF 5% (**b**) (y-axis) results attained for ten PPI targets (twenty protein structures) using Surflex and different SASA scoring methods (x-axis). Each boxplot shows twenty EF values for a given scoring method. A marked increase in mean and scoring performance (measured as EF1% and EF5%) can be seen in scoring methods SASA receptor total unbound ($T_{RU}$), SASA receptor total delta ($\Delta T_R$), SASA receptor hydrophobic delta ($\Delta H_R$), SASA ligand total unbound ($T_{LU}$), SASA ligand total bound ($T_{LB}$), SASA ligand total delta ($\Delta T_L$), SASA ligand other unbound ($O_{LU}$), SASA receptor-ligand total delta ($\Delta T_{RL}$) as compared to the Surflex scoring function.

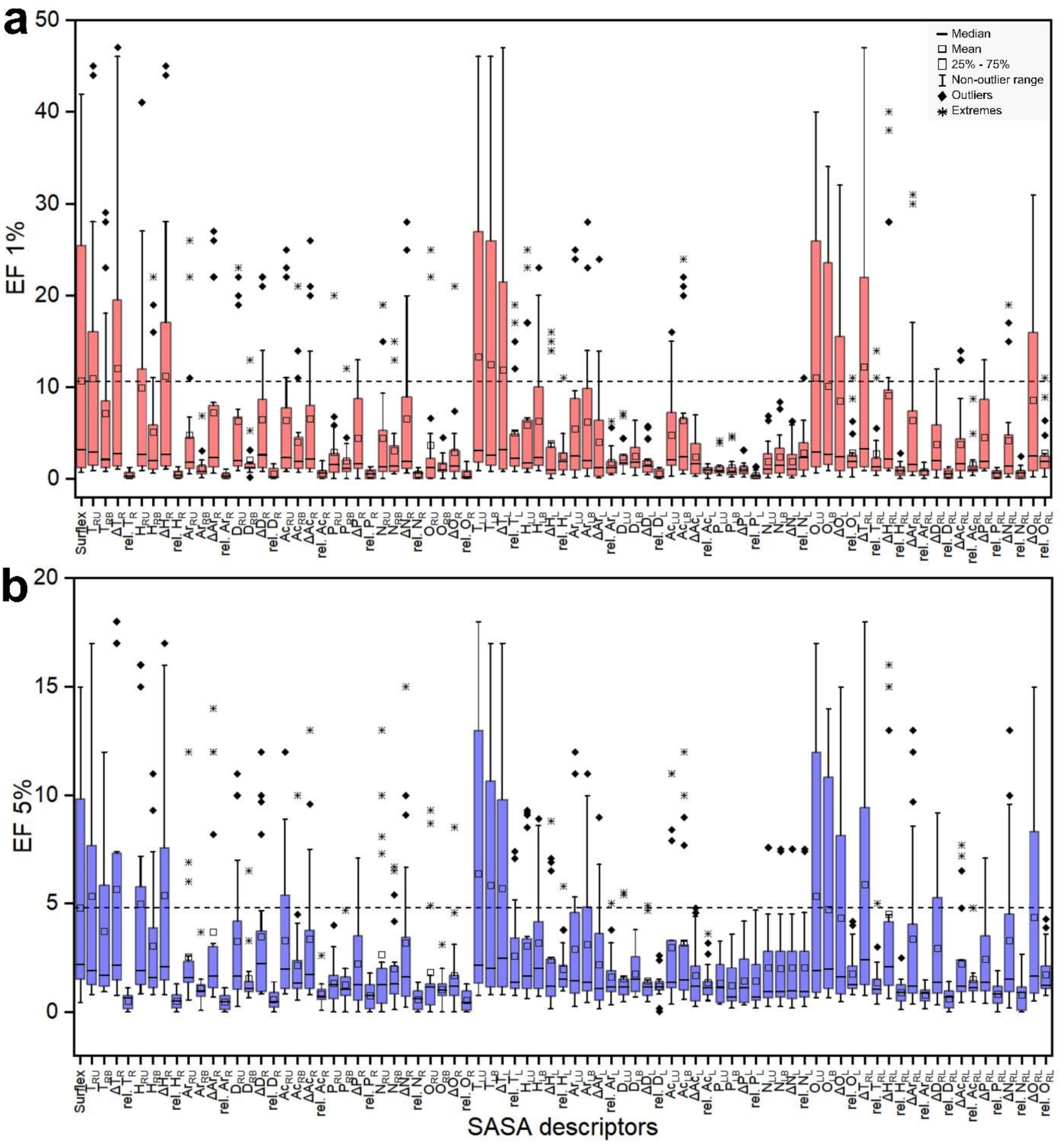

**Figure S6.** Heat map plot showing the BEDROC values for the ten PPI targets and their protein structures (y-axis) obtained using 32 protein SASA descriptors, 32 ligand SASA descriptors, and 16 protein-ligand SASA descriptors. The calculations were performed for each target-specific set of actives and inactives.

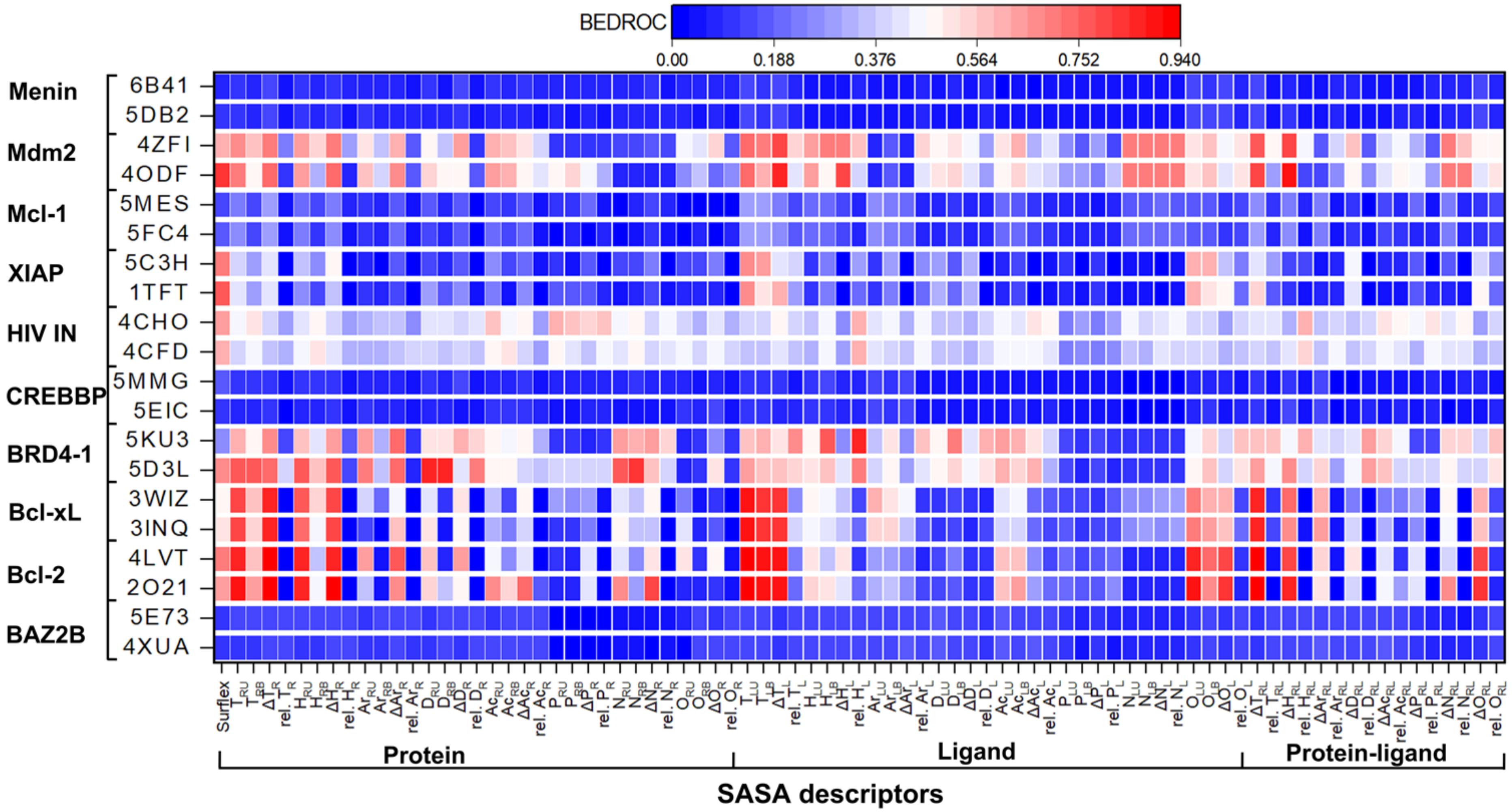

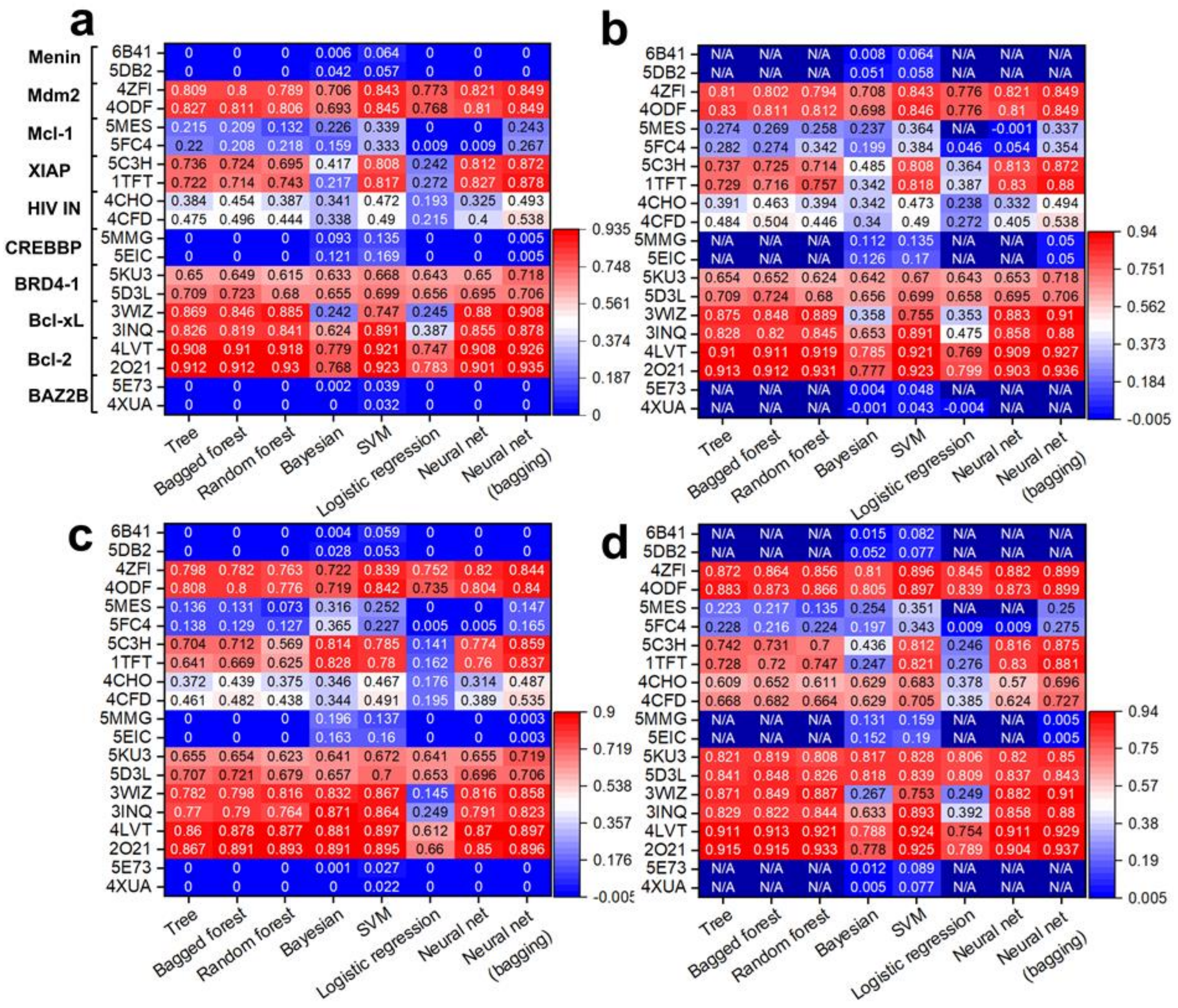


**Figure S7.** The heat maps show the additional external statistics for the machine learning models: (**a**) κ (**b**) MCC (**c**) J (**d**) F1.

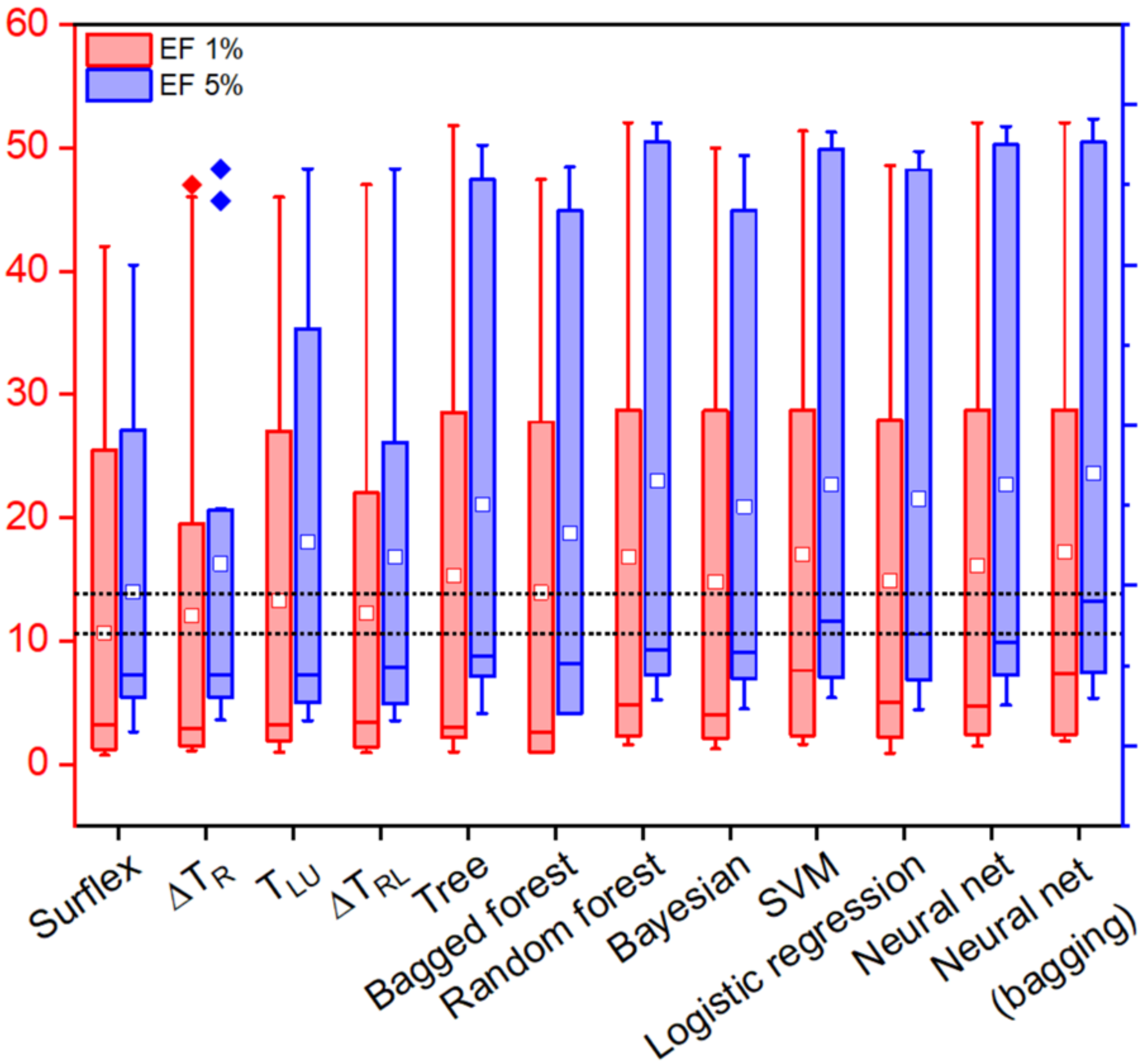


**Figure S8.** Comparison of EF1% and EF5% (y-axis) results attained for ten PPI targets (twenty protein structures) using Surflex; the best performing receptor SASA descriptor $\Delta T_R$; the best performing ligand SASA descriptor $T_{LU}$; the best performing receptor-ligand SASA descriptor $\Delta T_{RL}$; and different machine learning methods (x-axis): Each boxplot shows twenty EF values for a given method. The horizontal black dotted lines in the plot indicate a marked increase in mean and scoring performance (measured as EF 1% and EF 5%) of the 3D descriptors and ML models compared to Surflex. Among the different ML methods, neural net (bagging) produced the best results on this PPI dataset.

**Figure S9.** Relative molecular descriptor importance inferred from the Neural network (bagging) models. Each box plot underlines the relative importance of the descriptors inferred from the 20 Neural networks (bagging) classifier models trained to discriminate between active and inactive PPI molecules. The importance of the descriptors is sorted from highest (SASA receptor hydrophobic delta) to lowest (relative SASA receptor other). The x-axis reports the SASA descriptors, and the y-axis the relative importance of the descriptors.

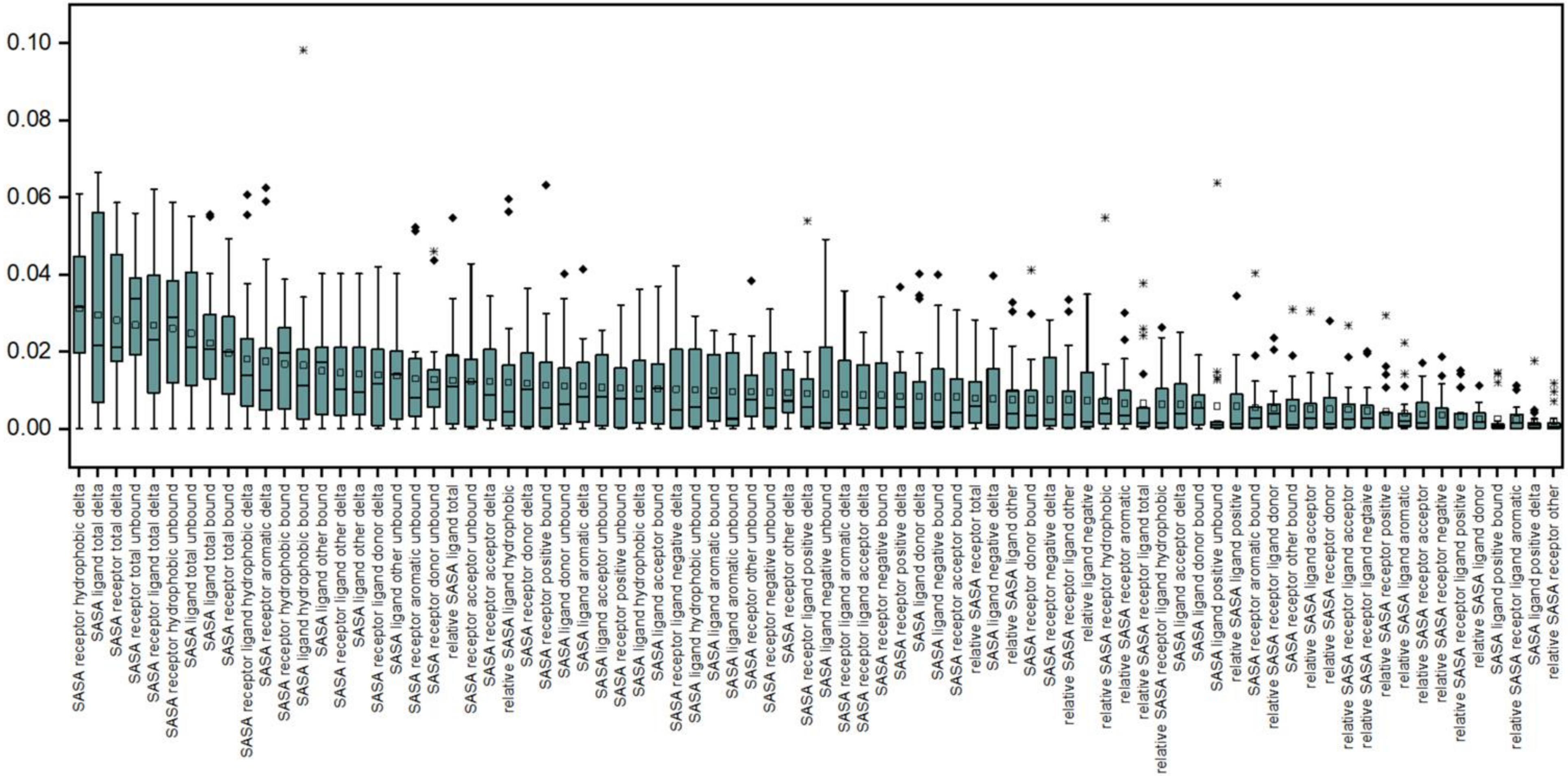

**Table S1**. The Enrichment Factors (EF 1% and EF 5%) for the protein structures of ten PPI targets obtained after Surflex docking, re-scoring with GOLD scoring functions, and the consensus scoring method. The best enrichment values corresponding to each target protein (across rows) for EF 1% and EF 5% are shown in bold.

| Target | PDB ID | Enrichment factors | **Surflex** | **GoldScore** | **ChemScore** | **ASP** | **ChemPLP** | **ChemScore RDS** | **Consensus scoring** |
|---|---|---|---|---|---|---|---|---|---|
| BAZ2B | 4XUA | EF 1% | 0.77 | 0.77 | 1.2 | **1.4** | 0.92 | 1 | 1 |
| | | EF 5% | 0.88 | 0.94 | **1.1** | 1 | 0.95 | 0.98 | 1 |
| | 5E73 | EF 1% | 0.86 | 0.7 | **1.3** | 1 | 1 | 1.1 | 0.93 |
| | | EF 5% | 0.89 | 0.84 | **1** | 0.98 | 0.93 | **1** | 0.96 |
| Bcl-2 | 2O21 | EF 1% | 25 | 26 | 23 | 22 | **28** | 6.6 | 21 |
| | | EF 5% | 11 | 11 | 10 | 11 | **13** | 3.4 | 9.8 |
| | 4LVT | EF 1% | **28** | 27 | **28** | **28** | **28** | 16 | **28** |
| | | EF 5% | 12 | 12 | 12 | 12 | **13** | 6.2 | **13** |
| Bcl-xL | 3INQ | EF 1% | 26 | 25 | 19 | 20 | **27** | 7.3 | 21 |
| | | EF 5% | **10** | 8.8 | 8 | 7.8 | 9.9 | 3.6 | 8.6 |
| | 3WIZ | EF 1% | 27 | 30 | 27 | 21 | **33** | 6.5 | 30 |
| | | EF 5% | 9.7 | 9.9 | 10 | 9.1 | **12** | 3.3 | 11 |
| BRD4-1 | 5D3L | EF 1% | 0.96 | 1.4 | 1.9 | 1.9 | 1.9 | **2.2** | 1.9 |
| | | EF 5% | 1.4 | 1.7 | **1.9** | 1.7 | **1.9** | **1.9** | 1.8 |
| | 5KU3 | EF 1% | 0.72 | 1.3 | 0.6 | **1.8** | 0.84 | 0.48 | 1.3 |
| | | EF 5% | 0.43 | **1.4** | 1.2 | 1.3 | 1.1 | 1.1 | **1.4** |
| CREBBP | | EF 1% | 1.8 | 1.6 | 2.8 | 1.8 | 2.8 | 1.4 | **2.9** |
| | 5EIC | EF 5% | 1.6 | 1.9 | 2.1 | **2.7** | 2.2 | 1.3 | 2.6 |
| | | EF 1% | **5.1** | 2.3 | 2.7 | 2.5 | 2.4 | 1.1 | 2.9 |
| | 5MMG | EF 5% | **2.7** | 2.1 | 1.9 | 2.4 | 1.9 | 1.3 | 2 |
| HIV IN | 4CFD | EF 1% | **1.4** | 0.35 | 0.7 | 1.2 | 0.7 | 0.35 | **1.4** |
| | | EF 5% | **1.6** | 0.8 | 0.94 | 1.4 | 1.1 | 0.82 | 1.3 |
| | 4CHO | EF 1% | 1.5 | 0.46 | 0.81 | **1.7** | 1.2 | 0.7 | 1.3 |
| | | EF 5% | **1.6** | 0.96 | 0.91 | 1.4 | 1.3 | 0.77 | 1.3 |
| XIAP | 1TFT | EF 1% | **42** | 31 | 31 | 20 | 30 | 31 | 29 |
| | | EF 5% | **15** | 12 | 11 | 9.8 | 11 | 10 | 11 |
| | 5C3H | EF 1% | 33 | 9.3 | 23 | 24 | 33 | 29 | **35** |
| | | EF 5% | **14** | 6.8 | 9.7 | 9.5 | 12 | 11 | 12 |
| Mcl-1 | 5FC4 | EF 1% | 6.1 | 8.3 | 11 | **12** | 11 | 9.4 | 10 |
| | | EF 5% | 2.8 | 3.9 | **4.4** | 3.6 | 4.2 | 4 | 4.1 |
| | 5MES | EF 1% | 3.3 | **7.4** | 5.5 | 3.6 | 5.4 | 3.9 | 5.8 |
| | | EF 5% | 2.5 | **3.5** | 2.7 | 2.5 | 2.6 | 2.2 | 2.8 |
| Mdm2 | 4ODF | EF 1% | **3** | **3** | 2.8 | 2.9 | 2.8 | 2.8 | 2.8 |
| | | EF 5% | **2.9** | **2.9** | 2.7 | 2.7 | 2.7 | 2.2 | 2.8 |
| | 4ZFI | EF 1% | 2.5 | **2.8** | 1.4 | 2 | 1.3 | 0.67 | 1.7 |
| | | EF 5% | 1.9 | **2.4** | 1.4 | 1.6 | 1.2 | 0.7 | 1.5 |
| Menin | 5DB2 | EF 1% | 3.4 | **6** | 2 | 5.3 | 5.3 | 1.6 | **6** |
| | | EF 5% | 1.9 | **2.4** | 1.9 | 2.2 | 2.1 | 1.7 | 2.3 |
| | 6B41 | EF 1% | 1 | **3.3** | 1.7 | 3 | 3.1 | 1.1 | 2.4 |
| | | EF 5% | 1.2 | **1.9** | 1.1 | 1.7 | 1.6 | 0.94 | 1.2 |

**Table S2.** The AUC values for the PPI datasets screened using **32 protein SASA descriptors**: SASA receptor total unbound($T_{RU}$), SASA receptor total bound ($T_{RB}$), SASA receptor total delta ($\Delta T_R$), relative SASA receptor total (rel. $T_R$) , SASA receptor hydrophobic unbound ($H_{RU}$), SASA receptor hydrophobic bound ($H_{RB}$), SASA receptor hydrophobic delta ($\Delta H_R$), relative SASA receptor hydrophobic (rel. $H_R$), SASA receptor aromatic unbound ($Ar_{RU}$), SASA receptor aromatic bound ($Ar_{RB}$), SASA receptor aromatic delta ($\Delta Ar_R$), relative SASA receptor aromatic (rel. $Ar_R$), SASA receptor donor unbound ($D_{RU}$), SASA receptor donor bound ($D_{RB}$), SASA receptor donor delta ($\Delta D_R$), relative SASA receptor donor (rel. $D_R$), SASA receptor acceptor unbound ($Ac_{RU}$), SASA receptor acceptor bound ($Ac_{RB}$), SASA receptor acceptor delta ($\Delta Ac_R$), relative SASA receptor acceptor (rel. $Ac_R$), SASA receptor positive unbound ($P_{RU}$), SASA receptor positive bound ($P_{RB}$), SASA receptor positive delta ($\Delta P_R$), relative SASA receptor positive (rel. $P_R$), SASA receptor negative unbound ($N_{RU}$), SASA receptor negative bound ($N_{RB}$), SASA receptor negative delta ($\Delta N_R$), relative SASA receptor negative (rel. $N_R$), SASA receptor other unbound ($O_{RU}$), SASA receptor other bound ($O_{RB}$), SASA receptor other delta ($\Delta O_R$), relative SASA receptor other (rel. $O_R$); **32 ligand SASA descriptors**: SASA ligand total unbound ($T_{LU}$), SASA ligand total bound ($T_{LB}$), SASA ligand total delta ($\Delta T_L$), relative SASA ligand total (rel. $T_L$), SASA ligand hydrophobic unbound ($H_{LU}$), SASA ligand hydrophobic bound ($H_{LB}$), SASA ligand hydrophobic delta ($\Delta H_L$), relative SASA ligand hydrophobic (rel. $H_L$), SASA ligand aromatic unbound ($Ar_{LU}$), SASA ligand aromatic bound ($Ar_{LB}$), SASA ligand aromatic delta ($\Delta Ar_L$), relative SASA ligand aromatic (rel. $Ar_L$), SASA ligand donor unbound ($D_{LU}$), SASA ligand donor bound ($D_{LB}$), SASA ligand donor delta ($\Delta D_L$), relative SASA ligand donor (rel. $D_L$), SASA ligand acceptor unbound ($Ac_{LU}$), SASA ligand acceptor bound ($Ac_{LB}$), SASA ligand acceptor delta ($\Delta Ac_L$), relative SASA ligand acceptor (rel. $Ac_L$), SASA ligand positive unbound ($P_{LU}$), SASA ligand positive bound ($P_{LB}$), SASA ligand positive delta ($\Delta P_L$), relative SASA ligand positive (rel. $P_L$), SASA negative unbound ($N_{LU}$), SASA ligand negative bound ($N_{LB}$), SASA ligand negative delta ($\Delta N_L$), relative SASA ligand negative (rel. $N_L$), SASA ligand other unbound ($O_{LU}$), SASA ligand other bound ($O_{LB}$), SASA ligand other delta ($\Delta O_L$), relative SASA ligand other (rel. $O_L$); and **16 protein-ligand SASA descriptors**: SASA receptor ligand total delta ($\Delta T_{RL}$), relative SASA receptor ligand total (rel. $T_{RL}$), SASA receptor ligand hydrophobic delta ($\Delta H_{RL}$), relative SASA receptor ligand hydrophobic (rel. $H_{RL}$), SASA receptor ligand aromatic delta ($\Delta Ar_{RL}$), relative SASA receptor ligand aromatic (rel. $Ar_{RL}$), SASA receptor ligand donor delta ($\Delta D_{RL}$), relative SASA receptor ligand donor (rel. $D_{RL}$), SASA receptor ligand acceptor delta ($\Delta Ac_{RL}$),

relative SASA receptor ligand acceptor (rel. $Ac_{RL}$), SASA receptor ligand positive delta ($\Delta P_{RL}$), relative SASA receptor ligand positive (rel. $P_{RL}$), SASA receptor ligand negative delta ($\Delta N_{RL}$), relative SASA receptor ligand negative (rel. $N_{RL}$), SASA receptor ligand other delta ($\Delta O_{RL}$), relative SASA receptor ligand other (rel. $O_{RL}$). The best AUC score corresponding to each target protein (across columns) is indicated in bold and highlighted in green.

| SASA descriptors | BAZ2B | | BCL2 | | BCLXL | | BRD4-1 | | CREBBP | | HIV-IN | | XIAP | | MCL1 | | MDM2 | | Menin | |
|---|---|---|---|---|---|---|---|---|---|---|---|---|---|---|---|---|---|---|---|---|
| | 4XUA | 5E73 | 2O21 | 4LVT | 3INQ | 3WIZ | 5D3L | 5KU3 | 5EIC | 5MMG | 4CFD | 4CHO | 1TFT | 5C3H | 5FC4 | 5MES | 4ODF | 4ZFI | 5DB2 | 6B41 |
| Surflex | 0.491 | 0.482 | 0.857 | 0.886 | 0.844 | 0.823 | 0.696 | 0.426 | 0.604 | **0.662** | 0.627 | 0.631 | 0.888 | 0.892 | 0.585 | 0.59 | 0.64 | 0.57 | 0.548 | 0.517 |
| $T_{RU}$ | 0.485 | 0.485 | 0.965 | 0.966 | 0.944 | 0.950 | 0.810 | 0.738 | 0.594 | 0.639 | 0.605 | 0.613 | 0.857 | 0.835 | 0.647 | 0.625 | 0.733 | 0.746 | 0.535 | 0.531 |
| $T_{RB}$ | 0.486 | 0.484 | 0.898 | 0.896 | 0.868 | 0.892 | 0.742 | 0.606 | 0.520 | 0.608 | 0.582 | 0.590 | 0.777 | 0.758 | 0.562 | 0.567 | 0.621 | 0.664 | 0.519 | 0.502 |
| $DT_R$ | 0.488 | 0.489 | 0.976 | 0.977 | 0.960 | 0.963 | 0.805 | 0.789 | 0.662 | 0.632 | 0.606 | 0.619 | 0.872 | 0.869 | 0.675 | 0.670 | 0.830 | 0.805 | 0.544 | 0.542 |
| rel. $T_R$ | 0.491 | 0.487 | 0.159 | 0.127 | 0.167 | 0.311 | 0.550 | 0.393 | 0.392 | 0.503 | 0.475 | 0.497 | 0.360 | 0.348 | 0.364 | 0.418 | 0.396 | 0.458 | 0.486 | 0.467 |
| $H_{RU}$ | 0.487 | 0.486 | 0.955 | 0.959 | 0.938 | 0.941 | 0.790 | 0.718 | 0.580 | 0.628 | 0.642 | 0.634 | 0.804 | 0.843 | 0.654 | 0.608 | 0.723 | 0.744 | 0.528 | 0.533 |
| $H_{RB}$ | 0.487 | 0.486 | 0.858 | 0.818 | 0.862 | 0.881 | 0.648 | 0.579 | 0.487 | 0.590 | 0.570 | 0.596 | 0.667 | 0.731 | 0.563 | 0.563 | 0.509 | 0.666 | 0.506 | 0.518 |
| $DH_R$ | 0.489 | 0.491 | 0.968 | 0.962 | 0.945 | 0.946 | **0.821** | 0.781 | 0.665 | 0.628 | **0.661** | 0.626 | 0.851 | 0.896 | 0.679 | 0.618 | 0.832 | 0.793 | 0.542 | 0.538 |
| rel. $H_R$ | 0.490 | 0.487 | 0.306 | 0.343 | 0.340 | 0.307 | 0.432 | 0.435 | 0.386 | 0.509 | 0.457 | 0.509 | 0.246 | 0.352 | 0.395 | 0.486 | 0.344 | 0.551 | 0.480 | 0.500 |
| $Ar_{RU}$ | 0.487 | 0.494 | 0.824 | 0.911 | 0.772 | 0.821 | 0.804 | 0.760 | 0.604 | 0.559 | 0.555 | 0.544 | 0.646 | 0.667 | 0.570 | 0.550 | 0.649 | 0.672 | 0.547 | 0.510 |
| $Ar_{RB}$ | 0.486 | 0.490 | 0.463 | 0.638 | 0.461 | 0.594 | 0.599 | 0.490 | 0.530 | 0.542 | 0.539 | 0.545 | 0.582 | 0.384 | 0.477 | 0.512 | 0.521 | 0.593 | 0.481 | 0.514 |
| $DAr_R$ | 0.495 | 0.502 | 0.927 | 0.936 | 0.896 | 0.868 | 0.801 | **0.799** | 0.545 | 0.542 | 0.551 | 0.548 | 0.656 | 0.681 | 0.617 | 0.587 | 0.781 | 0.704 | 0.527 | 0.496 |
| rel. $Ar_R$ | 0.490 | 0.487 | 0.202 | 0.318 | 0.208 | 0.375 | 0.353 | 0.283 | 0.502 | 0.503 | 0.508 | 0.475 | 0.409 | 0.336 | 0.434 | 0.437 | 0.459 | 0.463 | 0.479 | 0.509 |
| $D_{RU}$ | 0.496 | 0.493 | 0.845 | 0.897 | 0.838 | 0.820 | 0.758 | 0.621 | 0.515 | 0.635 | 0.553 | 0.516 | 0.746 | 0.777 | 0.570 | 0.608 | 0.667 | 0.616 | 0.548 | 0.537 |
| $D_{RB}$ | 0.500 | 0.499 | 0.628 | 0.580 | 0.501 | 0.617 | 0.743 | 0.606 | 0.498 | 0.528 | 0.539 | 0.504 | 0.659 | 0.584 | 0.495 | 0.546 | 0.655 | 0.469 | 0.507 | 0.469 |
| $DD_R$ | 0.489 | 0.490 | 0.881 | 0.928 | 0.872 | 0.874 | 0.488 | 0.519 | 0.522 | 0.649 | 0.518 | 0.527 | 0.758 | 0.708 | 0.584 | 0.624 | 0.623 | 0.745 | 0.551 | 0.555 |
| rel. $D_R$ | 0.503 | 0.500 | 0.298 | 0.198 | 0.175 | 0.332 | 0.711 | 0.582 | 0.486 | 0.431 | 0.515 | 0.503 | 0.535 | 0.454 | 0.436 | 0.417 | 0.496 | 0.358 | 0.479 | 0.449 |
| $Ac_{RU}$ | 0.497 | 0.494 | 0.912 | 0.833 | 0.774 | 0.832 | 0.489 | 0.498 | 0.626 | 0.584 | 0.548 | 0.586 | 0.704 | 0.689 | 0.601 | 0.570 | 0.670 | 0.633 | 0.521 | 0.552 |
| $Ac_{RB}$ | 0.501 | 0.500 | 0.849 | 0.734 | 0.732 | 0.651 | 0.519 | 0.462 | 0.598 | 0.559 | 0.519 | 0.534 | 0.393 | 0.565 | 0.566 | 0.508 | 0.631 | 0.608 | 0.519 | 0.535 |
| $DAc_R$ | 0.494 | 0.490 | 0.918 | 0.826 | 0.794 | 0.821 | 0.459 | 0.553 | 0.634 | 0.592 | 0.550 | 0.595 | 0.856 | 0.771 | 0.599 | 0.604 | 0.602 | 0.612 | 0.510 | 0.540 |
| rel. $Ac_R$ | 0.504 | 0.504 | 0.729 | 0.488 | 0.549 | 0.469 | 0.556 | 0.430 | 0.542 | 0.509 | 0.460 | 0.467 | 0.309 | 0.483 | 0.484 | 0.459 | 0.571 | 0.563 | 0.512 | 0.514 |

| | | | | | | | | | | | | | | | | | | | | |
|---|---|---|---|---|---|---|---|---|---|---|---|---|---|---|---|---|---|---|---|---|
| $P_{RU}$ | 0.500 | 0.500 | 0.735 | 0.776 | 0.699 | 0.808 | 0.502 | 0.501 | 0.474 | 0.531 | 0.535 | 0.534 | 0.767 | 0.679 | 0.502 | 0.599 | 0.593 | 0.503 | 0.506 | 0.508 |
| $P_{RB}$ | 0.500 | 0.500 | 0.629 | 0.642 | 0.619 | 0.761 | 0.502 | 0.501 | 0.480 | 0.532 | 0.544 | 0.534 | 0.785 | 0.644 | 0.515 | 0.538 | 0.588 | 0.503 | 0.502 | 0.509 |
| $DP_R$ | 0.500 | 0.500 | 0.831 | 0.834 | 0.731 | 0.803 | 0.502 | 0.501 | 0.462 | 0.507 | 0.518 | 0.524 | 0.648 | 0.631 | 0.508 | 0.646 | 0.588 | 0.504 | 0.506 | 0.500 |
| rel. $P_R$ | 0.500 | 0.500 | 0.492 | 0.345 | 0.503 | 0.529 | 0.501 | 0.501 | 0.522 | 0.524 | 0.542 | 0.533 | 0.537 | 0.455 | 0.513 | 0.488 | 0.558 | 0.503 | 0.503 | 0.509 |
| $N_{RU}$ | 0.497 | 0.496 | 0.911 | 0.810 | 0.833 | 0.796 | 0.728 | 0.587 | 0.500 | 0.521 | 0.524 | 0.526 | 0.524 | 0.740 | 0.527 | 0.561 | 0.505 | 0.502 | 0.515 | 0.498 |
| $N_{RB}$ | 0.497 | 0.497 | 0.814 | 0.759 | 0.775 | 0.765 | 0.726 | 0.583 | 0.500 | 0.521 | 0.539 | 0.541 | 0.503 | 0.720 | 0.520 | 0.541 | 0.505 | 0.502 | 0.524 | 0.490 |
| $DN_R$ | 0.499 | 0.498 | 0.927 | 0.844 | 0.797 | 0.814 | 0.623 | 0.582 | 0.497 | 0.515 | 0.492 | 0.504 | 0.541 | 0.689 | 0.532 | 0.584 | 0.505 | 0.502 | 0.505 | 0.509 |
| rel. $N_R$ | 0.497 | 0.497 | 0.331 | 0.277 | 0.518 | 0.521 | 0.660 | 0.576 | 0.500 | 0.520 | 0.541 | 0.559 | 0.497 | 0.567 | 0.520 | 0.540 | 0.505 | 0.501 | 0.519 | 0.485 |
| $O_{RU}$ | 0.489 | 0.490 | 0.738 | 0.804 | 0.762 | 0.746 | 0.537 | 0.540 | 0.563 | 0.549 | 0.533 | 0.531 | 0.529 | 0.609 | 0.533 | 0.500 | 0.589 | 0.624 | 0.514 | 0.502 |
| $O_{RB}$ | 0.493 | 0.494 | 0.614 | 0.556 | 0.637 | 0.498 | 0.426 | 0.465 | 0.496 | 0.522 | 0.501 | 0.475 | 0.360 | 0.560 | 0.522 | 0.500 | 0.472 | 0.525 | 0.485 | 0.492 |
| $DO_R$ | 0.487 | 0.489 | 0.732 | 0.789 | 0.734 | 0.705 | 0.639 | 0.583 | 0.614 | 0.550 | 0.535 | 0.559 | 0.735 | 0.648 | 0.521 | 0.500 | 0.623 | 0.658 | 0.524 | 0.510 |
| rel. $O_R$ | 0.498 | 0.497 | 0.611 | 0.539 | 0.515 | 0.461 | 0.426 | 0.465 | 0.461 | 0.510 | 0.501 | 0.475 | 0.282 | 0.526 | 0.522 | 0.500 | 0.469 | 0.453 | 0.485 | 0.491 |
| $T_{LU}$ | 0.487 | 0.486 | **0.977** | **0.978** | **0.970** | **0.968** | 0.752 | 0.744 | 0.638 | 0.641 | 0.607 | 0.616 | **0.942** | **0.944** | 0.691 | 0.697 | 0.840 | **0.844** | 0.547 | 0.543 |
| $T_{LB}$ | 0.498 | 0.499 | 0.970 | 0.969 | 0.961 | 0.952 | 0.710 | 0.777 | 0.622 | 0.597 | 0.537 | 0.532 | 0.880 | 0.926 | 0.670 | 0.682 | 0.646 | 0.759 | 0.521 | 0.530 |
| $DT_L$ | 0.480 | 0.478 | 0.964 | 0.966 | 0.950 | 0.958 | 0.711 | 0.580 | 0.605 | 0.616 | 0.638 | **0.651** | 0.927 | 0.873 | 0.661 | 0.653 | 0.842 | 0.809 | 0.551 | 0.543 |
| rel. $T_L$ | 0.505 | 0.508 | 0.686 | 0.721 | 0.833 | 0.734 | 0.667 | 0.762 | 0.588 | 0.544 | 0.499 | 0.468 | 0.688 | 0.833 | 0.619 | 0.626 | 0.504 | 0.624 | 0.466 | 0.494 |
| $H_{LU}$ | 0.510 | 0.509 | 0.900 | 0.899 | 0.840 | 0.843 | 0.543 | 0.532 | 0.634 | 0.634 | 0.499 | 0.504 | 0.556 | 0.558 | 0.671 | 0.674 | 0.705 | 0.709 | 0.448 | 0.445 |
| $H_{LB}$ | 0.508 | 0.511 | 0.863 | 0.789 | 0.840 | 0.798 | 0.665 | 0.724 | 0.608 | 0.622 | 0.544 | 0.539 | 0.613 | 0.719 | 0.633 | 0.656 | 0.457 | 0.579 | 0.438 | 0.448 |
| $DH_L$ | 0.508 | 0.505 | 0.820 | 0.863 | 0.757 | 0.798 | 0.472 | 0.380 | 0.612 | 0.602 | 0.469 | 0.478 | 0.460 | 0.361 | 0.643 | 0.638 | 0.723 | 0.664 | 0.461 | 0.453 |
| rel. $H_L$ | 0.502 | 0.507 | 0.619 | 0.505 | 0.677 | 0.568 | 0.689 | 0.777 | 0.545 | 0.554 | 0.555 | 0.550 | 0.644 | 0.813 | 0.550 | 0.568 | 0.352 | 0.469 | 0.465 | 0.478 |
| $Ar_{LU}$ | **0.522** | **0.521** | 0.836 | 0.829 | 0.877 | 0.890 | 0.510 | 0.501 | 0.652 | 0.650 | 0.471 | 0.478 | 0.554 | 0.562 | **0.725** | **0.727** | 0.292 | 0.307 | 0.422 | 0.417 |
| $Ar_{LB}$ | 0.510 | 0.513 | 0.788 | 0.767 | 0.852 | 0.861 | 0.578 | 0.630 | 0.586 | 0.604 | 0.495 | 0.489 | 0.621 | 0.720 | 0.679 | 0.695 | 0.371 | 0.422 | 0.412 | 0.427 |
| $DAr_L$ | 0.522 | 0.520 | 0.769 | 0.788 | 0.805 | 0.826 | 0.489 | 0.436 | 0.649 | 0.640 | 0.475 | 0.481 | 0.459 | 0.365 | 0.683 | 0.685 | 0.320 | 0.299 | 0.440 | 0.427 |
| rel. $Ar_L$ | 0.496 | 0.501 | 0.562 | 0.533 | 0.671 | 0.549 | 0.582 | 0.646 | 0.535 | 0.556 | 0.512 | 0.511 | 0.643 | 0.801 | 0.564 | 0.574 | 0.470 | 0.560 | 0.458 | 0.472 |
| $D_{LU}$ | 0.487 | 0.487 | 0.731 | 0.730 | 0.640 | 0.645 | 0.524 | 0.530 | 0.421 | 0.421 | 0.481 | 0.499 | 0.905 | 0.898 | 0.584 | 0.586 | 0.520 | 0.521 | 0.456 | 0.456 |
| $D_{LB}$ | 0.497 | 0.491 | 0.712 | 0.742 | 0.705 | 0.680 | 0.516 | 0.562 | 0.458 | 0.451 | 0.477 | 0.491 | 0.750 | 0.543 | 0.589 | 0.595 | 0.519 | 0.516 | 0.455 | 0.477 |
| $DD_L$ | 0.487 | 0.489 | 0.683 | 0.654 | 0.579 | 0.590 | 0.510 | 0.485 | 0.420 | 0.419 | 0.489 | 0.511 | 0.909 | 0.921 | 0.555 | 0.558 | 0.493 | 0.494 | 0.463 | 0.454 |
| rel. $D_L$ | 0.495 | 0.489 | 0.666 | 0.698 | 0.699 | 0.661 | 0.510 | 0.557 | 0.461 | 0.454 | 0.485 | 0.498 | 0.632 | 0.458 | 0.572 | 0.585 | 0.477 | 0.481 | 0.463 | 0.479 |
| $Ac_{LU}$ | 0.496 | 0.496 | 0.871 | 0.869 | 0.731 | 0.737 | 0.675 | 0.680 | 0.528 | 0.525 | 0.518 | 0.515 | 0.502 | 0.511 | 0.586 | 0.583 | 0.563 | 0.572 | 0.493 | 0.491 |
| $Ac_{LB}$ | 0.495 | 0.498 | 0.870 | 0.870 | 0.739 | 0.766 | 0.569 | 0.633 | 0.589 | 0.549 | 0.464 | 0.468 | 0.528 | 0.530 | 0.580 | 0.586 | 0.595 | 0.617 | 0.475 | 0.502 |

| | | | | | | | | | | | | | | | | | | | | |
|---|---|---|---|---|---|---|---|---|---|---|---|---|---|---|---|---|---|---|---|---|
| DAc$_{L}$ | 0.499 | 0.496 | 0.740 | 0.723 | 0.646 | 0.629 | 0.659 | 0.630 | 0.491 | 0.518 | 0.550 | 0.548 | 0.477 | 0.498 | 0.539 | 0.538 | 0.529 | 0.512 | 0.505 | 0.491 |
| rel. Ac$_{L}$ | 0.497 | 0.500 | 0.754 | 0.724 | 0.655 | 0.700 | 0.523 | 0.570 | 0.593 | 0.545 | 0.458 | 0.470 | 0.540 | 0.528 | 0.548 | 0.556 | 0.575 | 0.597 | 0.476 | 0.513 |
| P$_{LU}$ | 0.511 | 0.510 | 0.688 | 0.686 | 0.618 | 0.611 | 0.495 | 0.494 | 0.491 | 0.492 | 0.459 | 0.459 | 0.542 | 0.577 | 0.539 | 0.539 | 0.504 | 0.503 | 0.481 | 0.482 |
| P$_{LB}$ | 0.503 | 0.505 | 0.608 | 0.599 | 0.549 | 0.585 | 0.496 | 0.498 | 0.498 | 0.497 | 0.474 | 0.480 | 0.497 | 0.491 | 0.526 | 0.536 | 0.512 | 0.486 | 0.485 | 0.493 |
| DP$_{L}$ | 0.510 | 0.509 | 0.610 | 0.609 | 0.585 | 0.550 | 0.496 | 0.494 | 0.491 | 0.491 | 0.461 | 0.462 | 0.546 | 0.581 | 0.524 | 0.511 | 0.487 | 0.507 | 0.482 | 0.482 |
| rel. P$_{L}$ | 0.503 | 0.505 | 0.615 | 0.605 | 0.552 | 0.589 | 0.496 | 0.498 | 0.498 | 0.497 | 0.474 | 0.480 | 0.497 | 0.491 | 0.527 | 0.537 | 0.514 | 0.485 | 0.485 | 0.493 |
| N$_{LU}$ | 0.500 | 0.500 | 0.519 | 0.519 | 0.665 | 0.665 | 0.493 | 0.493 | 0.476 | 0.476 | 0.492 | 0.495 | 0.501 | 0.501 | 0.599 | 0.599 | 0.595 | 0.595 | 0.492 | 0.492 |
| N$_{LB}$ | 0.500 | 0.500 | 0.518 | 0.520 | 0.666 | 0.664 | 0.493 | 0.495 | 0.477 | 0.476 | 0.492 | 0.492 | 0.503 | 0.504 | 0.594 | 0.593 | 0.594 | 0.591 | 0.492 | 0.496 |
| DN$_{L}$ | 0.500 | 0.500 | 0.519 | 0.518 | 0.664 | 0.666 | 0.493 | 0.493 | 0.476 | 0.476 | 0.492 | 0.492 | 0.499 | 0.499 | 0.597 | 0.598 | 0.594 | 0.591 | 0.492 | 0.491 |
| rel. N$_{L}$ | 0.500 | 0.500 | 0.518 | 0.520 | 0.667 | 0.664 | 0.493 | 0.495 | 0.477 | 0.476 | 0.493 | 0.493 | 0.504 | 0.504 | 0.594 | 0.593 | 0.594 | 0.591 | 0.492 | 0.496 |
| O$_{LU}$ | 0.477 | 0.477 | 0.953 | 0.952 | 0.897 | 0.897 | 0.645 | 0.633 | 0.555 | 0.559 | 0.604 | 0.609 | 0.931 | 0.933 | 0.507 | 0.505 | 0.657 | 0.655 | **0.611** | **0.608** |
| O$_{LB}$ | 0.494 | 0.494 | 0.906 | 0.923 | 0.885 | 0.858 | 0.680 | 0.698 | 0.589 | 0.567 | 0.556 | 0.550 | 0.893 | 0.932 | 0.546 | 0.541 | 0.623 | 0.635 | 0.598 | 0.583 |
| DO$_{L}$ | 0.472 | 0.471 | 0.942 | 0.940 | 0.865 | 0.885 | 0.576 | 0.543 | 0.528 | 0.548 | 0.611 | 0.615 | 0.901 | 0.850 | 0.483 | 0.478 | 0.628 | 0.623 | 0.602 | 0.604 |
| rel. O$_{L}$ | 0.509 | 0.512 | 0.517 | 0.659 | 0.730 | 0.632 | 0.657 | 0.679 | 0.574 | 0.534 | 0.498 | 0.460 | 0.693 | 0.806 | 0.570 | 0.570 | 0.564 | 0.572 | 0.532 | 0.509 |
| DT$_{RL}$ | 0.483 | 0.482 | 0.971 | 0.973 | 0.957 | 0.962 | 0.775 | 0.696 | 0.638 | 0.635 | 0.629 | 0.645 | 0.918 | 0.880 | 0.674 | 0.668 | **0.860** | 0.822 | 0.549 | 0.543 |
| rel. T$_{RL}$ | 0.498 | 0.498 | 0.365 | 0.388 | 0.567 | 0.556 | 0.696 | 0.714 | 0.502 | 0.536 | 0.471 | 0.468 | 0.578 | 0.781 | 0.548 | 0.565 | 0.456 | 0.581 | 0.469 | 0.477 |
| DH$_{RL}$ | 0.501 | 0.499 | 0.960 | 0.954 | 0.922 | 0.941 | 0.652 | 0.545 | **0.675** | 0.647 | 0.545 | 0.539 | 0.697 | 0.633 | 0.699 | 0.662 | 0.830 | 0.775 | 0.483 | 0.472 |
| rel. H$_{RL}$ | 0.491 | 0.492 | 0.415 | 0.377 | 0.504 | 0.381 | 0.569 | 0.657 | 0.413 | 0.507 | 0.523 | 0.544 | 0.485 | 0.679 | 0.436 | 0.529 | 0.307 | 0.525 | 0.460 | 0.486 |
| DAr$_{RL}$ | 0.520 | 0.519 | 0.903 | 0.914 | 0.912 | 0.906 | 0.638 | 0.588 | 0.651 | 0.640 | 0.496 | 0.508 | 0.567 | 0.484 | 0.706 | 0.700 | 0.415 | 0.416 | 0.454 | 0.486 |
| rel. Ar$_{RL}$ | 0.482 | 0.488 | 0.339 | 0.401 | 0.397 | 0.457 | 0.492 | 0.528 | 0.477 | 0.481 | 0.527 | 0.526 | 0.572 | 0.611 | 0.496 | 0.475 | 0.453 | 0.575 | 0.455 | 0.490 |
| DD$_{RL}$ | 0.487 | 0.490 | 0.868 | 0.903 | 0.845 | 0.806 | 0.521 | 0.498 | 0.437 | 0.481 | 0.500 | 0.514 | 0.928 | 0.934 | 0.595 | 0.628 | 0.605 | 0.669 | 0.511 | 0.501 |
| rel. D$_{RL}$ | 0.502 | 0.500 | 0.323 | 0.268 | 0.245 | 0.407 | 0.694 | 0.605 | 0.532 | 0.500 | 0.522 | 0.496 | 0.391 | 0.320 | 0.465 | 0.440 | 0.549 | 0.404 | 0.484 | 0.468 |
| DAc$_{RL}$ | 0.497 | 0.493 | 0.821 | 0.813 | 0.708 | 0.695 | 0.650 | 0.634 | 0.533 | 0.541 | 0.553 | 0.575 | 0.563 | 0.584 | 0.552 | 0.558 | 0.547 | 0.543 | 0.506 | 0.500 |
| rel. Ac$_{RL}$ | 0.501 | 0.503 | 0.803 | 0.672 | 0.653 | 0.695 | 0.495 | 0.508 | 0.588 | 0.523 | 0.459 | 0.460 | 0.449 | 0.517 | 0.569 | 0.550 | 0.593 | 0.612 | 0.478 | 0.514 |
| DP$_{RL}$ | 0.510 | 0.509 | 0.833 | 0.835 | 0.740 | 0.802 | 0.499 | 0.495 | 0.458 | 0.506 | 0.511 | 0.492 | 0.649 | 0.632 | 0.514 | 0.646 | 0.586 | 0.509 | 0.494 | 0.481 |
| rel. P$_{RL}$ | 0.503 | 0.505 | 0.492 | 0.345 | 0.495 | 0.534 | 0.498 | 0.499 | 0.524 | 0.524 | 0.542 | 0.519 | 0.535 | 0.454 | 0.520 | 0.486 | 0.556 | 0.486 | 0.494 | 0.501 |
| DN$_{RL}$ | 0.499 | 0.498 | 0.923 | 0.837 | 0.863 | 0.848 | 0.613 | 0.573 | 0.473 | 0.489 | 0.493 | 0.490 | 0.532 | 0.674 | 0.625 | 0.658 | 0.596 | 0.589 | 0.498 | 0.502 |
| rel. N$_{RL}$ | 0.497 | 0.496 | 0.332 | 0.282 | 0.481 | 0.499 | 0.662 | 0.575 | 0.481 | 0.501 | 0.521 | 0.553 | 0.509 | 0.569 | 0.589 | 0.564 | 0.597 | 0.583 | 0.526 | 0.497 |
| DO$_{RL}$ | 0.472 | 0.471 | 0.942 | 0.940 | 0.867 | 0.886 | 0.577 | 0.544 | 0.532 | 0.550 | 0.611 | 0.615 | 0.901 | 0.851 | 0.483 | 0.478 | 0.630 | 0.632 | 0.602 | 0.604 |
| rel. O$_{RL}$ | 0.509 | 0.513 | 0.516 | 0.658 | 0.724 | 0.617 | 0.657 | 0.679 | 0.573 | 0.531 | 0.498 | 0.460 | 0.693 | 0.811 | 0.570 | 0.570 | 0.562 | 0.565 | 0.532 | 0.510 |

**Table S3.** The percent improvement or depreciation in the AUC values with respect to the Surflex scoring function for the 10 PPI targets (20 protein structures) using different SASA scoring methods. The positive value indicates a percent increase in the AUC, while the negative value indicates a percent decrease in the AUC. The AUC values with an improvement greater than 10% are shown in bold and are highlighted in green.

| SASA descriptor | BAZ2B | | BCL2 | | BCLXL | | BRD4-1 | | CREBBP | | HIV-IN | | XIAP | | MCL1 | | MDM2 | | Menin | |
|---|---|---|---|---|---|---|---|---|---|---|---|---|---|---|---|---|---|---|---|---|
| | 4XUA | 5E73 | 2O21 | 4LVT | 3INQ | 3WIZ | 5D3L | 5KU3 | 5EIC | 5MMG | 4CFD | 4CHO | 1TFT | 5C3H | 5FC4 | 5MES | 4ODF | 4ZFI | 5DB2 | 6B41 |
| $T_{RU}$ | -1.19 | 0.57 | **12.59** | 9.02 | **11.89** | **15.43** | **16.33** | **73.29** | -1.61 | -3.47 | -3.44 | -2.85 | -3.52 | -6.44 | **10.58** | 5.88 | **14.54** | **30.94** | -2.38 | 2.63 |
| $T_{RB}$ | -1.07 | 0.48 | 4.74 | 1.08 | 2.89 | 8.35 | 6.64 | **42.23** | -13.99 | -8.21 | -7.13 | -6.45 | -12.56 | -15.05 | -3.88 | -3.84 | -3.01 | **16.54** | -5.38 | -2.97 |
| $\Delta T_R$ | -0.57 | 1.52 | **13.92** | **10.32** | **13.77** | **16.97** | **15.73** | **85.31** | 9.64 | -4.50 | -3.39 | -1.93 | -1.83 | -2.62 | **15.33** | **13.58** | **29.64** | **41.23** | -0.70 | 4.79 |
| rel. $T_R$ | -0.07 | 0.99 | -81.47 | -85.64 | -80.23 | -62.23 | -21.03 | -7.64 | -35.07 | -23.99 | -24.28 | -21.28 | -59.47 | -61.02 | -37.75 | -29.21 | -38.06 | -19.71 | -11.31 | -9.64 |
| $H_{RU}$ | -0.89 | 0.79 | **11.43** | 8.27 | **11.15** | **14.30** | **13.48** | **68.65** | -3.93 | -5.11 | 2.43 | 0.53 | -9.50 | -5.54 | **11.79** | 3.06 | **12.94** | **30.45** | -3.68 | 3.13 |
| $H_{RB}$ | -0.76 | 0.76 | 0.15 | -7.70 | 2.12 | 7.10 | -6.95 | **35.80** | -19.32 | -10.92 | -9.06 | -5.49 | -24.94 | -18.10 | -3.77 | -4.54 | -20.43 | **16.92** | -7.67 | 0.16 |
| $\Delta H_R$ | -0.42 | 1.85 | **12.90** | 8.59 | **11.92** | **15.00** | **17.94** | **83.23** | **10.06** | -5.19 | 5.38 | -0.78 | -4.19 | 0.42 | **16.10** | 4.75 | **29.94** | **39.06** | -1.09 | 4.01 |
| rel. $H_R$ | -0.26 | 1.11 | -64.30 | -61.25 | -59.75 | -62.69 | -37.89 | 2.15 | -36.07 | -23.06 | -27.06 | -19.36 | -72.29 | -60.58 | -32.53 | -17.55 | -46.18 | -3.28 | -12.34 | -3.31 |
| $Ar_{RU}$ | -0.85 | 2.44 | -3.84 | 2.84 | -8.58 | -0.28 | **15.48** | **78.29** | 0.07 | -15.55 | -11.50 | -13.77 | -27.24 | -25.21 | -2.64 | -6.77 | 1.40 | **17.97** | -0.10 | -1.27 |
| $Ar_{RB}$ | -1.08 | 1.70 | -45.95 | -28.04 | -45.38 | -27.86 | -14.01 | **15.06** | -12.31 | -18.06 | -14.11 | -13.58 | -34.43 | -56.99 | -18.48 | -13.23 | -18.53 | 4.03 | -12.17 | -0.53 |
| $\Delta Ar_R$ | 0.78 | 4.15 | 8.12 | 5.67 | 6.21 | 5.46 | **15.11** | **87.47** | -9.78 | -18.06 | -12.04 | -13.12 | -26.13 | -23.64 | 5.45 | -0.49 | **22.04** | **23.57** | -3.78 | -4.10 |
| rel. $Ar_R$ | -0.27 | 1.11 | -76.42 | -64.07 | -75.37 | -54.49 | -49.22 | -33.50 | -16.90 | -24.00 | -18.90 | -24.72 | -53.90 | -62.34 | -25.75 | -25.94 | -28.29 | -18.80 | -12.55 | -1.58 |
| $D_{RU}$ | 0.92 | 2.37 | -1.37 | 1.21 | -0.71 | -0.31 | 8.86 | **45.72** | -14.66 | -4.06 | -11.84 | -18.20 | -15.98 | -12.84 | -2.63 | 3.03 | 4.22 | 8.05 | 0.04 | 3.81 |
| $D_{RB}$ | 1.82 | 3.43 | -26.68 | -34.56 | -40.65 | -25.03 | 6.79 | **42.22** | -17.56 | -20.28 | -14.02 | -20.11 | -25.77 | -34.53 | -15.41 | -7.42 | 2.37 | -17.65 | -7.50 | -9.32 |
| $\Delta D_R$ | -0.35 | 1.66 | 2.81 | 4.71 | 3.32 | 6.17 | -29.94 | **21.93** | -13.63 | -1.95 | -17.34 | -16.46 | -14.60 | -20.68 | -0.10 | 5.78 | -2.65 | **30.70** | 0.47 | 7.44 |
| rel. $D_R$ | 2.38 | 3.64 | -65.25 | -77.68 | -79.23 | -59.68 | 2.10 | **36.54** | -19.59 | -34.89 | -17.86 | -20.29 | -39.77 | -49.08 | -25.55 | -29.39 | -22.56 | -37.16 | -12.53 | -13.25 |
| $Ac_{RU}$ | 1.28 | 2.49 | 6.43 | -5.95 | -8.33 | 1.05 | -29.75 | **17.00** | 3.67 | -11.71 | -12.60 | -7.12 | -20.77 | -22.74 | 2.79 | -3.33 | 4.74 | **11.08** | -4.99 | 6.80 |
| $Ac_{RB}$ | 2.10 | 3.67 | -0.96 | -17.16 | -13.29 | -20.91 | -25.44 | 8.53 | -0.96 | -15.61 | -17.24 | -15.37 | -55.75 | -36.62 | -3.19 | -13.83 | -1.38 | 6.71 | -5.22 | 3.38 |
| $\Delta Ac_R$ | 0.67 | 1.74 | 7.12 | -6.76 | -5.97 | -0.24 | -34.05 | **29.86** | 5.05 | -10.51 | -12.27 | -5.73 | -3.57 | -13.54 | 2.46 | 2.30 | -6.01 | 7.30 | -7.00 | 4.41 |
| rel. $Ac_R$ | 2.74 | 4.57 | -14.89 | -44.89 | -34.94 | -43.03 | -20.10 | 0.87 | -10.21 | -23.05 | -26.59 | -26.05 | -65.18 | -45.89 | -17.29 | -22.18 | -10.73 | -1.28 | -6.66 | -0.64 |
| $P_{RU}$ | 1.86 | 3.72 | -14.21 | -12.40 | -17.14 | -1.87 | -27.88 | **17.67** | -21.46 | -19.79 | -14.74 | -15.42 | -13.63 | -23.92 | -14.24 | 1.59 | -7.31 | -11.71 | -7.73 | -1.70 |
| $P_{RB}$ | 1.86 | 3.72 | -26.61 | -27.59 | -26.66 | -7.54 | -27.91 | **17.67** | -20.51 | -19.58 | -13.16 | -15.39 | -11.65 | -27.81 | -11.90 | -8.79 | -8.06 | -11.71 | -8.44 | -1.63 |

| | | | | | | | | | | | | | | | | | | | | |
|---|---|---|---|---|---|---|---|---|---|---|---|---|---|---|---|---|---|---|---|---|
| $\Delta P_R$ | 1.85 | 3.73 | -3.03 | -5.84 | -13.38 | -2.39 | -27.93 | **17.53** | -23.45 | -23.47 | -17.36 | -16.92 | -27.05 | -29.27 | -13.22 | 9.43 | -8.20 | -11.57 | -7.66 | -3.33 |
| rel. $P_R$ | 1.86 | 3.72 | -42.64 | -61.10 | -40.46 | -35.69 | -27.95 | **17.67** | -13.60 | -20.89 | -13.63 | -15.46 | -39.51 | -48.94 | -12.35 | -17.23 | -12.79 | -11.72 | -8.24 | -1.63 |
| $N_{RU}$ | 1.20 | 2.93 | 6.31 | -8.62 | -1.33 | -3.31 | 4.62 | **37.71** | -17.27 | -21.25 | -16.47 | -16.69 | -41.05 | -17.00 | -9.95 | -4.85 | -21.09 | -11.99 | -5.95 | -3.59 |
| $N_{RB}$ | 1.21 | 3.02 | -5.02 | -14.35 | -8.15 | -7.04 | 4.33 | **36.95** | -17.17 | -21.24 | -14.06 | -14.24 | -43.33 | -19.25 | -11.18 | -8.39 | -21.09 | -12.01 | -4.32 | -5.20 |
| $\Delta N_R$ | 1.69 | 3.24 | 8.22 | -4.73 | -5.53 | -1.14 | -10.54 | **36.69** | -17.74 | -22.24 | -21.61 | -20.17 | -39.03 | -22.74 | -9.11 | -1.06 | -21.11 | -11.87 | -7.80 | -1.47 |
| rel. $N_R$ | 1.21 | 3.04 | -61.40 | -68.73 | -38.61 | -36.67 | -5.21 | **35.29** | -17.16 | -21.47 | -13.78 | -11.46 | -43.98 | -36.48 | -11.18 | -8.41 | -21.09 | -12.02 | -5.33 | -6.17 |
| $O_{RU}$ | -0.49 | 1.67 | -13.89 | -9.28 | -9.77 | -9.37 | -22.78 | **26.71** | -6.78 | -17.13 | -14.96 | -15.88 | -40.41 | -31.68 | -8.93 | -15.26 | -7.99 | 9.51 | -6.17 | -2.87 |
| $O_{RB}$ | 0.36 | 2.41 | -28.40 | -37.24 | -24.51 | -39.45 | -38.78 | 9.19 | -17.81 | -21.14 | -20.08 | -24.78 | -59.46 | -37.20 | -10.82 | -15.26 | -26.32 | -7.96 | -11.46 | -4.88 |
| $\Delta O_R$ | -0.84 | 1.46 | -14.64 | -10.98 | -13.06 | -14.39 | -8.16 | **36.86** | 1.65 | -16.87 | -14.63 | -11.44 | -17.21 | -27.32 | -10.90 | -15.25 | -2.60 | **15.36** | -4.40 | -1.27 |
| rel. $O_R$ | 1.33 | 3.11 | -28.67 | -39.15 | -38.94 | -43.99 | -38.78 | 9.19 | -23.73 | -23.04 | -20.09 | -24.78 | -68.29 | -40.98 | -10.80 | -15.26 | -26.79 | -20.57 | -11.50 | -5.00 |
| $T_{LU}$ | -0.83 | 0.87 | **13.99** | **10.34** | **14.88** | **17.65** | 8.08 | **74.70** | 5.55 | -3.10 | -3.20 | -2.38 | 6.11 | 5.84 | **18.07** | **18.07** | **31.22** | **48.00** | -0.17 | 4.93 |
| $T_{LB}$ | 1.46 | 3.57 | **13.23** | 9.40 | **13.81** | **15.71** | 1.99 | **82.28** | 3.06 | -9.76 | -14.37 | -15.65 | -0.93 | 3.77 | **14.47** | **15.62** | 0.99 | **33.15** | -4.86 | 2.45 |
| $\Delta T_L$ | -2.18 | -0.78 | **12.48** | 8.98 | **12.54** | **16.35** | 2.10 | **36.17** | 0.22 | -6.98 | 1.79 | 3.19 | 4.42 | -2.10 | **13.06** | **10.75** | **31.52** | **41.88** | 0.47 | 5.06 |
| rel. $T_L$ | 2.91 | 5.38 | -19.99 | -18.62 | -1.25 | -10.82 | -4.22 | **78.76** | -2.66 | -17.87 | -20.49 | -25.82 | -22.55 | -6.59 | 5.89 | 6.12 | -21.25 | 9.41 | -14.97 | -4.50 |
| $H_{LU}$ | 3.84 | 5.67 | 5.03 | 1.42 | -0.43 | 2.37 | -22.02 | **24.82** | 4.93 | -4.22 | -20.47 | -20.12 | -37.41 | -37.45 | **14.75** | **14.17** | **10.15** | **24.38** | -18.25 | -13.99 |
| $H_{LB}$ | 3.48 | 6.10 | 0.75 | -10.92 | -0.49 | -3.03 | -4.43 | **70.00** | 0.74 | -6.02 | -13.29 | -14.56 | -30.95 | -19.40 | 8.18 | **11.24** | -28.64 | 1.65 | -20.15 | -13.28 |
| $\Delta H_L$ | 3.51 | 4.73 | -4.35 | -2.63 | -10.28 | -3.05 | -32.14 | -10.88 | 1.24 | -9.03 | -25.15 | -24.32 | -48.25 | -59.57 | 9.99 | 8.10 | **12.93** | **16.51** | -15.84 | -12.47 |
| rel. $H_L$ | 2.22 | 5.23 | -27.78 | -43.01 | -19.83 | -31.02 | -1.06 | **82.46** | -9.85 | -16.26 | -11.48 | -12.81 | -27.48 | -8.86 | -5.92 | -3.68 | -44.93 | -17.68 | -15.20 | -7.56 |
| $Ar_{LU}$ | 6.37 | 8.18 | -2.40 | -6.46 | 3.88 | 8.08 | -26.72 | **17.64** | 8.01 | -1.78 | -24.87 | -24.18 | -37.58 | -36.97 | **23.89** | **23.29** | -54.39 | -46.23 | -23.02 | -19.32 |
| $Ar_{LB}$ | 3.87 | 6.45 | -8.01 | -13.39 | 1.01 | 4.61 | -16.97 | **47.96** | -2.91 | -8.69 | -21.11 | -22.58 | -30.01 | -19.26 | **16.06** | **17.80** | -42.04 | -26.01 | -24.75 | -17.38 |
| $\Delta Ar_L$ | 6.30 | 7.84 | -10.27 | -11.05 | -4.65 | 0.30 | -29.78 | 2.40 | 7.45 | -3.32 | -24.27 | -23.73 | -48.35 | -59.03 | **16.69** | **16.11** | -49.97 | -47.47 | -19.63 | -17.42 |
| rel. $Ar_L$ | 1.04 | 3.88 | -34.44 | -39.79 | -20.55 | -33.32 | -16.36 | **51.74** | -11.42 | -15.97 | -18.29 | -19.01 | -27.61 | -10.22 | -3.64 | -2.70 | -26.51 | -1.71 | -16.46 | -8.66 |
| $D_{LU}$ | -0.77 | 1.06 | -14.70 | -17.60 | -24.16 | -21.60 | -24.66 | **24.46** | -30.26 | -36.46 | -23.23 | -20.87 | 1.93 | 0.71 | -0.22 | -0.65 | -18.76 | -8.53 | -16.72 | -11.86 |
| $D_{LB}$ | 1.29 | 1.85 | -16.93 | -16.22 | -16.52 | -17.33 | -25.87 | **31.99** | -24.12 | -31.85 | -23.87 | -22.17 | -15.53 | -39.10 | 0.69 | 0.81 | -18.85 | -9.46 | -17.04 | -7.66 |
| $\Delta D_L$ | -0.75 | 1.52 | -20.30 | -26.23 | -31.42 | -28.34 | -26.71 | **13.93** | -30.53 | -36.76 | -21.95 | -18.99 | 2.39 | 3.26 | -5.18 | -5.49 | -22.92 | -13.29 | -15.48 | -12.24 |
| rel. $D_L$ | 0.78 | 1.38 | -22.34 | -21.21 | -17.14 | -19.66 | -26.69 | **30.67** | -23.59 | -31.41 | -22.72 | -21.11 | -28.83 | -48.71 | -2.27 | -0.92 | -25.54 | -15.67 | -15.57 | -7.38 |
| $Ac_{LU}$ | 1.09 | 2.89 | 1.62 | -1.91 | -13.34 | -10.41 | -3.05 | **59.57** | -12.51 | -20.70 | -17.39 | -18.41 | -43.52 | -42.75 | 0.16 | -1.19 | -12.08 | 0.29 | -9.99 | -5.04 |
| $Ac_{LB}$ | 0.88 | 3.35 | 1.47 | -1.78 | -12.46 | -6.95 | -18.26 | **48.59** | -2.41 | -17.02 | -26.07 | -25.79 | -40.57 | -40.62 | -0.84 | -0.66 | -7.11 | 8.21 | -13.34 | -2.96 |
| $\Delta Ac_L$ | 1.58 | 2.93 | -13.63 | -18.36 | -23.49 | -23.59 | -5.30 | **47.84** | -18.71 | -21.82 | -12.31 | -13.12 | -46.33 | -44.19 | -7.85 | -8.84 | -17.39 | -10.21 | -7.87 | -4.99 |
| rel. $Ac_L$ | 1.15 | 3.66 | -11.99 | -18.32 | -22.40 | -14.91 | -24.87 | **33.78** | -1.75 | -17.61 | -27.03 | -25.45 | -39.17 | -40.79 | -6.35 | -5.84 | -10.22 | 4.69 | -13.12 | -0.79 |

| | | | | | | | | | | | | | | | | | | | | |
|---|---|---|---|---|---|---|---|---|---|---|---|---|---|---|---|---|---|---|---|---|
| $P_{LU}$ | 4.07 | 5.90 | -19.67 | -22.53 | -26.74 | -25.71 | -28.86 | **15.88** | -18.64 | -25.75 | -26.75 | -27.29 | -39.00 | -35.32 | -7.90 | -8.62 | -21.28 | -11.78 | -12.30 | -6.82 |
| $P_{LB}$ | 2.50 | 4.76 | -29.07 | -32.39 | -34.92 | -28.86 | -28.74 | **16.95** | -17.61 | -24.87 | -24.41 | -23.96 | -44.05 | -44.99 | -10.07 | -9.18 | -20.03 | -14.82 | -11.55 | -4.61 |
| $\Delta P_{L}$ | 3.79 | 5.62 | -28.84 | -31.22 | -30.72 | -33.12 | -28.78 | **15.99** | -18.67 | -25.76 | -26.51 | -26.77 | -38.52 | -34.91 | -10.35 | -13.43 | -23.93 | -11.03 | -12.01 | -6.73 |
| rel. $P_{L}$ | 2.52 | 4.78 | -28.22 | -31.71 | -34.65 | -28.39 | -28.74 | **16.95** | -17.61 | -24.87 | -24.41 | -23.98 | -44.04 | -45.00 | -9.92 | -8.95 | -19.72 | -14.83 | -11.57 | -4.60 |
| $N_{LU}$ | 1.81 | 3.70 | -39.39 | -41.38 | -21.19 | -19.15 | -29.20 | **15.68** | -21.14 | -28.05 | -21.54 | -21.53 | -43.53 | -43.79 | 2.44 | 1.47 | -7.02 | 4.39 | -10.28 | -4.92 |
| $N_{LB}$ | 1.88 | 3.68 | -39.58 | -41.31 | -21.08 | -19.30 | -29.12 | **16.27** | -20.95 | -28.04 | -21.55 | -21.97 | -43.30 | -43.51 | 1.47 | 0.45 | -7.13 | 3.64 | -10.26 | -4.04 |
| $\Delta N_{L}$ | 1.78 | 3.66 | -39.40 | -41.48 | -21.36 | -19.11 | -29.20 | **15.67** | -21.16 | -28.07 | -21.50 | -22.01 | -43.81 | -44.06 | 2.08 | 1.36 | -7.17 | 3.68 | -10.26 | -5.08 |
| rel. $N_{L}$ | 1.88 | 3.68 | -39.57 | -41.30 | -21.01 | -19.28 | -29.12 | **16.27** | -20.95 | -28.04 | -21.38 | -21.92 | -43.29 | -43.50 | 1.48 | 0.49 | -7.13 | 3.64 | -10.25 | -4.03 |
| $O_{LU}$ | -2.90 | -1.11 | **11.17** | 7.47 | 6.22 | 9.01 | -7.35 | **48.70** | -8.18 | -15.49 | -3.74 | -3.43 | 4.81 | 4.61 | -13.37 | -14.46 | 2.72 | **14.93** | **11.57** | **17.65** |
| $O_{LB}$ | 0.54 | 2.45 | 5.72 | 4.14 | 4.89 | 4.22 | -2.36 | **63.75** | -2.55 | -14.35 | -11.25 | -12.91 | 0.59 | 4.46 | -6.71 | -8.39 | -2.72 | **11.38** | 9.14 | **12.67** |
| $\Delta O_{L}$ | -3.80 | -2.21 | 9.97 | 6.10 | 2.50 | 7.48 | -17.22 | **27.55** | -12.55 | -17.18 | -2.52 | -2.58 | 1.45 | -4.74 | -17.43 | -18.90 | -1.80 | 9.29 | 9.77 | **16.82** |
| rel. $O_{L}$ | 3.59 | 6.18 | -39.62 | -25.59 | -13.50 | -23.27 | -5.57 | **59.43** | -4.89 | -19.41 | -20.58 | -27.04 | -21.91 | -9.64 | -2.52 | -3.33 | -11.86 | 0.42 | -2.92 | -1.54 |
| $\Delta T_{RL}$ | -1.66 | -0.07 | **13.35** | 9.87 | **13.39** | **16.88** | **11.30** | **63.37** | 5.67 | -4.14 | 0.30 | 2.18 | 3.39 | -1.36 | **15.24** | **13.20** | **34.39** | **44.29** | 0.15 | 5.04 |
| rel. $T_{RL}$ | 1.40 | 3.28 | -57.41 | -56.23 | -32.81 | -32.42 | 0.02 | **67.58** | -16.95 | -19.11 | -24.89 | -25.78 | -34.94 | -12.46 | -6.31 | -4.22 | -28.82 | 1.96 | -14.44 | -7.77 |
| $\Delta H_{RL}$ | 2.02 | 3.62 | **12.07** | 7.70 | 9.24 | **14.35** | -6.27 | **27.89** | **11.80** | -2.33 | -13.10 | -14.57 | -21.45 | -29.04 | **19.41** | **12.23** | **29.68** | **36.05** | -11.93 | -8.73 |
| rel. $H_{RL}$ | -0.09 | 2.17 | -51.61 | -57.41 | -40.25 | -53.70 | -18.27 | **54.21** | -31.62 | -23.43 | -16.59 | -13.72 | -45.36 | -23.84 | -25.55 | -10.30 | -51.97 | -7.93 | -16.13 | -5.95 |
| $\Delta Ar_{RL}$ | 5.87 | 7.71 | 5.39 | 3.17 | 8.11 | **10.06** | -8.34 | **38.08** | 7.74 | -3.39 | -20.82 | -19.57 | -36.14 | -45.75 | **20.74** | **18.71** | -35.14 | -27.10 | -17.24 | -5.95 |
| rel. $Ar_{RL}$ | -1.81 | 1.18 | -60.47 | -54.72 | -52.97 | -44.53 | -29.34 | **23.93** | -20.98 | -27.30 | -15.96 | -16.64 | -35.54 | -31.55 | -15.18 | -19.53 | -29.15 | 0.84 | -16.91 | -5.26 |
| $\Delta D_{RL}$ | -0.89 | 1.67 | 1.26 | 1.89 | 0.13 | -2.03 | -25.15 | **16.84** | -27.60 | -27.30 | -20.19 | -18.61 | 4.49 | 4.68 | 1.77 | 6.52 | -5.43 | **17.34** | -6.71 | -3.18 |
| rel. $D_{RL}$ | 2.28 | 3.68 | -62.27 | -69.77 | -70.96 | -50.59 | -0.30 | **42.12** | -11.89 | -24.52 | -16.67 | -21.46 | -55.96 | -64.08 | -20.44 | -25.46 | -14.16 | -29.15 | -11.64 | -9.39 |
| $\Delta Ac_{RL}$ | 1.20 | 2.38 | -4.21 | -8.25 | -16.07 | -15.58 | -6.55 | **48.82** | -11.80 | -18.22 | -11.73 | -8.91 | -36.55 | -34.55 | -5.60 | -5.46 | -14.60 | -4.77 | -7.64 | -3.28 |
| rel. $Ac_{RL}$ | 2.04 | 4.37 | -6.34 | -24.19 | -22.61 | -15.58 | -28.86 | **19.24** | -2.71 | -20.94 | -26.82 | -27.09 | -49.46 | -41.99 | -2.65 | -6.86 | -7.39 | 7.28 | -12.71 | -0.52 |
| $\Delta P_{RL}$ | 3.80 | 5.63 | -2.81 | -5.71 | -12.33 | -2.56 | -28.37 | **16.15** | -24.13 | -23.60 | -18.48 | -22.04 | -26.91 | -29.16 | -12.14 | 9.49 | -8.42 | -10.66 | -9.93 | -6.94 |
| rel. $P_{RL}$ | 2.54 | 4.77 | -42.65 | -61.05 | -41.39 | -35.17 | -28.42 | **17.25** | -13.31 | -20.85 | -13.59 | -17.74 | -39.79 | -49.07 | -11.07 | -17.60 | -13.12 | -14.80 | -9.93 | -3.11 |
| $\Delta N_{RL}$ | 1.65 | 3.22 | 7.73 | -5.50 | 2.30 | 3.03 | -11.90 | **34.47** | -21.66 | -26.11 | -21.42 | -22.41 | -40.11 | -24.47 | 6.81 | **11.57** | -6.81 | 3.40 | -9.12 | -2.82 |
| rel. $N_{RL}$ | 1.24 | 2.99 | -61.30 | -68.22 | -42.95 | -39.36 | -4.84 | **35.09** | -20.43 | -24.29 | -16.93 | -12.42 | -42.64 | -36.20 | 0.66 | -4.43 | -6.78 | 2.29 | -4.11 | -3.96 |
| $\Delta O_{RL}$ | -3.79 | -2.19 | 9.95 | 6.15 | 2.68 | 7.65 | -17.10 | **27.59** | -11.85 | -16.85 | -2.51 | -2.55 | 1.45 | -4.63 | -17.41 | -18.90 | -1.50 | **10.81** | 9.79 | **16.81** |
| rel. $O_{RL}$ | 3.65 | 6.35 | -39.84 | -25.78 | -14.27 | -25.07 | -5.67 | **59.41** | -5.20 | -19.81 | -20.58 | -27.11 | -21.92 | -9.10 | -2.54 | -3.33 | -12.14 | -0.89 | -2.92 | -1.44 |

**Table S4.** The BEDROC values ($\alpha$ = 20) for the datasets screened using SASA descriptors. The best BEDROC score corresponding to each target protein (across columns) is indicated in bold and highlighted in green.

| SASA descriptor | BAZ2B | | BCL2 | | BCLXL | | BRD4-1 | | CREBBP | | HIV-IN | | XIAP | | MCL1 | | MDM2 | | Menin | |
|---|---|---|---|---|---|---|---|---|---|---|---|---|---|---|---|---|---|---|---|---|
| | 4XUA | 5E73 | 2O21 | 4LVT | 3INQ | 3WIZ | 5D3L | 5KU3 | 5EIC | 5MMG | 4CFD | 4CHO | 1TFT | 5C3H | 5FC4 | 5MES | 4ODF | 4ZFI | 5DB2 | 6B41 |
| Surflex | 0.120 | 0.120 | 0.641 | 0.701 | 0.523 | 0.500 | 0.672 | 0.254 | 0.100 | **0.160** | 0.604 | **0.639** | **0.767** | **0.704** | 0.170 | 0.150 | 0.842 | 0.600 | 0.104 | 0.065 |
| $T_{RU}$ | 0.125 | 0.131 | 0.882 | 0.872 | 0.792 | 0.809 | 0.753 | 0.596 | 0.093 | 0.119 | 0.398 | 0.443 | 0.431 | 0.385 | 0.260 | 0.223 | 0.689 | 0.662 | 0.111 | 0.108 |
| $T_{RB}$ | 0.123 | 0.132 | 0.640 | 0.582 | 0.543 | 0.592 | 0.766 | 0.479 | 0.076 | 0.117 | 0.450 | 0.530 | 0.300 | 0.288 | 0.128 | 0.143 | 0.498 | 0.579 | 0.084 | 0.076 |
| $DT_R$ | 0.127 | 0.132 | 0.932 | 0.927 | 0.864 | 0.864 | 0.721 | 0.677 | 0.120 | 0.107 | 0.370 | 0.381 | 0.410 | 0.425 | 0.290 | 0.283 | 0.742 | 0.699 | 0.126 | 0.136 |
| rel. $T_R$ | 0.133 | 0.142 | 0.002 | 0.003 | 0.006 | 0.006 | 0.385 | 0.143 | 0.031 | 0.051 | 0.327 | 0.302 | 0.016 | 0.012 | 0.039 | 0.048 | 0.095 | 0.246 | 0.047 | 0.049 |
| $H_{RU}$ | 0.125 | 0.132 | 0.847 | 0.864 | 0.740 | 0.767 | 0.754 | 0.565 | 0.089 | 0.125 | 0.468 | 0.436 | 0.261 | 0.367 | 0.236 | 0.204 | 0.635 | 0.648 | 0.100 | 0.104 |
| $H_{RB}$ | 0.123 | 0.132 | 0.450 | 0.368 | 0.466 | 0.536 | 0.593 | 0.434 | 0.072 | 0.125 | 0.504 | 0.475 | 0.156 | 0.259 | 0.104 | 0.139 | 0.315 | 0.535 | 0.078 | 0.091 |
| $DH_R$ | 0.128 | 0.132 | 0.911 | 0.872 | 0.801 | 0.786 | 0.767 | 0.646 | 0.120 | 0.101 | 0.412 | 0.403 | 0.346 | 0.492 | 0.280 | 0.226 | 0.744 | 0.714 | 0.116 | 0.112 |
| rel. $H_R$ | 0.125 | 0.132 | 0.004 | 0.006 | 0.026 | 0.009 | 0.135 | 0.166 | 0.035 | 0.047 | 0.323 | 0.307 | 0.002 | 0.019 | 0.043 | 0.063 | 0.071 | 0.299 | 0.052 | 0.067 |
| $Ar_{RU}$ | 0.102 | 0.124 | 0.362 | 0.656 | 0.152 | 0.396 | 0.701 | 0.649 | 0.104 | 0.077 | 0.325 | 0.344 | 0.113 | 0.076 | 0.125 | 0.125 | 0.574 | 0.530 | 0.101 | 0.082 |
| $Ar_{RB}$ | 0.124 | 0.128 | 0.042 | 0.049 | 0.010 | 0.189 | 0.346 | 0.334 | 0.093 | 0.080 | 0.332 | 0.364 | 0.072 | 0.013 | 0.092 | 0.085 | 0.378 | 0.350 | 0.066 | 0.076 |
| $DAr_R$ | 0.132 | 0.134 | 0.677 | 0.759 | 0.588 | 0.453 | 0.752 | 0.727 | 0.071 | 0.076 | 0.362 | 0.354 | 0.156 | 0.178 | 0.199 | 0.171 | 0.671 | 0.613 | 0.093 | 0.084 |
| rel. $Ar_R$ | 0.125 | 0.137 | 0.006 | 0.008 | 0.005 | 0.028 | 0.089 | 0.108 | 0.054 | 0.046 | 0.377 | 0.274 | 0.013 | 0.004 | 0.042 | 0.038 | 0.174 | 0.180 | 0.064 | 0.058 |
| $D_{RU}$ | 0.097 | 0.103 | 0.556 | 0.584 | 0.522 | 0.428 | **0.866** | 0.527 | 0.066 | 0.108 | 0.396 | 0.412 | 0.293 | 0.150 | 0.136 | 0.173 | 0.559 | 0.487 | 0.084 | 0.106 |
| $D_{RB}$ | 0.145 | 0.140 | 0.356 | 0.072 | 0.044 | 0.108 | 0.849 | 0.531 | 0.065 | 0.070 | 0.366 | 0.431 | 0.160 | 0.091 | 0.068 | 0.089 | 0.479 | 0.363 | 0.066 | 0.050 |
| $DD_R$ | 0.123 | 0.128 | 0.483 | 0.654 | 0.489 | 0.510 | 0.434 | 0.604 | 0.062 | 0.144 | 0.398 | 0.404 | 0.259 | 0.171 | 0.160 | 0.167 | 0.487 | 0.644 | 0.096 | **0.139** |
| rel. $D_R$ | 0.147 | 0.142 | 0.006 | 0.003 | 0.003 | 0.005 | 0.697 | 0.542 | 0.046 | 0.032 | 0.357 | 0.413 | 0.035 | 0.021 | 0.029 | 0.026 | 0.159 | 0.095 | 0.052 | 0.051 |
| $Ac_{RU}$ | 0.129 | 0.123 | 0.672 | 0.453 | 0.330 | 0.350 | 0.473 | 0.484 | 0.121 | 0.092 | 0.492 | 0.581 | 0.246 | 0.269 | 0.195 | 0.131 | 0.650 | 0.612 | 0.068 | 0.102 |
| $Ac_{RB}$ | 0.130 | 0.125 | 0.563 | 0.276 | 0.249 | 0.128 | 0.478 | 0.470 | 0.103 | 0.077 | 0.516 | 0.444 | 0.039 | 0.143 | 0.153 | 0.102 | 0.612 | 0.579 | 0.064 | 0.068 |
| $DAc_R$ | 0.137 | 0.136 | 0.721 | 0.426 | 0.393 | 0.471 | 0.405 | 0.476 | 0.121 | 0.110 | 0.403 | 0.499 | 0.279 | 0.207 | 0.212 | 0.173 | 0.480 | 0.518 | 0.067 | 0.105 |
| rel. $Ac_R$ | 0.145 | 0.138 | 0.180 | 0.027 | 0.023 | 0.021 | 0.368 | 0.327 | 0.065 | 0.050 | 0.287 | 0.297 | 0.006 | 0.044 | 0.046 | 0.043 | 0.436 | 0.402 | 0.059 | 0.063 |
| $P_{RU}$ | 0.001 | 0.002 | 0.088 | 0.100 | 0.120 | 0.287 | 0.404 | 0.101 | 0.052 | 0.074 | 0.483 | 0.596 | 0.167 | 0.115 | 0.020 | 0.114 | 0.479 | 0.108 | 0.068 | 0.076 |
| $P_{RB}$ | 0.001 | 0.002 | 0.070 | 0.080 | 0.101 | 0.256 | 0.400 | 0.101 | 0.058 | 0.083 | 0.412 | 0.595 | 0.177 | 0.059 | 0.092 | 0.114 | 0.536 | 0.108 | 0.054 | 0.078 |

| | | | | | | | | | | | | | | | | | | | | |
|---|---|---|---|---|---|---|---|---|---|---|---|---|---|---|---|---|---|---|---|---|
| $DP_R$ | 0.001 | 0.002 | 0.421 | 0.419 | 0.241 | 0.310 | 0.386 | 0.091 | 0.063 | 0.063 | 0.348 | 0.563 | 0.187 | 0.175 | 0.021 | 0.217 | 0.460 | 0.103 | 0.085 | 0.068 |
| rel. $P_R$ | 0.001 | 0.002 | 0.022 | 0.010 | 0.008 | 0.035 | 0.391 | 0.101 | 0.088 | 0.068 | 0.446 | 0.569 | 0.067 | 0.008 | 0.078 | 0.041 | 0.316 | 0.104 | 0.060 | 0.078 |
| $N_{RU}$ | 0.054 | 0.047 | 0.665 | 0.419 | 0.500 | 0.408 | 0.774 | 0.631 | 0.053 | 0.091 | 0.408 | 0.457 | 0.042 | 0.135 | 0.011 | 0.003 | 0.075 | 0.157 | 0.089 | 0.086 |
| $N_{RB}$ | 0.060 | 0.054 | 0.297 | 0.268 | 0.367 | 0.354 | 0.824 | 0.624 | 0.057 | 0.094 | 0.487 | 0.494 | 0.089 | 0.156 | 0.056 | 0.069 | 0.075 | 0.154 | 0.078 | 0.071 |
| $DN_R$ | 0.028 | 0.033 | 0.805 | 0.519 | 0.370 | 0.500 | 0.565 | 0.654 | 0.042 | 0.080 | 0.380 | 0.401 | 0.151 | 0.234 | 0.123 | 0.129 | 0.073 | 0.161 | 0.091 | 0.098 |
| rel. $N_R$ | 0.055 | 0.043 | 0.007 | 0.007 | 0.015 | 0.017 | 0.384 | 0.545 | 0.056 | 0.073 | 0.410 | 0.455 | 0.038 | 0.026 | 0.056 | 0.068 | 0.075 | 0.147 | 0.071 | 0.061 |
| $O_{RU}$ | 0.021 | 0.069 | 0.090 | 0.503 | 0.435 | 0.220 | 0.084 | 0.088 | 0.096 | 0.065 | 0.454 | 0.400 | 0.076 | 0.132 | 0.028 | 0.000 | 0.281 | 0.463 | 0.075 | 0.075 |
| $O_{RB}$ | 0.131 | 0.132 | 0.074 | 0.112 | 0.082 | 0.046 | 0.108 | 0.125 | 0.091 | 0.048 | 0.407 | 0.342 | 0.072 | 0.143 | 0.080 | 0.000 | 0.355 | 0.420 | 0.054 | 0.067 |
| $DO_R$ | 0.126 | 0.125 | 0.121 | 0.477 | 0.183 | 0.120 | 0.529 | 0.255 | 0.125 | 0.076 | 0.462 | 0.429 | 0.067 | 0.220 | 0.000 | 0.000 | 0.206 | 0.562 | 0.076 | 0.078 |
| rel. $O_R$ | 0.130 | 0.138 | 0.039 | 0.037 | 0.025 | 0.004 | 0.108 | 0.125 | 0.036 | 0.047 | 0.401 | 0.342 | 0.000 | 0.018 | 0.084 | 0.000 | 0.274 | 0.166 | 0.051 | 0.066 |
| $T_{LU}$ | 0.128 | 0.130 | **0.936** | **0.933** | **0.879** | **0.880** | 0.627 | 0.590 | 0.111 | 0.114 | 0.362 | 0.379 | 0.666 | 0.673 | **0.313** | **0.312** | 0.742 | 0.746 | 0.138 | 0.129 |
| $T_{LB}$ | 0.138 | 0.140 | 0.899 | 0.897 | 0.846 | 0.820 | 0.589 | 0.612 | 0.113 | 0.095 | 0.352 | 0.377 | 0.506 | 0.633 | 0.299 | 0.288 | 0.624 | 0.700 | 0.107 | 0.109 |
| $DT_L$ | 0.121 | 0.118 | 0.895 | 0.881 | 0.817 | 0.845 | 0.585 | 0.513 | 0.085 | 0.097 | 0.419 | 0.490 | 0.614 | 0.412 | 0.257 | 0.244 | 0.863 | 0.793 | **0.144** | 0.127 |
| rel. $T_L$ | 0.141 | 0.144 | 0.193 | 0.238 | 0.386 | 0.279 | 0.539 | 0.673 | 0.090 | 0.058 | 0.354 | 0.400 | 0.298 | 0.403 | 0.173 | 0.155 | 0.422 | 0.556 | 0.072 | 0.078 |
| $H_{LU}$ | 0.146 | 0.144 | 0.537 | 0.530 | 0.453 | 0.475 | 0.514 | 0.494 | 0.103 | 0.104 | 0.341 | 0.345 | 0.105 | 0.108 | 0.205 | 0.204 | 0.630 | 0.636 | 0.055 | 0.051 |
| $H_{LB}$ | 0.137 | 0.144 | 0.515 | 0.367 | 0.427 | 0.448 | 0.593 | 0.766 | 0.114 | 0.131 | 0.454 | 0.460 | 0.223 | 0.249 | 0.192 | 0.194 | 0.488 | 0.705 | 0.048 | 0.052 |
| $DH_L$ | 0.144 | 0.138 | 0.417 | 0.505 | 0.359 | 0.345 | 0.415 | 0.281 | 0.092 | 0.085 | 0.312 | 0.260 | 0.017 | 0.014 | 0.148 | 0.142 | 0.809 | 0.700 | 0.062 | 0.052 |
| rel. $H_L$ | 0.128 | 0.134 | 0.193 | 0.112 | 0.188 | 0.128 | 0.691 | **0.848** | 0.069 | 0.090 | **0.615** | 0.615 | 0.203 | 0.312 | 0.075 | 0.077 | 0.385 | 0.573 | 0.060 | 0.064 |
| $Ar_{LU}$ | 0.154 | 0.152 | 0.332 | 0.303 | 0.556 | 0.602 | 0.406 | 0.375 | 0.143 | 0.139 | 0.428 | 0.429 | 0.109 | 0.121 | 0.274 | 0.268 | 0.082 | 0.083 | 0.039 | 0.040 |
| $Ar_{LB}$ | 0.145 | 0.146 | 0.363 | 0.305 | 0.535 | 0.506 | 0.480 | 0.532 | 0.100 | 0.098 | 0.392 | 0.440 | 0.244 | 0.307 | 0.246 | 0.230 | 0.163 | 0.177 | 0.040 | 0.042 |
| $DAr_L$ | 0.159 | 0.155 | 0.273 | 0.248 | 0.361 | 0.462 | 0.301 | 0.253 | 0.149 | 0.135 | 0.367 | 0.329 | 0.023 | 0.010 | 0.216 | 0.207 | 0.090 | 0.073 | 0.046 | 0.045 |
| rel. $Ar_L$ | 0.128 | 0.134 | 0.144 | 0.085 | 0.170 | 0.103 | 0.424 | 0.588 | 0.035 | 0.045 | 0.464 | 0.457 | 0.206 | 0.282 | 0.066 | 0.063 | 0.422 | 0.555 | 0.068 | 0.070 |
| $D_{LU}$ | 0.157 | **0.159** | 0.134 | 0.135 | 0.105 | 0.109 | 0.475 | 0.498 | 0.038 | 0.039 | 0.365 | 0.375 | 0.322 | 0.312 | 0.085 | 0.086 | 0.506 | 0.484 | 0.054 | 0.055 |
| $D_{LB}$ | 0.146 | 0.137 | 0.184 | 0.225 | 0.153 | 0.153 | 0.554 | 0.697 | 0.047 | 0.050 | 0.373 | 0.360 | 0.201 | 0.160 | 0.105 | 0.111 | 0.554 | 0.508 | 0.058 | 0.066 |
| $DD_L$ | 0.150 | 0.154 | 0.117 | 0.104 | 0.092 | 0.095 | 0.452 | 0.413 | 0.039 | 0.041 | 0.314 | 0.333 | 0.327 | 0.339 | 0.080 | 0.078 | 0.434 | 0.461 | 0.054 | 0.055 |
| rel. $D_L$ | 0.124 | 0.121 | 0.107 | 0.120 | 0.145 | 0.094 | 0.506 | 0.612 | 0.059 | 0.061 | 0.392 | 0.388 | 0.024 | 0.011 | 0.085 | 0.088 | 0.302 | 0.286 | 0.070 | 0.071 |
| $Ac_{LU}$ | 0.159 | 0.158 | 0.581 | 0.569 | 0.387 | 0.408 | 0.621 | 0.645 | 0.035 | 0.036 | 0.445 | 0.445 | 0.077 | 0.086 | 0.191 | 0.191 | 0.500 | 0.505 | 0.032 | 0.030 |
| $Ac_{LB}$ | 0.132 | 0.139 | 0.635 | 0.591 | 0.402 | 0.466 | 0.533 | 0.615 | 0.072 | 0.047 | 0.470 | 0.430 | 0.130 | 0.136 | 0.193 | 0.176 | 0.539 | 0.624 | 0.048 | 0.053 |
| $DAc_L$ | **0.162** | 0.156 | 0.289 | 0.272 | 0.218 | 0.198 | 0.619 | 0.511 | 0.032 | 0.039 | 0.454 | 0.510 | 0.029 | 0.026 | 0.133 | 0.121 | 0.412 | 0.332 | 0.036 | 0.028 |
| rel. $Ac_L$ | 0.137 | 0.138 | 0.218 | 0.153 | 0.135 | 0.172 | 0.337 | 0.401 | 0.079 | 0.052 | 0.419 | 0.481 | 0.059 | 0.049 | 0.098 | 0.077 | 0.393 | 0.410 | 0.061 | 0.054 |

| | | | | | | | | | | | | | | | | | | | | |
|---|---|---|---|---|---|---|---|---|---|---|---|---|---|---|---|---|---|---|---|---|
| $P_{LU}$ | 0.112 | 0.112 | 0.210 | 0.212 | 0.168 | 0.151 | 0.140 | 0.139 | 0.035 | 0.035 | 0.226 | 0.228 | 0.085 | 0.103 | 0.091 | 0.091 | 0.298 | 0.277 | 0.043 | 0.045 |
| $P_{LB}$ | 0.061 | 0.074 | 0.162 | 0.152 | 0.110 | 0.142 | 0.076 | 0.098 | 0.040 | 0.039 | 0.260 | 0.302 | 0.032 | 0.025 | 0.079 | 0.090 | 0.296 | 0.162 | 0.044 | 0.054 |
| $DP_{L}$ | 0.107 | 0.106 | 0.162 | 0.178 | 0.151 | 0.104 | 0.142 | 0.138 | 0.034 | 0.034 | 0.233 | 0.228 | 0.096 | 0.118 | 0.077 | 0.059 | 0.220 | 0.283 | 0.046 | 0.044 |
| rel. $P_{L}$ | 0.063 | 0.076 | 0.254 | 0.210 | 0.132 | 0.161 | 0.076 | 0.098 | 0.040 | 0.039 | 0.260 | 0.294 | 0.033 | 0.024 | 0.088 | 0.092 | 0.348 | 0.150 | 0.042 | 0.055 |
| $N_{LU}$ | 0.074 | 0.074 | 0.072 | 0.071 | 0.261 | 0.264 | 0.104 | 0.104 | 0.027 | 0.027 | 0.373 | 0.445 | 0.034 | 0.033 | 0.192 | 0.184 | 0.716 | 0.714 | 0.050 | 0.049 |
| $N_{LB}$ | 0.069 | 0.068 | 0.076 | 0.078 | 0.292 | 0.282 | 0.105 | 0.106 | 0.027 | 0.025 | 0.343 | 0.405 | 0.051 | 0.044 | 0.189 | 0.184 | 0.711 | 0.705 | 0.051 | 0.060 |
| $DN_{L}$ | 0.072 | 0.072 | 0.074 | 0.071 | 0.271 | 0.284 | 0.099 | 0.101 | 0.024 | 0.024 | 0.431 | 0.435 | 0.025 | 0.030 | 0.196 | 0.201 | 0.709 | 0.694 | 0.051 | 0.046 |
| rel. $N_{L}$ | 0.069 | 0.068 | 0.078 | 0.079 | 0.300 | 0.283 | 0.105 | 0.106 | 0.027 | 0.026 | 0.393 | 0.445 | 0.053 | 0.046 | 0.190 | 0.188 | 0.710 | 0.705 | 0.051 | 0.061 |
| $O_{LU}$ | 0.114 | 0.115 | 0.878 | 0.873 | 0.706 | 0.710 | 0.475 | 0.453 | 0.076 | 0.080 | 0.377 | 0.381 | 0.612 | 0.614 | 0.157 | 0.157 | 0.519 | 0.521 | 0.136 | 0.133 |
| $O_{LB}$ | 0.134 | 0.129 | 0.668 | 0.741 | 0.649 | 0.599 | 0.573 | 0.548 | 0.096 | 0.075 | 0.381 | 0.402 | 0.522 | 0.620 | 0.168 | 0.166 | 0.590 | 0.581 | 0.127 | 0.122 |
| $DO_{L}$ | 0.108 | 0.113 | 0.823 | 0.805 | 0.569 | 0.619 | 0.392 | 0.403 | 0.057 | 0.072 | 0.313 | 0.310 | 0.484 | 0.368 | 0.106 | 0.117 | 0.422 | 0.462 | 0.131 | 0.129 |
| rel. $O_{L}$ | 0.146 | 0.149 | 0.085 | 0.123 | 0.216 | 0.138 | 0.505 | 0.575 | 0.089 | 0.060 | 0.372 | 0.395 | 0.210 | 0.249 | 0.130 | 0.128 | 0.510 | 0.517 | 0.068 | 0.069 |
| $DT_{RL}$ | 0.121 | 0.123 | 0.918 | 0.911 | 0.853 | 0.862 | 0.643 | 0.578 | 0.106 | 0.108 | 0.391 | 0.426 | 0.563 | 0.438 | 0.282 | 0.274 | 0.813 | 0.753 | 0.140 | 0.133 |
| rel. $T_{RL}$ | 0.141 | 0.144 | 0.028 | 0.040 | 0.129 | 0.095 | 0.466 | 0.591 | 0.059 | 0.042 | 0.364 | 0.414 | 0.185 | 0.294 | 0.085 | 0.098 | 0.296 | 0.471 | 0.051 | 0.055 |
| $DH_{RL}$ | 0.142 | 0.139 | 0.848 | 0.813 | 0.684 | 0.741 | 0.675 | 0.468 | 0.137 | 0.113 | 0.412 | 0.340 | 0.124 | 0.149 | 0.271 | 0.224 | **0.896** | **0.813** | 0.099 | 0.085 |
| rel. $H_{RL}$ | 0.131 | 0.137 | 0.015 | 0.020 | 0.060 | 0.016 | 0.395 | 0.601 | 0.036 | 0.050 | 0.543 | 0.608 | 0.042 | 0.144 | 0.041 | 0.079 | 0.131 | 0.468 | 0.053 | 0.057 |
| $DAr_{RL}$ | 0.157 | 0.153 | 0.518 | 0.562 | 0.630 | 0.604 | 0.550 | 0.423 | **0.151** | 0.132 | 0.358 | 0.348 | 0.091 | 0.047 | 0.245 | 0.227 | 0.142 | 0.186 | 0.053 | 0.057 |
| rel. $Ar_{RL}$ | 0.127 | 0.132 | 0.017 | 0.034 | 0.038 | 0.059 | 0.289 | 0.390 | 0.028 | 0.027 | 0.445 | 0.403 | 0.090 | 0.082 | 0.044 | 0.033 | 0.280 | 0.384 | 0.064 | 0.070 |
| $DD_{RL}$ | 0.150 | 0.155 | 0.395 | 0.506 | 0.380 | 0.236 | 0.445 | 0.420 | 0.042 | 0.027 | 0.345 | 0.360 | 0.434 | 0.454 | 0.109 | 0.122 | 0.478 | 0.575 | 0.061 | 0.076 |
| rel. $D_{RL}$ | 0.142 | 0.138 | 0.009 | 0.003 | 0.005 | 0.014 | 0.643 | 0.644 | 0.068 | 0.047 | 0.382 | 0.403 | 0.005 | 0.008 | 0.037 | 0.031 | 0.259 | 0.167 | 0.054 | 0.056 |
| $DAc_{RL}$ | 0.161 | 0.156 | 0.441 | 0.421 | 0.325 | 0.248 | 0.588 | 0.534 | 0.043 | 0.047 | 0.450 | 0.524 | 0.055 | 0.063 | 0.139 | 0.139 | 0.432 | 0.376 | 0.038 | 0.036 |
| rel. $Ac_{RL}$ | 0.133 | 0.138 | 0.287 | 0.125 | 0.094 | 0.248 | 0.380 | 0.465 | 0.071 | 0.040 | 0.416 | 0.471 | 0.061 | 0.064 | 0.097 | 0.077 | 0.489 | 0.444 | 0.069 | 0.061 |
| $DP_{RL}$ | 0.108 | 0.107 | 0.423 | 0.420 | 0.251 | 0.310 | 0.401 | 0.154 | 0.062 | 0.063 | 0.342 | 0.463 | 0.188 | 0.176 | 0.070 | 0.218 | 0.461 | 0.321 | 0.083 | 0.045 |
| rel. $P_{RL}$ | 0.063 | 0.077 | 0.022 | 0.012 | 0.008 | 0.034 | 0.388 | 0.126 | 0.092 | 0.068 | 0.443 | 0.502 | 0.067 | 0.008 | 0.076 | 0.040 | 0.303 | 0.173 | 0.060 | 0.070 |
| $DN_{RL}$ | 0.084 | 0.087 | 0.673 | 0.384 | 0.467 | 0.479 | 0.476 | 0.546 | 0.025 | 0.035 | 0.422 | 0.418 | 0.091 | 0.160 | 0.230 | 0.242 | 0.719 | 0.713 | 0.065 | 0.063 |
| rel. $N_{RL}$ | 0.069 | 0.051 | 0.006 | 0.005 | 0.006 | 0.012 | 0.388 | 0.551 | 0.055 | 0.073 | 0.422 | 0.479 | 0.041 | 0.031 | 0.077 | 0.066 | 0.709 | 0.581 | 0.071 | 0.065 |
| $DO_{RL}$ | 0.108 | 0.113 | 0.822 | 0.807 | 0.577 | 0.619 | 0.393 | 0.403 | 0.059 | 0.072 | 0.313 | 0.310 | 0.484 | 0.382 | 0.106 | 0.117 | 0.426 | 0.475 | 0.132 | 0.129 |
| rel. $O_{RL}$ | 0.146 | 0.148 | 0.084 | 0.121 | 0.210 | 0.127 | 0.504 | 0.576 | 0.087 | 0.058 | 0.372 | 0.396 | 0.210 | 0.254 | 0.130 | 0.128 | 0.505 | 0.501 | 0.068 | 0.070 |

**Table S5.** Enrichment Factors at 1% subsetting for protein structures of ten PPI targets obtained using post docking derivatized SASA descriptors. The best enrichment score corresponding to each target protein (across columns) is shown in bold and highlighted in green.

| SASA descriptor | BAZ2B | | BCL2 | | BCLXL | | BRD4-1 | | CREBBP | | HIV-IN | | XIAP | | MCL1 | | MDM2 | | Menin | |
|---|---|---|---|---|---|---|---|---|---|---|---|---|---|---|---|---|---|---|---|---|
| | 4XUA | 5E73 | 2O21 | 4LVT | 3INQ | 3WIZ | 5D3L | 5KU3 | 5EIC | 5MMG | 4CFD | 4CHO | 1TFT | 5C3H | 5FC4 | 5MES | 4ODF | 4ZFI | 5DB2 | 6B41 |
| Surflex | 0.77 | 0.86 | 25 | **28** | 26 | 27 | 0.96 | 0.72 | 1.8 | **5.1** | 1.4 | 1.5 | **42** | **33** | 6.1 | 3.3 | **3** | 2.5 | 3.4 | 1 |
| $T_{RU}$ | 0.96 | 1.2 | **28** | 27 | 44 | 45 | 1.8 | 1.6 | 1.4 | 1.5 | 1.5 | 1.6 | 16 | 16 | 11 | 8.9 | 2.5 | 2.6 | 3.3 | 3.3 |
| $T_{RB}$ | 0.98 | 1.2 | 23 | 18 | 29 | 28 | **1.9** | 0.96 | 1.3 | 1.4 | 0.91 | 1.3 | 7.8 | 9.1 | 4 | 4.4 | 1.8 | 2.3 | 3 | 2 |
| $\Delta T_R$ | 1.1 | 1.1 | **28** | 27 | **47** | 46 | 1.3 | 1.6 | 1.5 | 1.4 | 1.7 | 1.3 | 19 | 20 | 16 | 14 | 2.5 | 2.6 | 3.1 | 4.1 |
| rel. $T_R$ | 1.1 | 1.3 | 0 | 0.057 | 0 | 0.1 | 0.48 | 0.12 | 0.15 | 0.077 | 0.57 | 0.46 | 0.18 | 0 | 0.91 | 0.63 | 0.13 | 0.36 | 0.71 | 0.85 |
| $H_{RU}$ | 0.96 | 1.1 | 27 | 27 | 41 | 41 | 1.7 | 1.4 | 1.2 | 1.7 | 1.4 | 1.5 | 9.1 | 13 | 11 | 7 | 2.5 | 2.4 | 3 | 3 |
| $H_{RB}$ | 0.93 | 1.1 | 16 | 11 | 19 | 22 | 1.1 | 0.6 | 1 | 2.1 | 1.4 | 0.91 | 2.9 | 7.8 | 2.4 | 3.9 | 0.89 | 1.7 | 1.8 | 2.8 |
| $\Delta H_R$ | 1.2 | 1.1 | **28** | 27 | 44 | 45 | 1.7 | 1.4 | 1.5 | 1.2 | 1.7 | 1.4 | 15 | 19 | 14 | 8.9 | 2.5 | 2.7 | 2.8 | 3.3 |
| rel. $H_R$ | 0.92 | 0.99 | 0 | 0.11 | 0.42 | 0.1 | 0 | 0.12 | 0.54 | 0.15 | 0.69 | 0.34 | 0 | 0.088 | 0.77 | 1.4 | 0 | 0.94 | 0.71 | 1 |
| $Ar_{RU}$ | 0.86 | 0.93 | 11 | 22 | 6.7 | 26 | 1.2 | 1.6 | 1.6 | 0.77 | 0.57 | 1.1 | 2.6 | 4.4 | 4.6 | 1.4 | 2.3 | 1.8 | 1.8 | 1.8 |
| $Ar_{RB}$ | 0.9 | 1 | 0.28 | 0.62 | 0.1 | 6.9 | 0.12 | 0.6 | 1.5 | 0.62 | 0.46 | 1 | 2.1 | 0.35 | 3.1 | 1.3 | 1.1 | 0.54 | 0.71 | 1.7 |
| $\Delta Ar_R$ | 0.99 | 1.1 | 22 | 26 | 27 | 22 | 1.7 | 1.2 | 0.62 | 1.4 | 1 | 1.4 | 6.7 | 8.3 | 7.7 | 5.1 | 2.4 | 2.5 | 2.3 | 2.3 |
| rel. $Ar_R$ | 0.92 | 1.1 | 0.057 | 0.057 | 0.1 | 0.42 | 0 | 0.12 | 0.23 | 0.46 | 0.34 | 0.57 | 0.18 | 0 | 0.77 | 0.35 | 0.13 | 0.22 | 0.43 | 0.85 |
| $D_{RU}$ | 1 | 1.1 | 20 | 19 | 23 | 22 | 1.8 | 0.96 | 1.3 | 1.7 | 1 | 1.6 | 7.6 | 4.1 | 5.6 | 6 | 2 | 1.9 | 2.1 | 2.4 |
| $D_{RB}$ | 1.2 | 1.2 | 13 | 1.8 | 1.2 | 5.3 | 1.6 | 1.1 | 1.2 | 0.92 | 0.23 | 1.4 | 1.1 | 3.2 | 1.3 | 1.7 | 1.4 | 0.98 | 0.71 | 1.3 |
| $\Delta D_R$ | 0.88 | 0.99 | 14 | 22 | 21 | 22 | 1.1 | 1.3 | 1.1 | 2.5 | 1.3 | 1.4 | 11 | 6.3 | 5.6 | 5.1 | 2.4 | 2.5 | 2.8 | **4.6** |
| rel. $D_R$ | 1.2 | 1.2 | 0.057 | 0 | 0.1 | 0 | 1.3 | 1.1 | 0.69 | 0.23 | 0.57 | 0.46 | 0.18 | 0.088 | 0.21 | 0 | 0.22 | 0.18 | 1.3 | 1.6 |
| $Ac_{RU}$ | 0.88 | 0.79 | 25 | 22 | 23 | 7.4 | 1.2 | 0.84 | 1.6 | 1.1 | 1.1 | 1.4 | 7.7 | 11 | 7.8 | 4.3 | 2.1 | 2.5 | 1.8 | 3.1 |
| $Ac_{RB}$ | 0.9 | 0.77 | 21 | 11 | 14 | 4.1 | 1.4 | 0.84 | 1.4 | 0.77 | 1.4 | 0.69 | 2.6 | 5 | 5.1 | 2.6 | 2.1 | 2.1 | 1.3 | 1.7 |
| $\Delta Ac_R$ | 0.83 | 1 | 26 | 14 | 20 | 21 | 0.84 | 1.1 | 1.9 | 1.8 | 1.4 | 1 | 8.6 | 7.2 | 7.5 | 6.4 | 2.2 | 2.3 | 1.7 | 3.8 |
| rel. $Ac_R$ | 1.3 | 1.2 | 2 | 0.057 | 0 | 0.1 | 0.12 | 0.6 | 0.62 | 0.38 | 0.11 | 0.8 | 0.088 | 0.79 | 0.14 | 0.56 | 0.63 | 0.98 | 0.71 | 1 |
| $P_{RU}$ | 0.044 | 0.073 | 5.7 | 5.9 | 6.8 | 20 | 0.72 | 0.48 | 1.1 | 0.69 | 1.4 | 1.7 | 3.4 | 2.1 | 1.5 | 0.14 | 1.6 | 1.6 | 2 | 1 |
| $P_{RB}$ | 0.044 | 0.073 | 2 | 0.62 | 4.5 | 12 | 0.6 | 0.48 | 1.1 | 0.92 | 1.1 | 1.6 | 3.8 | 0.88 | 2.3 | 1.2 | 2.1 | 1.4 | 1 | 1.1 |

| | | | | | | | | | | | | | | | | | | | | |
|---|---|---|---|---|---|---|---|---|---|---|---|---|---|---|---|---|---|---|---|---|
| $\Delta P_R$ | 0.029 | 0.073 | 13 | 13 | 10 | 12 | 1.2 | 0.36 | 0.85 | 1.3 | 1.4 | 1.6 | 11 | 6.3 | 1.2 | 7.5 | 2.2 | 1.6 | 2.1 | 1.1 |
| rel. $P_R$ | 0.044 | 0.073 | 0.057 | 0.23 | 0 | 0 | 0.84 | 0.48 | 0.54 | 1 | 1.1 | 1.4 | 0.18 | 0 | 1.3 | 0 | 0.8 | 1.1 | 0.57 | 1.4 |
| $N_{RU}$ | 0.54 | 0.77 | 15 | 9.3 | 19 | 19 | 1.7 | 0.84 | 0.77 | 1.1 | 1.1 | 1.3 | 3.4 | 7.1 | 0 | 0.21 | 1.2 | 1.3 | 2.4 | 2.8 |
| $N_{RB}$ | 0.54 | 0.77 | 3.8 | 3.4 | 13 | 15 | 1.7 | 0.96 | 0.77 | 1.8 | 0.69 | 1.4 | 4.6 | 4.9 | 0.84 | 0.77 | 1.2 | 1.1 | 2.3 | 1.3 |
| $\Delta N_R$ | 0.95 | 0.67 | **28** | 18 | 20 | 25 | 1.1 | 1.3 | 0.62 | 1.6 | 1.6 | 0.8 | 8 | 9.8 | 3.6 | 2.7 | 1.1 | 1.4 | 2.1 | 3.3 |
| rel. $N_R$ | 0 | 0 | 0 | 0.17 | 0.21 | 0 | 0 | 0.72 | 0.46 | 0.54 | 0.91 | 0.69 | 0.62 | 0 | 0.84 | 0.63 | 1.2 | 0.72 | 1 | 1.3 |
| $O_{RU}$ | 0 | 0.015 | 6.6 | 25 | 22 | 1.9 | 0.12 | 0.12 | 1.2 | 1.2 | 1.3 | 0.8 | 4.9 | 2.5 | 0 | 0 | 0.67 | 1.7 | 1.7 | 1.6 |
| $O_{RB}$ | 0.8 | 0.98 | 2.8 | 2.3 | 1.9 | 1.8 | 0.24 | 0.12 | 0.77 | 0.31 | 1 | 1 | 4.5 | 0.79 | 0.99 | 0 | 0.8 | 1.4 | 1.7 | 1.3 |
| $\Delta O_R$ | 0.86 | 0.88 | 2.8 | 21 | 4.9 | 3.6 | 0.36 | 0.24 | 2.2 | 0.85 | 1.3 | 0.8 | 3.4 | 7.4 | 0 | 0 | 1.5 | 2.6 | 2.1 | 1.3 |
| rel. $O_R$ | 0.77 | 0.99 | 0 | 0 | 0 | 0 | 0.24 | 0.12 | 0.46 | 0.38 | 0.8 | 0.91 | 0 | 0 | 1.9 | 0 | 0 | 0.045 | 1 | 1 |
| $T_{LU}$ | 1.1 | 1.1 | 27 | 27 | 46 | 46 | 1.1 | 0.96 | 1.9 | 1.9 | **2.4** | **2.4** | 29 | 30 | **17** | **17** | 2.6 | 2.6 | 4.8 | 3.7 |
| $T_{LB}$ | 1.2 | 1.3 | 27 | 27 | 45 | 46 | 0.84 | 1.1 | 1.8 | 1.2 | **2.4** | **2.4** | 25 | 29 | 14 | 13 | 2.6 | 2.6 | 2.7 | 2.8 |
| $\Delta T_L$ | 1 | 0.88 | 27 | **28** | 46 | **47** | 1.2 | 0.84 | 0.85 | 1.8 | 1.5 | 1.1 | 25 | 18 | 13 | 10 | 2.7 | 2.7 | **5** | 3.8 |
| rel. $T_L$ | 1.2 | 1.1 | 5.3 | 4.5 | 15 | 12 | 1.2 | 1.6 | 1.6 | 0.85 | 2.3 | 2.3 | 17 | 19 | 4.6 | 3.1 | 2.4 | 2.6 | 1.7 | 1.3 |
| $H_{LU}$ | 1.1 | 1.1 | 17 | 17 | 23 | 25 | 1.1 | 1.1 | 1.5 | 1.2 | 1.9 | 2.1 | 2.7 | 3.2 | 6.6 | 6.2 | 1.5 | 1.6 | 0.85 | 0.71 |
| $H_{LB}$ | 1 | 0.98 | 17 | 13 | 20 | 23 | 1.4 | 1.7 | 1.5 | 2.5 | 2.1 | 2.2 | 10 | 10 | 6.8 | 6.6 | 2.1 | 2.5 | 1 | 0.85 |
| $\Delta H_L$ | 0.99 | 1 | 14 | 16 | 15 | 14 | 0.72 | 0.6 | 1 | 0.62 | 0.23 | 0 | 0.088 | 0 | 2.9 | 3.9 | 2.3 | 1.9 | 0.43 | 0.85 |
| rel. $H_L$ | 0.8 | 0.9 | 3.6 | 2.1 | 4.7 | 3.1 | **1.9** | **2.2** | 0.77 | 2.5 | 1.9 | 1.9 | 4.9 | 11 | 0.7 | 0.99 | 1.7 | 2.1 | 1.4 | 0.43 |
| $Ar_{LU}$ | 1.2 | 1.2 | 9 | 8.5 | 24 | 25 | 0.72 | 0.6 | **3.2** | 3.5 | 1.7 | 1.6 | 4 | 4.1 | 9.6 | 9.1 | 0.13 | 0.22 | 1 | 1 |
| $Ar_{LB}$ | 1.2 | 1.2 | 11 | 8.7 | 28 | 23 | 0.6 | 1.2 | 1.4 | 1.8 | 2.2 | 2.2 | 11 | 14 | 7.6 | 6.8 | 0.85 | 1 | 0.43 | 0.28 |
| $\Delta Ar_L$ | 1.4 | 1.3 | 8.8 | 7.5 | 14 | 24 | 0.36 | 0.24 | 3 | 2.4 | 1.1 | 0.8 | 0.18 | 0.26 | 5.5 | 7.2 | 0.18 | 0.13 | 0.71 | 0.71 |
| rel. $Ar_L$ | 0.93 | 1.1 | 2.8 | 0.45 | 3.6 | 1.5 | 0.6 | 1.2 | 0.54 | 0.46 | 0.8 | 1.5 | 5.6 | 6.3 | 0.56 | 0.63 | 1.7 | 2.1 | 1.6 | 1.3 |
| $D_{LU}$ | **1.8** | **1.8** | 1.5 | 1.6 | 4.4 | 4.4 | 0.84 | 0.84 | 0.54 | 0.62 | 1.7 | 1.8 | 7.1 | 6.9 | 1.6 | 1.6 | 2.5 | 2.6 | 1.6 | 1.7 |
| $D_{LB}$ | 1.4 | 1.2 | 2.9 | 4 | 5 | 5 | 1.3 | 1.4 | 0.38 | 0.92 | 1.8 | 2.1 | 6.4 | 4.8 | 1.2 | 1.8 | 2.5 | 2.5 | 1.1 | 1.4 |
| $\Delta D_L$ | 1.4 | 1.4 | 1.1 | 1.5 | 4.4 | 4.3 | 0.6 | 0.84 | 0.46 | 0.54 | 0.57 | 0.57 | 5.8 | 5.6 | 0.84 | 0.99 | 1.9 | 2.1 | 1.6 | 1.7 |
| rel. $D_L$ | 1.1 | 0.12 | 0 | 0.057 | 0.1 | 0 | 1.3 | 1.2 | 0.77 | 1.2 | 0.57 | 0.8 | 0.18 | 0.088 | 0.14 | 0 | 0.045 | 0.089 | 1.3 | 1.3 |
| $Ac_{LU}$ | 1.7 | 1.5 | 15 | 15 | 14 | 16 | 1.4 | 1.6 | 0.31 | 0.38 | **2.4** | **2.4** | 1.9 | 2.4 | 6.8 | 7.7 | 2.1 | 2 | 0.43 | 0.28 |
| $Ac_{LB}$ | 0.99 | 0.96 | 22 | 21 | 20 | 24 | 1.6 | 1.6 | 0.69 | 0.54 | **2.4** | **2.4** | 4.8 | 5.2 | 7.2 | 6.1 | 2.3 | 2.4 | 0.85 | 0.71 |
| $\Delta Ac_L$ | **1.8** | 1.6 | 7 | 6.6 | 6.7 | 5.5 | 1.6 | 0.96 | 0.38 | 0.38 | 2.1 | 2.2 | 0.26 | 0.44 | 3.8 | 3.9 | 0.94 | 0.94 | 0.71 | 0.28 |
| rel. $Ac_L$ | 1.2 | 1.2 | 1.5 | 0.68 | 1.1 | 0.83 | 0.24 | 0.84 | 0.62 | 0.69 | 0.91 | 1.3 | 0.088 | 0 | 1.6 | 1.5 | 0.98 | 1.2 | 1.4 | 0.28 |

| | | | | | | | | | | | | | | | | | | | | |
|---|---|---|---|---|---|---|---|---|---|---|---|---|---|---|---|---|---|---|---|---|
| $P_{LU}$ | 1 | 1 | 0.68 | 0.62 | 3.9 | 4.2 | 0.96 | 0.84 | 0.38 | 0.38 | 0.34 | 0.46 | 0.88 | 0.71 | 1.5 | 1.5 | 1.4 | 1.2 | 0.71 | 0.57 |
| $P_{LB}$ | 1.1 | 0.92 | 0.51 | 0.85 | 4.5 | 4.7 | 0.24 | 0.48 | 0.46 | 0.38 | 0.34 | 0.69 | 0.71 | 0.88 | 1.9 | 1.8 | 1.3 | 0.76 | 0.57 | 0.43 |
| $\Delta P_L$ | 0.98 | 1.1 | 1.3 | 1.1 | 3.2 | 3.2 | 0.96 | 0.84 | 0.23 | 0.23 | 0.46 | 0.46 | 0.97 | 0.53 | 1.7 | 1.7 | 0.98 | 1.3 | 0.57 | 0.71 |
| rel. $P_L$ | 1.3 | 1.4 | 0 | 0 | 0 | 0 | 0.24 | 0.48 | 0.46 | 0.38 | 0.34 | 0.46 | 0 | 0.44 | 0 | 0 | 0 | 0 | 0.28 | 1 |
| $N_{LU}$ | 1.2 | 1.2 | 1.6 | 1.7 | 6.4 | 6.9 | 0.6 | 0.48 | 0.15 | 0.23 | 0.8 | 0.91 | 0.53 | 0.35 | 4.1 | 3.7 | 2.7 | 2.8 | 0.85 | 0.57 |
| $N_{LB}$ | 1 | 1 | 3.2 | 2.3 | 8.4 | 7.6 | 0.6 | 0.48 | 0.46 | 0.23 | 0.34 | 0.57 | 3.5 | 1.6 | 4.8 | 4.3 | 2.7 | **3** | 0.85 | 1.3 |
| $\Delta N_L$ | 1.3 | 1.1 | 2.6 | 1.4 | 6.3 | 6.3 | 0.36 | 0.36 | 0.23 | 0.31 | 0.91 | 0.91 | 0.088 | 0.35 | 3.6 | 5.9 | 2.6 | 2.7 | 0.57 | 0.43 |
| rel. $N_L$ | 0.99 | 0.99 | 3.5 | 2.7 | 11 | 6.4 | 0.6 | 0.48 | 0.54 | 0.31 | 1.7 | 2.3 | 4.3 | 2.4 | 5 | 5.1 | 2.7 | 2.9 | 0.85 | 1.4 |
| $O_{LU}$ | 0.9 | 1.1 | 26 | 26 | 40 | 40 | 0.72 | 0.72 | 1.8 | 2 | 1.7 | 0.91 | 26 | 26 | 7.2 | 7.2 | 2.4 | 2.4 | 3.7 | 3.6 |
| $O_{LB}$ | 1.1 | 1.1 | 23 | 25 | 34 | 33 | 0.96 | 1.1 | 1.9 | 1.3 | 2.1 | 1.9 | 24 | 26 | 7.2 | 6.5 | 2.5 | 2.5 | 3.4 | 3 |
| $\Delta O_L$ | 0.83 | 0.89 | 27 | 27 | 29 | 32 | 0.72 | 0.6 | 1.2 | 1.8 | 0.23 | 0.23 | 18 | 13 | 3 | 3.9 | 1.1 | 1.4 | 3.6 | 3.8 |
| rel. $O_L$ | 1.2 | 1.2 | 1.5 | 1.6 | 6.3 | 4.9 | 0.24 | 1.4 | 1.5 | 0.92 | 2.2 | 2.1 | 11 | 8.7 | 2.8 | 2.3 | 2.2 | 2.2 | 1 | 0.85 |
| $\Delta T_{RL}$ | 1 | 0.92 | 27 | 27 | **47** | **47** | 1.3 | 1.2 | 1.5 | 1.8 | 1.5 | 1.1 | 23 | 21 | 16 | 13 | 2.7 | 2.7 | 4.1 | 4 |
| rel. $T_{RL}$ | 1.2 | 1.3 | 0.57 | 0.85 | 5.5 | 4.2 | 0.96 | 1.3 | 0.85 | 0.38 | 2.2 | 2.1 | 11 | 14 | 1.7 | 1.3 | 1.7 | 2.3 | 1.1 | 0.85 |
| $\Delta H_{RL}$ | 1.2 | 1.1 | **28** | **28** | 38 | 40 | 1.8 | 1.2 | 1.3 | 1.1 | 1.1 | 1 | 3.5 | 6 | 11 | 8.3 | 2.5 | 2.7 | 2 | 1.8 |
| rel. $H_{RL}$ | 1.1 | 1.1 | 0.057 | 0.17 | 0.52 | 0.1 | 0.84 | 1.3 | 0.38 | 0.15 | 1.8 | 1.9 | 0.88 | 2.8 | 0.28 | 1.3 | 0.49 | 1.8 | 0.85 | 0.71 |
| $\Delta Ar_{RL}$ | 1.4 | 1.4 | 16 | 17 | 30 | 31 | 1.2 | 0.96 | 3.1 | 2.2 | 0.8 | 0.69 | 2.6 | 1.7 | 7.4 | 7.3 | 0.36 | 0.85 | 0.71 | 0.71 |
| rel. $Ar_{RL}$ | 1 | 1 | 0 | 0.28 | 0.83 | 0.73 | 0.12 | 0.84 | 0.23 | 0.077 | 1 | 0.91 | 1.8 | 1.4 | 0.42 | 0.56 | 0.8 | 1.2 | 1.3 | 0.85 |
| $\Delta D_{RL}$ | 1.4 | 1.5 | 5.8 | 9.1 | 10 | 6 | 0.72 | 0.96 | 0.54 | 0.077 | 1 | 1 | 12 | 12 | 1.8 | 2.2 | 2.6 | 2.5 | 1.6 | 2.6 |
| rel. $D_{RL}$ | 1 | 1.1 | 0 | 0 | 0 | 0.1 | 1.2 | 1.4 | 0.77 | 0.54 | 0.57 | 1.3 | 0.088 | 0 | 0.49 | 0 | 0.36 | 0.13 | 1.3 | 1.1 |
| $\Delta Ac_{RL}$ | 1.7 | 1.6 | 13 | 13 | 14 | 8.7 | 1.6 | 1.3 | 0.15 | 0.38 | 2.2 | 2.2 | 0.79 | 0.97 | 4.4 | 4.2 | 1.5 | 1.4 | 0.71 | 0.43 |
| rel. $Ac_{RL}$ | 0.93 | 1 | 4.9 | 1.5 | 0.52 | 8.7 | 0.48 | 0.84 | 0.38 | 0.38 | 1 | 2.1 | 1.1 | 1.1 | 0.91 | 0.77 | 1.3 | 1.3 | 1.7 | 0.85 |
| $\Delta P_{RL}$ | 0.98 | 1.1 | 13 | 13 | 10 | 12 | 1.2 | 0.84 | 0.85 | 1.3 | 1.3 | 1.3 | 11 | 6.4 | 2.3 | 7.3 | 2.2 | 1.7 | 2.1 | 1 |
| rel. $P_{RL}$ | 1.3 | 1.3 | 0.057 | 0.4 | 0 | 0 | 0.72 | 0.84 | 0.54 | 1 | 1.1 | 0.69 | 0.088 | 0 | 1.1 | 0 | 0 | 0 | 0.71 | 1 |
| $\Delta N_{RL}$ | 1.3 | 1.1 | 15 | 6.1 | 17 | 19 | 0.36 | 0.36 | 0.23 | 0.31 | 0.69 | 1 | 0.44 | 3 | 3.7 | 5.8 | 2.6 | 2.7 | 1.3 | 1.4 |
| rel. $N_{RL}$ | 0 | 0 | 0 | 0.057 | 0 | 0 | 0 | 0.72 | 0.54 | 0.69 | 0.69 | 0.8 | 0.71 | 0.26 | 1.5 | 0.28 | 2.5 | 1.5 | 1 | 1.3 |
| $\Delta O_{RL}$ | 0.89 | 0.89 | 27 | 27 | 30 | 31 | 0.6 | 0.6 | 1.3 | 1.9 | 0.23 | 0.23 | 18 | 14 | 3 | 3.9 | 1.2 | 1.5 | 3.6 | 3.8 |
| rel. $O_{RL}$ | 1.1 | 1.2 | 1.5 | 1.6 | 6.4 | 4.8 | 0.24 | 1.4 | 1.4 | 1.1 | 2.2 | 2.1 | 11 | 8.9 | 2.7 | 2.3 | 2.2 | 2.2 | 1 | 1 |

**Table S6.** Enrichment Factors at 5% subsetting for protein structures of ten PPI targets obtained using post docking derivatized SASA descriptors. The best enrichment score corresponding to each target protein (across columns) is shown in bold and highlighted in green.

| SASA descriptor | BAZ2B | | BCL2 | | BCLXL | | BRD4-1 | | CREBBP | | HIV-IN | | XIAP | | MCL1 | | MDM2 | | Menin | |
|---|---|---|---|---|---|---|---|---|---|---|---|---|---|---|---|---|---|---|---|---|
| | 4XUA | 5E73 | 2O21 | 4LVT | 3INQ | 3WIZ | 5D3L | 5KU3 | 5EIC | 5MMG | 4CFD | 4CHO | 1TFT | 5C3H | 5FC4 | 5MES | 4ODF | 4ZFI | 5DB2 | 6B41 |
| Surflex | 0.88 | 0.89 | 11 | 12 | 10 | 9.7 | 1.4 | 0.43 | 1.6 | **2.7** | **1.6** | **1.6** | **15** | **14** | 2.8 | 2.5 | **2.9** | 1.9 | 1.9 | 1.2 |
| $T_{RU}$ | 0.98 | 1 | 17 | 16 | 16 | 16 | 1.6 | 1.2 | 1.4 | 2 | 0.82 | 0.87 | 8.2 | 7.2 | 4.5 | 3.8 | 2.1 | 1.9 | 1.9 | 1.9 |
| $T_{RB}$ | 0.92 | 0.98 | 11 | 10 | 10 | 12 | 1.7 | 1 | 1.2 | 1.9 | 0.98 | 1.3 | 6 | 5.7 | 2.1 | 2.3 | 1.5 | 1.7 | 1.3 | 1.2 |
| $\Delta T_R$ | 0.94 | 0.99 | **18** | **18** | 17 | 17 | 1.6 | 1.4 | 1.9 | 1.7 | 0.8 | 0.82 | 7.3 | 7.4 | 4.9 | 4.8 | 2.2 | 2 | 2.2 | **2.5** |
| rel. $T_R$ | 1 | 1.1 | 0.011 | 0.023 | 0.062 | 0.062 | 0.78 | 0.24 | 0.52 | 0.8 | 0.75 | 0.66 | 0.19 | 0.11 | 0.63 | 0.7 | 0.21 | 0.74 | 0.66 | 0.88 |
| $H_{RU}$ | 0.94 | 1 | 16 | 16 | 15 | 16 | 1.6 | 1.2 | 1.4 | 2.1 | 1 | 0.87 | 4.4 | 7.2 | 4 | 3.5 | 2 | 1.9 | 1.7 | 1.8 |
| $H_{RB}$ | 0.94 | 0.98 | 7.4 | 6.2 | 9.3 | 11 | 1.3 | 0.83 | 1.2 | 2.1 | 1.2 | 1.1 | 2.8 | 5 | 1.7 | 2.2 | 0.93 | 1.6 | 1.3 | 1.6 |
| $\Delta H_R$ | 0.97 | 1 | 17 | 16 | 16 | 16 | 1.6 | 1.4 | 1.9 | 1.6 | 0.82 | 0.87 | 6 | 9.2 | 4.7 | 3.8 | 2.1 | 2.1 | 2.1 | 2.1 |
| rel. $H_R$ | 0.9 | 0.99 | 0.034 | 0.068 | 0.48 | 0.12 | 0.26 | 0.17 | 0.48 | 0.52 | 0.78 | 0.69 | 0 | 0.16 | 0.63 | 0.84 | 0.18 | 0.81 | 0.74 | 1.3 |
| $Ar_{RU}$ | 0.66 | 0.72 | 6 | 12 | 2.6 | 6.9 | 1.6 | 1.4 | 1.7 | 1.4 | 0.59 | 0.75 | 2.2 | 1.4 | 2.5 | 2.2 | 1.8 | 1.6 | 1.4 | 1.4 |
| $Ar_{RB}$ | 0.93 | 0.97 | 0.63 | 0.7 | 0.083 | 3.7 | 0.47 | 0.74 | 1.5 | 1.3 | 0.71 | 0.91 | 1.1 | 0.19 | 1.5 | 1.4 | 1.1 | 1 | 1.1 | 1.2 |
| $\Delta Ar_R$ | 1 | 1 | 12 | 14 | 12 | 8.2 | 1.5 | 1.5 | 1.1 | 1.2 | 0.78 | 0.71 | 2.7 | 2.9 | 3.2 | 2.9 | 2 | 1.9 | 1.5 | 1.5 |
| rel. $Ar_R$ | 0.88 | 1 | 0.1 | 0.079 | 0.042 | 0.42 | 0.095 | 0.19 | 0.77 | 0.54 | 0.96 | 0.57 | 0.12 | 0.018 | 0.58 | 0.56 | 0.42 | 0.41 | 1.1 | 0.77 |
| $D_{RU}$ | 0.25 | 0.7 | 10 | 10 | 11 | 7 | **2** | 1.1 | 1 | 1.4 | 0.87 | 0.96 | 5.4 | 2.3 | 2.1 | 3 | 1.6 | 1.5 | 1.4 | 1.7 |
| $D_{RB}$ | 1.1 | 1.1 | 6.5 | 0.93 | 0.71 | 1.8 | 1.9 | 1.1 | 0.94 | 1.2 | 0.87 | 0.98 | 3.3 | 1.3 | 1.1 | 1.4 | 1.5 | 1.3 | 1.1 | 0.68 |
| $\Delta D_R$ | 0.91 | 0.98 | 8.2 | 12 | 9.7 | 10 | 0.9 | 1.3 | 0.91 | 2.5 | 0.87 | 0.98 | 4.7 | 2.8 | 2.7 | 2.8 | 1.4 | 2 | 1.5 | **2.5** |
| rel. $D_R$ | 1.1 | 1.1 | 0.045 | 0.045 | 0.021 | 0.021 | 1.4 | 1.1 | 0.71 | 0.45 | 0.71 | 1 | 0.28 | 0.25 | 0.32 | 0.49 | 0.39 | 0.18 | 0.8 | 0.77 |
| $Ac_{RU}$ | 0.98 | 0.85 | 12 | 6.6 | 5.4 | 8.9 | 0.95 | 1.1 | 2 | 1.4 | 1.1 | 1.5 | 5.5 | 5.4 | 3.3 | 2.1 | 2.2 | 2 | 1.1 | 1.8 |
| $Ac_{RB}$ | 0.96 | 0.94 | 10 | 4.5 | 4.1 | 2.2 | 0.85 | 1.1 | 1.7 | 1.2 | 1.3 | 1.1 | 0.58 | 2.6 | 2.6 | 1.4 | 2 | 1.9 | 1.1 | 1.1 |
| $\Delta Ac_R$ | 1.1 | 1 | 13 | 7.1 | 7.5 | 9.6 | 0.83 | 1.1 | 1.9 | 1.7 | 0.94 | 1.1 | 3.7 | 3.9 | 3.7 | 3 | 1.4 | 1.6 | 1.1 | 1.8 |
| rel. $Ac_R$ | 1.1 | 1 | 2.6 | 0.29 | 0.29 | 0.27 | 0.76 | 0.62 | 1 | 0.69 | 0.59 | 0.55 | 0.088 | 0.9 | 0.65 | 0.68 | 1.3 | 1.3 | 1 | 1.1 |
| $P_{RU}$ | 0.0088 | 0.015 | 1.1 | 1.2 | 1.3 | 4 | 1.2 | 0.21 | 0.83 | 1.4 | 1.4 | **1.6** | 3 | 1.8 | 0.31 | 2.2 | 2 | 0.36 | 1.1 | 1.3 |
| $P_{RB}$ | 0.0088 | 0.015 | 0.68 | 0.82 | 1.6 | 4.7 | 1 | 0.21 | 0.97 | 1.3 | 1.1 | **1.6** | 2 | 0.81 | 1.5 | 2 | 1.9 | 0.36 | 0.83 | 1.4 |

| | | | | | | | | | | | | | | | | | | | | |
|---|---|---|---|---|---|---|---|---|---|---|---|---|---|---|---|---|---|---|---|---|
| $\Delta P_R$ | 0.0058 | 0.015 | 7.1 | 7.1 | 4.3 | 6 | 1 | 0.19 | 0.98 | 0.95 | 0.78 | 1.5 | 3.2 | 3 | 0.24 | 3.8 | 1.4 | 0.33 | 1.5 | 1.1 |
| rel. $P_R$ | 0.0088 | 0.015 | 0.18 | 0.09 | 0 | 0.6 | 0.97 | 0.21 | 1.7 | 0.92 | 1.2 | 1.5 | 1.8 | 0 | 1.3 | 0.56 | 0.91 | 0.36 | 1.2 | 1.4 |
| $N_{RU}$ | 0.11 | 0.15 | 13 | 8.1 | 10 | 7.3 | 1.7 | 1.4 | 0.74 | 1.7 | 0.82 | 1.1 | 0.69 | 2.3 | 0 | 0.042 | 0.23 | 0.59 | 1.5 | 1.5 |
| $N_{RB}$ | 0.11 | 0.15 | 4.2 | 5.4 | 6.5 | 6.7 | 1.9 | 1.4 | 0.91 | 1.7 | 1.3 | 1.3 | 1.4 | 2.3 | 0.73 | 1 | 0.23 | 0.59 | 1.3 | 1.2 |
| $\Delta N_R$ | 0.24 | 0.31 | 15 | 9.1 | 6.7 | 10 | 1.2 | 1.5 | 0.57 | 1.5 | 0.87 | 1 | 2.7 | 4.2 | 2.3 | 2.1 | 0.22 | 0.58 | 1.7 | 1.7 |
| rel. $N_R$ | 0 | 0 | 0.09 | 0.11 | 0.17 | 0 | 0.62 | 1.1 | 0.82 | 1.4 | 0.96 | 1.1 | 0.49 | 0.088 | 0.73 | 0.97 | 0.23 | 0.59 | 1.2 | 0.88 |
| $O_{RU}$ | 0 | 0.0029 | 1.3 | 8.7 | 9.3 | 4.9 | 0.12 | 0.14 | 1.7 | 0.8 | 1.3 | 1.1 | 1.2 | 0.49 | 0.72 | 0 | 1.3 | 1 | 1.3 | 1.2 |
| $O_{RB}$ | 0.99 | 0.97 | 0.56 | 2 | 1.4 | 0.89 | 0.14 | 0.24 | 1.3 | 0.71 | 1 | 0.82 | 1 | 3.1 | 1.3 | 0 | 1.2 | 1.4 | 0.91 | 1.2 |
| $\Delta O_R$ | 0.97 | 0.92 | 0.56 | 8.5 | 3.1 | 1.7 | 1.1 | 0.21 | 2.1 | 1.3 | 1.4 | 1.2 | 1.2 | 4.6 | 0 | 0 | 0.61 | 1.7 | 1.2 | 1.4 |
| rel. $O_R$ | 0.98 | 1.1 | 0 | 0.32 | 0.48 | 0 | 0.14 | 0.24 | 0.35 | 0.65 | 1 | 0.85 | 0 | 0 | 1.3 | 0 | 0.93 | 0.32 | 0.77 | 1.3 |
| $T_{LU}$ | 0.96 | 0.97 | **18** | **18** | **18** | **18** | 1.4 | 1.3 | 1.8 | 1.9 | 0.78 | 0.8 | 13 | 13 | **5.4** | **5.2** | 2.1 | 2.1 | 2.4 | 2.3 |
| $T_{LB}$ | 1 | 1.1 | 17 | 17 | 17 | 16 | 1.3 | 1.1 | 2 | 1.4 | 0.82 | 0.82 | 9.3 | 12 | 5.2 | **5.2** | 2 | 2.1 | 1.9 | 2.1 |
| $\Delta T_L$ | 0.93 | 0.89 | 17 | 17 | 16 | 17 | 1.3 | 1 | 1.3 | 1.5 | 0.82 | 1.1 | 12 | 7.6 | 4.3 | 4.3 | 2.6 | 2.4 | **2.7** | 2.4 |
| rel. $T_L$ | 1 | 1.1 | 3 | 3.9 | 7.4 | 5.2 | 1.1 | 1.4 | 1.4 | 0.91 | 0.85 | 0.8 | 5.2 | 7.1 | 3 | 2.6 | 1.3 | 1.7 | 1.2 | 1.4 |
| $H_{LU}$ | 1.1 | 1.1 | 9.3 | 9.1 | 8.5 | 9.2 | 1.1 | 1.1 | 1.6 | 1.6 | 0.69 | 0.64 | 1.8 | 1.9 | 3.3 | 3.5 | 1.8 | 1.9 | 0.68 | 0.74 |
| $H_{LB}$ | 0.98 | 1.1 | 8.9 | 6.2 | 8.2 | 8.6 | 1.2 | 1.6 | 1.8 | 2.3 | 1.1 | 1.1 | 4 | 4.4 | 3.3 | 3.3 | 1.6 | 2.4 | 0.77 | 0.83 |
| $\Delta H_L$ | 1.1 | 1 | 6.9 | 8.8 | 7.1 | 6.5 | 0.85 | 0.57 | 1.4 | 1.3 | 0.75 | 0.55 | 0.21 | 0.16 | 2.5 | 2.2 | 2.4 | 2.2 | 0.97 | 0.71 |
| rel. $H_L$ | 0.98 | 0.96 | 3.2 | 1.8 | 3.8 | 2.4 | 1.6 | **1.8** | 1.1 | 1.5 | 1.5 | 1.4 | 3.8 | 5.8 | 1.1 | 1.3 | 1.3 | 1.9 | 1 | 1.2 |
| $Ar_{LU}$ | 1.2 | 1.1 | 5.3 | 4.7 | 11 | 12 | 0.9 | 0.81 | 2.2 | 2.2 | 1.1 | 1 | 1.7 | 2.1 | 4.7 | 4.5 | 0.27 | 0.24 | 0.57 | 0.6 |
| $Ar_{LB}$ | 1.1 | 1.1 | 6.1 | 5.2 | 11 | 10 | 1.1 | 1.1 | 1.6 | 1.6 | 0.82 | 1.1 | 4.5 | 5.6 | 4.3 | 3.9 | 0.43 | 0.49 | 0.68 | 0.68 |
| $\Delta Ar_L$ | 1.2 | 1.2 | 4.5 | 3.8 | 6.8 | 9 | 0.64 | 0.43 | **2.5** | 2.3 | 0.94 | 0.8 | 0.3 | 0.16 | 3.9 | 3.4 | 0.21 | 0.19 | 0.63 | 0.77 |
| rel. $Ar_L$ | 0.92 | 1 | 2.4 | 1.3 | 3.2 | 2 | 0.9 | 1.3 | 0.34 | 0.57 | 1.1 | 1.1 | 3.8 | 5 | 0.94 | 0.84 | 1.4 | 1.8 | 1.1 | 1.2 |
| $D_{LU}$ | 1.2 | **1.3** | 1.3 | 1.4 | 1.6 | 1.6 | 1 | 1 | 0.48 | 0.46 | 0.78 | 0.78 | 5.4 | 5.5 | 1.1 | 1.1 | 1.5 | 1.5 | 0.77 | 0.8 |
| $D_{LB}$ | 1.1 | 1 | 3 | 3.5 | 2.5 | 2.6 | 1.3 | 1.6 | 0.72 | 0.71 | 0.89 | 0.87 | 3.8 | 3 | 1.6 | 1.8 | 1.7 | 1.5 | 0.97 | 1 |
| $\Delta D_L$ | 1.2 | 1.2 | 1.5 | 1.2 | 1.4 | 1.4 | 0.88 | 0.78 | 0.52 | 0.57 | 0.66 | 0.64 | 4.7 | 4.9 | 1.3 | 1.1 | 1.3 | 1.4 | 0.77 | 0.91 |
| rel. $D_L$ | 0.88 | 0.98 | 1.5 | 1.5 | 2.6 | 2.4 | 1.2 | 1.4 | 1 | 1 | 1.1 | 1.1 | 0.19 | 0.053 | 1.3 | 1.4 | 1 | 0.81 | 1.3 | 1.3 |
| $Ac_{LU}$ | **1.3** | 1.2 | 11 | 11 | 7.9 | 8.4 | 1.3 | 1.4 | 0.38 | 0.43 | 1.1 | 1.1 | 1.2 | 1.4 | 3.3 | 3.3 | 1.4 | 1.4 | 0.37 | 0.43 |
| $Ac_{LB}$ | 1 | 1.1 | 12 | 10 | 7.7 | 9 | 1.2 | 1.4 | 1.1 | 0.62 | 1.2 | 1 | 2.4 | 2.5 | 3.3 | 2.9 | 1.6 | 2 | 0.71 | 0.83 |
| $\Delta Ac_L$ | **1.3** | 1.2 | 4.8 | 4.6 | 4.4 | 3.8 | 1.3 | 0.97 | 0.42 | 0.55 | 1.1 | 1.2 | 0.44 | 0.34 | 2.3 | 1.9 | 1.2 | 0.94 | 0.43 | 0.26 |
| rel. $Ac_L$ | 1.1 | 1 | 3.6 | 2.2 | 2.7 | 3.2 | 0.55 | 0.78 | 1.1 | 0.72 | 1.1 | 1.3 | 0.79 | 0.65 | 1.5 | 1.2 | 1.2 | 1.1 | 1 | 0.74 |

| | | | | | | | | | | | | | | | | | | | | |
|---|---|---|---|---|---|---|---|---|---|---|---|---|---|---|---|---|---|---|---|---|
| $P_{LU}$ | **1.3** | **1.3** | 2.5 | 2.5 | 3.3 | 1.9 | 0.36 | 0.36 | 0.43 | 0.43 | 0.39 | 0.39 | 2.6 | 2.6 | 1.5 | 1.5 | 0.91 | 0.88 | 0.57 | 0.66 |
| $P_{LB}$ | 0.59 | 0.77 | 3.6 | 3.3 | 2.8 | 2.7 | 0.17 | 0.21 | 0.66 | 0.65 | 0.37 | 0.48 | 0.88 | 0.56 | 2 | 2.1 | 0.87 | 0.65 | 0.43 | 0.74 |
| $\Delta P_L$ | **1.3** | **1.3** | 2.8 | 4.2 | 3.3 | 2.8 | 0.36 | 0.36 | 0.42 | 0.42 | 0.37 | 0.32 | 2.4 | 2.5 | 1.6 | 1.4 | 0.9 | 0.89 | 0.74 | 0.51 |
| rel. $P_L$ | 0.59 | 0.77 | 4.7 | 4.4 | 2.9 | 4 | 0.17 | 0.21 | 0.66 | 0.65 | 0.37 | 0.48 | 0.88 | 0.58 | 2 | 2.4 | 1.5 | 0.63 | 0.4 | 0.74 |
| $N_{LU}$ | 0.77 | 0.77 | 1.7 | 1.7 | 7.6 | 7.6 | 0.24 | 0.24 | 0.35 | 0.34 | 0.82 | 1.3 | 0.95 | 0.95 | 4.5 | 4.4 | 2.8 | **2.8** | 0.63 | 0.68 |
| $N_{LB}$ | 0.72 | 0.71 | 1.6 | 1.7 | 7.5 | 7.4 | 0.24 | 0.24 | 0.26 | 0.25 | 0.78 | 1.1 | 0.94 | 0.95 | 4.5 | 4.4 | 2.8 | **2.8** | 0.66 | 0.83 |
| $\Delta N_L$ | 0.76 | 0.76 | 1.7 | 1.6 | 7.5 | 7.5 | 0.24 | 0.24 | 0.2 | 0.28 | 1.2 | 1.1 | 0.86 | 0.85 | 4.4 | 4.5 | 2.8 | **2.8** | 0.66 | 0.54 |
| rel. $N_L$ | 0.72 | 0.71 | 1.6 | 1.7 | 7.5 | 7.4 | 0.24 | 0.24 | 0.26 | 0.25 | 0.8 | 1.2 | 0.94 | 0.95 | 4.6 | 4.5 | 2.8 | **2.8** | 0.66 | 0.83 |
| $O_{LU}$ | 0.78 | 0.82 | 17 | 17 | 14 | 14 | 1 | 0.9 | 1.2 | 1.3 | 0.69 | 0.69 | 12 | 12 | 2.7 | 2.7 | 1.6 | 1.6 | 2.3 | 2.3 |
| $O_{LB}$ | 1 | 0.94 | 12 | 14 | 13 | 12 | 1.2 | 1.1 | 1.6 | 1.1 | 0.69 | 0.71 | 9.7 | 12 | 2.9 | 2.8 | 1.9 | 1.8 | 2.2 | 2.1 |
| $\Delta O_L$ | 0.76 | 0.85 | 15 | 15 | 11 | 12 | 0.88 | 0.85 | 0.88 | 1.1 | 0.57 | 0.5 | 9.4 | 6.9 | 1.9 | 2.1 | 1.3 | 1.4 | 2.3 | 2.3 |
| rel. $O_L$ | 1.1 | 1.1 | 1.2 | 1.8 | 4 | 2.5 | 1 | 1.2 | 1.4 | 0.88 | 0.82 | 0.85 | 3.6 | 4.2 | 2.2 | 2.1 | 1.6 | 1.6 | 1.1 | 1.2 |
| $\Delta T_{RL}$ | 0.89 | 0.93 | **18** | **18** | 17 | 17 | 1.4 | 1.2 | 1.7 | 1.8 | 0.78 | 0.94 | 11 | 7.9 | 4.8 | 4.6 | 2.4 | 2.2 | 2.5 | **2.5** |
| rel. $T_{RL}$ | 1.1 | 1.1 | 0.36 | 0.55 | 2.3 | 1.7 | 0.83 | 1.3 | 0.92 | 0.58 | 0.94 | 0.94 | 3 | 5 | 1.3 | 1.5 | 0.83 | 1.5 | 0.83 | 1 |
| $\Delta H_{RL}$ | 1.1 | 1 | 16 | 15 | 13 | 15 | 1.4 | 1.1 | 2.3 | 1.7 | 0.91 | 0.64 | 1.9 | 2.5 | 4.5 | 3.9 | 2.8 | 2.4 | 1.8 | 1.6 |
| rel. $H_{RL}$ | 0.95 | 1 | 0.11 | 0.24 | 1 | 0.27 | 0.76 | 1.4 | 0.45 | 0.65 | 1.3 | 1.5 | 0.56 | 2.5 | 0.65 | 1.2 | 0.35 | 1.5 | 0.85 | 0.94 |
| $\Delta Ar_{RL}$ | 1.2 | 1.1 | 8.6 | 9.7 | 13 | 12 | 1.2 | 0.85 | **2.5** | 2.2 | 0.87 | 0.8 | 1.5 | 0.74 | 4.3 | 3.8 | 0.35 | 0.54 | 0.88 | 0.94 |
| rel. $Ar_{RL}$ | 1 | 1 | 0.15 | 0.44 | 0.62 | 1.1 | 0.66 | 0.76 | 0.34 | 0.31 | 1 | 0.94 | 1.5 | 1.4 | 0.59 | 0.25 | 0.8 | 1.1 | 1 | 1.2 |
| $\Delta D_{RL}$ | 1.2 | **1.3** | 6.9 | 9.2 | 7.7 | 3.7 | 0.88 | 0.85 | 0.55 | 0.31 | 0.75 | 0.78 | 7.4 | 8.3 | 1.8 | 1.9 | 1.4 | 1.8 | 1 | 1.2 |
| rel. $D_{RL}$ | 1.1 | 1 | 0.068 | 0 | 0.021 | 0.25 | 1.4 | 1.3 | 1.1 | 0.69 | 0.87 | 0.87 | 0.071 | 0.11 | 0.51 | 0.46 | 0.65 | 0.39 | 0.8 | 0.91 |
| $\Delta Ac_{RL}$ | 1.2 | 1.2 | 7.7 | 7.2 | 6.5 | 4.8 | 1.2 | 1 | 0.57 | 0.66 | 1 | 1.3 | 0.86 | 0.99 | 2.4 | 2.4 | 1.3 | 1.1 | 0.48 | 0.43 |
| rel. $Ac_{RL}$ | 0.97 | 1 | 4.8 | 1.8 | 1.6 | 4.8 | 0.83 | 0.93 | 0.94 | 0.48 | 1 | 1.2 | 1 | 1.1 | 1.4 | 1.2 | 1.5 | 1.3 | 1.1 | 0.97 |
| $\Delta P_{RL}$ | **1.3** | **1.3** | 7.1 | 7.1 | 4.5 | 6.1 | 0.97 | 0.4 | 0.97 | 0.95 | 0.75 | 1.3 | 3.1 | 3.1 | 1.4 | 3.9 | 1.4 | 1 | 1.5 | 0.54 |
| rel. $P_{RL}$ | 0.6 | 0.78 | 0.19 | 0.12 | 0.042 | 0.64 | 0.97 | 0.28 | 1.7 | 0.94 | 1.2 | 1.3 | 1.9 | 0 | 1.2 | 0.52 | 0.87 | 0.72 | 1.1 | 1.3 |
| $\Delta N_{RL}$ | 0.94 | 0.91 | 13 | 6 | 9.6 | 10 | 1 | 1.1 | 0.2 | 0.28 | 1 | 1.1 | 1.9 | 2.8 | 4.4 | 4.6 | 2.8 | 2.7 | 0.85 | 0.77 |
| rel. $N_{RL}$ | 0 | 0 | 0.079 | 0.079 | 0 | 0 | 0.64 | 1.1 | 0.82 | 1.4 | 1 | 1.3 | 0.53 | 0.18 | 0.96 | 0.94 | 2.7 | 2 | 1.2 | 0.97 |
| $\Delta O_{RL}$ | 0.77 | 0.83 | 15 | 15 | 11 | 12 | 0.85 | 0.85 | 0.94 | 1.1 | 0.57 | 0.5 | 9.4 | 7.3 | 1.9 | 2.1 | 1.3 | 1.4 | 2.4 | 2.3 |
| rel. $O_{RL}$ | 1.1 | 1.1 | 1.2 | 1.8 | 3.9 | 2.2 | 1 | 1.2 | 1.3 | 0.8 | 0.82 | 0.82 | 3.6 | 4.3 | 2.2 | 2.1 | 1.5 | 1.5 | 1.1 | 1.2 |

**Table S7.** 10-fold Cross-Validation TN, FP, FN, TP, Total, Sensitivity, Specificity, Precision, Concordance (Accuracy), J, MCC, $F_1$, k, ROC score, EF 1%, and EF 5% values for the different machine learning models obtained on 10 PPI targets with a total of 20 protein structures.

| Target | PDB ID | ML techniques | TN | FP | FN | TP | Total | Sensitivity | Specificity | Precision | Concordance | J | MCC | $F_1$ | k | ROC score | EF 1% | EF 5% |
|---|---|---|---|---|---|---|---|---|---|---|---|---|---|---|---|---|---|---|
| BAZ2B | 4XUA | Tree | 34598 | 0 | 4794 | 0 | 39392 | 0.000 | 1.000 | N/A | 0.878 | 0.000 | N/A | N/A | 0.000 | 0.543 | 2.127 | 1.633 |
| | | Bagged forest | 34598 | 0 | 4794 | 0 | 39392 | 0.000 | 1.000 | N/A | 0.878 | 0.000 | N/A | N/A | 0.000 | 0.500 | 1.000 | 1.000 |
| | | Random forest | 34598 | 0 | 4794 | 0 | 39392 | 0.000 | 1.000 | N/A | 0.878 | 0.000 | N/A | N/A | 0.000 | 0.601 | 3.931 | 2.345 |
| | | Bayesian | 34543 | 55 | 4722 | 72 | 39392 | 0.015 | 0.998 | 0.567 | 0.879 | 0.013 | 0.077 | 0.029 | 0.023 | 0.602 | 3.136 | 2.032 |
| | | SVM | 34492 | 106 | 4181 | 613 | 39392 | 0.128 | 0.997 | 0.853 | 0.891 | 0.125 | 0.305 | 0.222 | 0.197 | 0.758 | 7.464 | 5.125 |
| | | Logistic regression | 34598 | 0 | 4794 | 0 | 39392 | 0.000 | 1.000 | N/A | 0.878 | 0.000 | N/A | N/A | 0.000 | 0.550 | 1.296 | 1.081 |
| | | Neural net | 34598 | 0 | 4794 | 0 | 39392 | 0.000 | 1.000 | N/A | 0.878 | 0.000 | N/A | N/A | 0.000 | 0.543 | 1.066 | 1.181 |
| | | Neural net (bagging) | 34598 | 0 | 4794 | 0 | 39392 | 0.000 | 1.000 | N/A | 0.878 | 0.000 | N/A | N/A | 0.000 | 0.651 | 3.952 | 2.667 |
| | 5E73 | Tree | 34598 | 0 | 4794 | 0 | 39392 | 0.000 | 1.000 | N/A | 0.878 | 0.000 | N/A | N/A | 0.000 | 0.534 | 1.922 | 1.490 |
| | | Bagged forest | 34598 | 0 | 4794 | 0 | 39392 | 0.000 | 1.000 | N/A | 0.878 | 0.000 | N/A | N/A | 0.000 | 0.500 | 1.000 | 1.000 |
| | | Random forest | 34598 | 0 | 4794 | 0 | 39392 | 0.000 | 1.000 | N/A | 0.878 | 0.000 | N/A | N/A | 0.000 | 0.631 | 3.617 | 2.391 |
| | | Bayesian | 34499 | 99 | 4699 | 95 | 39392 | 0.020 | 0.997 | 0.490 | 0.878 | 0.017 | 0.079 | 0.038 | 0.029 | 0.602 | 3.178 | 2.070 |
| | | SVM | 34492 | 106 | 4065 | 729 | 39392 | 0.152 | 0.997 | 0.873 | 0.894 | 0.149 | 0.338 | 0.259 | 0.231 | 0.772 | 7.569 | 5.738 |
| | | Logistic regression | 34598 | 0 | 4793 | 1 | 39392 | 0.000 | 1.000 | 1.000 | 0.878 | 0.000 | 0.014 | 0.000 | 0.000 | 0.535 | 1.443 | 1.089 |
| | | Neural net | 34598 | 0 | 4794 | 0 | 39392 | 0.000 | 1.000 | N/A | 0.878 | 0.000 | N/A | N/A | 0.000 | 0.542 | 1.422 | 1.294 |
| | | Neural net (bagging) | 34598 | 0 | 4794 | 0 | 39392 | 0.000 | 1.000 | N/A | 0.878 | 0.000 | N/A | N/A | 0.000 | 0.671 | 4.286 | 2.942 |
| Bcl-2 | 2O21 | Tree | 34305 | 38 | 166 | 1074 | 35583 | 0.866 | 0.999 | 0.966 | 0.994 | 0.865 | 0.912 | 0.913 | 0.910 | 0.965 | 28.557 | 18.313 |
| | | Bagged forest | 34262 | 81 | 147 | 1093 | 35583 | 0.881 | 0.998 | 0.931 | 0.994 | 0.879 | 0.903 | 0.906 | 0.902 | 0.943 | 27.496 | 17.798 |
| | | Random forest | 34305 | 38 | 152 | 1088 | 35583 | 0.877 | 0.999 | 0.966 | 0.995 | 0.876 | 0.918 | 0.920 | 0.917 | 0.999 | 28.696 | 19.663 |
| | | Bayesian | 33822 | 521 | 124 | 1116 | 35583 | 0.900 | 0.985 | 0.682 | 0.982 | 0.885 | 0.775 | 0.776 | 0.767 | 0.984 | 28.546 | 18.066 |
| | | SVM | 34322 | 21 | 99 | 1141 | 35583 | 0.920 | 0.999 | 0.982 | 0.997 | 0.920 | 0.949 | 0.950 | 0.948 | 0.990 | 28.696 | 19.292 |
| | | Logistic regression | 34318 | 25 | 418 | 822 | 35583 | 0.663 | 0.999 | 0.970 | 0.988 | 0.662 | 0.797 | 0.788 | 0.782 | 0.973 | 27.483 | 18.098 |
| | | Neural net | 34307 | 36 | 170 | 1070 | 35583 | 0.863 | 0.999 | 0.967 | 0.994 | 0.862 | 0.911 | 0.912 | 0.909 | 0.992 | 28.696 | 18.937 |

| | | | | | | | | | | | | | | | | | |
|---|---|---|---|---|---|---|---|---|---|---|---|---|---|---|---|---|---|
| | | Neural net (bagging) | 34341 | 2 | 27 | 1213 | 35583 | 0.978 | 1.000 | 0.998 | 0.999 | 0.978 | 0.988 | 0.988 | 0.988 | 1.000 | 28.696 | 20.002 |
| | 4LVT | Tree | 34311 | 32 | 173 | 1067 | 35583 | 0.860 | 0.999 | 0.971 | 0.994 | 0.860 | 0.911 | 0.912 | 0.909 | 0.948 | 28.581 | 17.984 |
| | | Bagged forest | 34259 | 84 | 155 | 1085 | 35583 | 0.875 | 0.998 | 0.928 | 0.993 | 0.873 | 0.898 | 0.901 | 0.897 | 0.940 | 27.278 | 17.687 |
| | | Random forest | 34310 | 33 | 151 | 1089 | 35583 | 0.878 | 0.999 | 0.971 | 0.995 | 0.877 | 0.921 | 0.922 | 0.919 | 0.998 | 28.696 | 19.469 |
| | | Bayesian | 33886 | 457 | 125 | 1115 | 35583 | 0.899 | 0.987 | 0.709 | 0.984 | 0.886 | 0.791 | 0.793 | 0.785 | 0.984 | 28.516 | 18.227 |
| | | SVM | 34324 | 19 | 117 | 1123 | 35583 | 0.906 | 0.999 | 0.983 | 0.996 | 0.905 | 0.942 | 0.943 | 0.941 | 0.988 | 28.696 | 19.292 |
| | | Logistic regression | 34316 | 27 | 451 | 789 | 35583 | 0.636 | 0.999 | 0.967 | 0.987 | 0.636 | 0.779 | 0.768 | 0.761 | 0.979 | 27.079 | 18.195 |
| | | Neural net | 34299 | 44 | 164 | 1076 | 35583 | 0.868 | 0.999 | 0.961 | 0.994 | 0.866 | 0.910 | 0.912 | 0.909 | 0.989 | 28.615 | 18.889 |
| | | Neural net (bagging) | 34341 | 2 | 16 | 1224 | 35583 | 0.987 | 1.000 | 0.998 | 0.999 | 0.987 | 0.992 | 0.993 | 0.992 | 1.000 | 28.696 | 20.002 |
| Bcl-xL | 3INQ | Tree | 34382 | 38 | 131 | 543 | 35094 | 0.806 | 0.999 | 0.935 | 0.995 | 0.805 | 0.865 | 0.865 | 0.863 | 0.971 | 51.306 | 18.616 |
| | | Bagged forest | 34346 | 74 | 141 | 533 | 35094 | 0.791 | 0.998 | 0.878 | 0.994 | 0.789 | 0.830 | 0.832 | 0.829 | 0.907 | 46.798 | 16.444 |
| | | Random forest | 34401 | 19 | 129 | 545 | 35094 | 0.809 | 0.999 | 0.966 | 0.996 | 0.808 | 0.882 | 0.880 | 0.878 | 0.997 | 52.068 | 19.563 |
| | | Bayesian | 33764 | 656 | 72 | 602 | 35094 | 0.893 | 0.981 | 0.479 | 0.979 | 0.874 | 0.645 | 0.623 | 0.614 | 0.983 | 49.688 | 18.405 |
| | | SVM | 34404 | 16 | 72 | 602 | 35094 | 0.893 | 1.000 | 0.974 | 0.997 | 0.893 | 0.932 | 0.932 | 0.931 | 0.991 | 51.771 | 19.266 |
| | | Logistic regression | 34402 | 18 | 511 | 163 | 35094 | 0.242 | 0.999 | 0.901 | 0.985 | 0.241 | 0.462 | 0.381 | 0.376 | 0.980 | 46.861 | 18.494 |
| | | Neural net | 34379 | 41 | 123 | 551 | 35094 | 0.818 | 0.999 | 0.931 | 0.995 | 0.816 | 0.870 | 0.870 | 0.868 | 0.987 | 51.919 | 19.028 |
| | | Neural net (bagging) | 34418 | 2 | 7 | 667 | 35094 | 0.990 | 1.000 | 0.997 | 1.000 | 0.990 | 0.993 | 0.993 | 0.993 | 1.000 | 52.068 | 20.008 |
| | 3WIZ | Tree | 34399 | 21 | 159 | 515 | 35094 | 0.764 | 0.999 | 0.961 | 0.995 | 0.763 | 0.854 | 0.851 | 0.849 | 0.973 | 50.892 | 18.713 |
| | | Bagged forest | 34352 | 68 | 148 | 526 | 35094 | 0.780 | 0.998 | 0.886 | 0.994 | 0.778 | 0.828 | 0.830 | 0.827 | 0.906 | 46.432 | 16.415 |
| | | Random forest | 34396 | 24 | 128 | 546 | 35094 | 0.810 | 0.999 | 0.958 | 0.996 | 0.809 | 0.879 | 0.878 | 0.876 | 0.998 | 52.068 | 19.622 |
| | | Bayesian | 31015 | 3405 | 43 | 631 | 35094 | 0.936 | 0.901 | 0.156 | 0.902 | 0.837 | 0.360 | 0.268 | 0.243 | 0.977 | 50.581 | 17.663 |
| | | SVM | 34420 | 0 | 14 | 660 | 35094 | 0.979 | 1.000 | 1.000 | 1.000 | 0.979 | 0.989 | 0.990 | 0.989 | 1.000 | 52.068 | 19.949 |
| | | Logistic regression | 34405 | 15 | 577 | 97 | 35094 | 0.144 | 1.000 | 0.866 | 0.983 | 0.143 | 0.349 | 0.247 | 0.243 | 0.975 | 47.456 | 18.405 |
| | | Neural net | 34397 | 23 | 127 | 547 | 35094 | 0.812 | 0.999 | 0.960 | 0.996 | 0.811 | 0.880 | 0.879 | 0.877 | 0.988 | 44.641 | 15.979 |
| | | Neural net (bagging) | 34420 | 0 | 13 | 661 | 35094 | 0.981 | 1.000 | 1.000 | 1.000 | 0.981 | 0.990 | 0.990 | 0.990 | 1.000 | 52.068 | 20.008 |
| BRD4-1 | 5D3L | Tree | 624 | 63 | 73 | 519 | 1279 | 0.877 | 0.908 | 0.892 | 0.894 | 0.785 | 0.786 | 0.884 | 0.786 | 0.962 | 2.155 | 2.150 |
| | | Bagged forest | 638 | 49 | 66 | 526 | 1279 | 0.889 | 0.929 | 0.915 | 0.910 | 0.817 | 0.819 | 0.901 | 0.819 | 0.972 | 2.160 | 2.160 |
| | | Random forest | 614 | 73 | 59 | 533 | 1279 | 0.900 | 0.894 | 0.880 | 0.897 | 0.794 | 0.793 | 0.890 | 0.793 | 0.968 | 2.160 | 2.160 |
| | | Bayesian | 585 | 102 | 69 | 523 | 1279 | 0.883 | 0.852 | 0.837 | 0.866 | 0.735 | 0.733 | 0.859 | 0.732 | 0.936 | 2.160 | 2.160 |

| | | | | | | | | | | | | | | | | | | |
|---|---|---|---|---|---|---|---|---|---|---|---|---|---|---|---|---|---|---|
| | | SVM | 619 | 68 | 74 | 518 | 1279 | 0.875 | 0.901 | 0.884 | 0.889 | 0.776 | 0.777 | 0.879 | 0.777 | 0.952 | 1.980 | 2.126 |
| | | Logistic regression | 629 | 58 | 139 | 453 | 1279 | 0.765 | 0.916 | 0.886 | 0.846 | 0.681 | 0.693 | 0.821 | 0.687 | 0.917 | 2.160 | 2.092 |
| | | Neural net | 600 | 87 | 79 | 513 | 1279 | 0.867 | 0.873 | 0.855 | 0.870 | 0.740 | 0.739 | 0.861 | 0.739 | 0.947 | 2.160 | 2.160 |
| | | Neural net (bagging) | 687 | 0 | 0 | 592 | 1279 | 1.000 | 1.000 | 1.000 | 1.000 | 1.000 | 1.000 | 1.000 | 1.000 | 1.000 | 2.160 | 2.160 |
| | 5KU3 | Tree | 635 | 52 | 42 | 550 | 1279 | 0.929 | 0.924 | 0.914 | 0.927 | 0.853 | 0.852 | 0.921 | 0.852 | 0.972 | 2.160 | 2.160 |
| | | Bagged forest | 614 | 73 | 50 | 542 | 1279 | 0.916 | 0.894 | 0.881 | 0.904 | 0.809 | 0.808 | 0.898 | 0.807 | 0.966 | 2.160 | 2.160 |
| | | Random forest | 581 | 106 | 55 | 537 | 1279 | 0.907 | 0.846 | 0.835 | 0.874 | 0.753 | 0.751 | 0.870 | 0.748 | 0.957 | 2.160 | 2.160 |
| | | Bayesian | 540 | 147 | 65 | 527 | 1279 | 0.890 | 0.786 | 0.782 | 0.834 | 0.676 | 0.675 | 0.833 | 0.670 | 0.924 | 2.160 | 2.160 |
| | | SVM | 591 | 96 | 88 | 504 | 1279 | 0.851 | 0.860 | 0.840 | 0.856 | 0.712 | 0.711 | 0.846 | 0.711 | 0.927 | 2.160 | 2.126 |
| | | Logistic regression | 611 | 76 | 139 | 453 | 1279 | 0.765 | 0.889 | 0.856 | 0.832 | 0.655 | 0.663 | 0.808 | 0.659 | 0.900 | 2.160 | 2.126 |
| | | Neural net | 581 | 106 | 88 | 504 | 1279 | 0.851 | 0.846 | 0.826 | 0.848 | 0.697 | 0.696 | 0.839 | 0.696 | 0.928 | 2.160 | 2.160 |
| | | Neural net (bagging) | 687 | 0 | 1 | 591 | 1279 | 0.998 | 1.000 | 1.000 | 0.999 | 0.998 | 0.998 | 0.999 | 0.998 | 1.000 | 2.160 | 2.160 |
| CREBBP | 5EIC | Tree | 32425 | 0 | 910 | 0 | 33335 | 0.000 | 1.000 | N/A | 0.973 | 0.000 | N/A | N/A | 0.000 | 0.635 | 5.140 | 3.225 |
| | | Bagged forest | 32425 | 0 | 910 | 0 | 33335 | 0.000 | 1.000 | N/A | 0.973 | 0.000 | N/A | N/A | 0.000 | 0.500 | 1.000 | 1.000 |
| | | Random forest | 32425 | 0 | 910 | 0 | 33335 | 0.000 | 1.000 | N/A | 0.973 | 0.000 | N/A | N/A | 0.000 | 0.887 | 24.531 | 10.356 |
| | | Bayesian | 31019 | 1406 | 677 | 233 | 33335 | 0.256 | 0.957 | 0.142 | 0.938 | 0.213 | 0.160 | 0.183 | 0.153 | 0.794 | 9.020 | 5.211 |
| | | SVM | 32392 | 33 | 535 | 375 | 33335 | 0.412 | 0.999 | 0.919 | 0.983 | 0.411 | 0.609 | 0.569 | 0.562 | 0.938 | 34.872 | 13.545 |
| | | Logistic regression | 32425 | 0 | 910 | 0 | 33335 | 0.000 | 1.000 | N/A | 0.973 | 0.000 | N/A | N/A | 0.000 | 0.766 | 6.820 | 4.683 |
| | | Neural net | 32425 | 0 | 910 | 0 | 33335 | 0.000 | 1.000 | N/A | 0.973 | 0.000 | N/A | N/A | 0.000 | 0.766 | 7.810 | 4.683 |
| | | Neural net (bagging) | 32425 | 0 | 901 | 9 | 33335 | 0.010 | 1.000 | 1.000 | 0.973 | 0.010 | 0.098 | 0.020 | 0.019 | 0.938 | 26.951 | 12.489 |
| | 5MMG | Tree | 32425 | 0 | 910 | 0 | 33335 | 0.000 | 1.000 | N/A | 0.973 | 0.000 | N/A | N/A | 0.000 | 0.714 | 6.344 | 3.832 |
| | | Bagged forest | 32425 | 0 | 910 | 0 | 33335 | 0.000 | 1.000 | N/A | 0.973 | 0.000 | N/A | N/A | 0.000 | 0.500 | 1.000 | 1.000 |
| | | Random forest | 32425 | 0 | 910 | 0 | 33335 | 0.000 | 1.000 | N/A | 0.973 | 0.000 | N/A | N/A | 0.000 | 0.883 | 20.131 | 10.224 |
| | | Bayesian | 29632 | 2793 | 636 | 274 | 33335 | 0.301 | 0.914 | 0.089 | 0.897 | 0.215 | 0.121 | 0.138 | 0.100 | 0.746 | 4.840 | 3.980 |
| | | SVM | 32398 | 27 | 467 | 443 | 33335 | 0.487 | 0.999 | 0.943 | 0.985 | 0.486 | 0.672 | 0.642 | 0.635 | 0.946 | 36.412 | 14.138 |
| | | Logistic regression | 32425 | 0 | 910 | 0 | 33335 | 0.000 | 1.000 | N/A | 0.973 | 0.000 | N/A | N/A | 0.000 | 0.734 | 5.500 | 4.486 |
| | | Neural net | 32425 | 0 | 910 | 0 | 33335 | 0.000 | 1.000 | N/A | 0.973 | 0.000 | N/A | N/A | 0.000 | 0.760 | 6.710 | 4.617 |
| | | Neural net (bagging) | 32425 | 0 | 904 | 6 | 33335 | 0.007 | 1.000 | 1.000 | 0.973 | 0.007 | 0.080 | 0.013 | 0.013 | 0.934 | 23.981 | 11.829 |
| HIV IN | 4CFD | Tree | 829 | 26 | 93 | 517 | 1465 | 0.848 | 0.970 | 0.952 | 0.919 | 0.817 | 0.834 | 0.897 | 0.830 | 0.970 | 2.402 | 2.402 |

| | | | | | | | | | | | | | | | | | | |
|---|---|---|---|---|---|---|---|---|---|---|---|---|---|---|---|---|---|---|
| | | Bagged forest | 801 | 54 | 134 | 476 | 1465 | 0.780 | 0.937 | 0.898 | 0.872 | 0.717 | 0.736 | 0.835 | 0.731 | 0.920 | 2.402 | 2.374 |
| | | Random forest | 771 | 84 | 114 | 496 | 1465 | 0.813 | 0.902 | 0.855 | 0.865 | 0.715 | 0.721 | 0.834 | 0.720 | 0.936 | 2.402 | 2.402 |
| | | Bayesian | 625 | 230 | 122 | 488 | 1465 | 0.800 | 0.731 | 0.680 | 0.760 | 0.531 | 0.524 | 0.735 | 0.518 | 0.827 | 2.402 | 2.171 |
| | | SVM | 720 | 135 | 119 | 491 | 1465 | 0.805 | 0.842 | 0.784 | 0.827 | 0.647 | 0.645 | 0.794 | 0.645 | 0.895 | 2.230 | 2.237 |
| | | Logistic regression | 807 | 48 | 431 | 179 | 1465 | 0.293 | 0.944 | 0.789 | 0.673 | 0.237 | 0.323 | 0.428 | 0.261 | 0.781 | 2.230 | 1.941 |
| | | Neural net | 719 | 136 | 209 | 401 | 1465 | 0.657 | 0.841 | 0.747 | 0.765 | 0.498 | 0.510 | 0.699 | 0.507 | 0.831 | 2.402 | 2.303 |
| | | Neural net (bagging) | 855 | 0 | 0 | 610 | 1465 | 1.000 | 1.000 | 1.000 | 1.000 | 1.000 | 1.000 | 1.000 | 1.000 | 1.000 | 2.402 | 2.402 |
| | 4CHO | Tree | 782 | 73 | 172 | 438 | 1465 | 0.718 | 0.915 | 0.857 | 0.833 | 0.633 | 0.654 | 0.781 | 0.648 | 0.931 | 2.402 | 2.402 |
| | | Bagged forest | 794 | 61 | 146 | 464 | 1465 | 0.761 | 0.929 | 0.884 | 0.859 | 0.689 | 0.709 | 0.818 | 0.703 | 0.928 | 2.402 | 2.402 |
| | | Random forest | 808 | 47 | 165 | 445 | 1465 | 0.730 | 0.945 | 0.904 | 0.855 | 0.675 | 0.704 | 0.808 | 0.694 | 0.929 | 2.402 | 2.402 |
| | | Bayesian | 608 | 247 | 135 | 475 | 1465 | 0.779 | 0.711 | 0.658 | 0.739 | 0.490 | 0.483 | 0.713 | 0.477 | 0.832 | 2.402 | 2.369 |
| | | SVM | 734 | 121 | 141 | 469 | 1465 | 0.769 | 0.858 | 0.795 | 0.821 | 0.627 | 0.631 | 0.782 | 0.630 | 0.896 | 2.402 | 2.336 |
| | | Logistic regression | 805 | 50 | 411 | 199 | 1465 | 0.326 | 0.942 | 0.799 | 0.685 | 0.268 | 0.351 | 0.463 | 0.293 | 0.770 | 2.402 | 2.336 |
| | | Neural net | 744 | 111 | 232 | 378 | 1465 | 0.620 | 0.870 | 0.773 | 0.766 | 0.490 | 0.512 | 0.688 | 0.504 | 0.833 | 2.402 | 2.303 |
| | | Neural net (bagging) | 855 | 0 | 0 | 610 | 1465 | 1.000 | 1.000 | 1.000 | 1.000 | 1.000 | 1.000 | 1.000 | 1.000 | 1.000 | 2.402 | 2.402 |
| XIAP | 1TFT | Tree | 34425 | 103 | 263 | 530 | 35321 | 0.668 | 0.997 | 0.837 | 0.990 | 0.665 | 0.743 | 0.743 | 0.738 | 0.972 | 38.484 | 17.750 |
| | | Bagged forest | 34385 | 143 | 237 | 556 | 35321 | 0.701 | 0.996 | 0.795 | 0.989 | 0.697 | 0.741 | 0.745 | 0.740 | 0.974 | 37.172 | 17.453 |
| | | Random forest | 34509 | 19 | 206 | 587 | 35321 | 0.740 | 0.999 | 0.969 | 0.994 | 0.740 | 0.844 | 0.839 | 0.836 | 0.998 | 44.541 | 19.849 |
| | | Bayesian | 29963 | 4565 | 21 | 772 | 35321 | 0.974 | 0.868 | 0.145 | 0.870 | 0.841 | 0.348 | 0.252 | 0.221 | 0.981 | 36.213 | 17.478 |
| | | SVM | 34479 | 49 | 99 | 694 | 35321 | 0.875 | 0.999 | 0.934 | 0.996 | 0.874 | 0.902 | 0.904 | 0.902 | 0.997 | 43.532 | 19.471 |
| | | Logistic regression | 34514 | 14 | 683 | 110 | 35321 | 0.139 | 1.000 | 0.887 | 0.980 | 0.138 | 0.346 | 0.240 | 0.235 | 0.984 | 36.592 | 18.260 |
| | | Neural net | 34462 | 66 | 159 | 634 | 35321 | 0.799 | 0.998 | 0.906 | 0.994 | 0.798 | 0.848 | 0.849 | 0.846 | 0.994 | 43.658 | 19.294 |
| | | Neural net (bagging) | 34524 | 4 | 8 | 785 | 35321 | 0.990 | 1.000 | 0.995 | 1.000 | 0.990 | 0.992 | 0.992 | 0.992 | 1.000 | 44.541 | 20.001 |
| | 5C3H | Tree | 34409 | 119 | 214 | 579 | 35321 | 0.730 | 0.997 | 0.830 | 0.991 | 0.727 | 0.774 | 0.777 | 0.772 | 0.985 | 42.323 | 18.547 |
| | | Bagged forest | 34357 | 171 | 208 | 585 | 35321 | 0.738 | 0.995 | 0.774 | 0.989 | 0.733 | 0.750 | 0.755 | 0.750 | 0.984 | 38.358 | 18.386 |
| | | Random forest | 34509 | 19 | 221 | 572 | 35321 | 0.721 | 0.999 | 0.968 | 0.993 | 0.721 | 0.832 | 0.827 | 0.823 | 0.998 | 44.541 | 19.849 |
| | | Bayesian | 32991 | 1537 | 85 | 708 | 35321 | 0.893 | 0.955 | 0.315 | 0.954 | 0.848 | 0.515 | 0.466 | 0.448 | 0.981 | 36.339 | 17.024 |
| | | SVM | 34476 | 52 | 104 | 689 | 35321 | 0.869 | 0.998 | 0.930 | 0.996 | 0.867 | 0.897 | 0.898 | 0.896 | 0.998 | 43.910 | 19.698 |
| | | Logistic regression | 34513 | 15 | 670 | 123 | 35321 | 0.155 | 1.000 | 0.891 | 0.981 | 0.155 | 0.367 | 0.264 | 0.259 | 0.987 | 36.844 | 17.983 |

| | | | | | | | | | | | | | | | | | | |
|---|---|---|---|---|---|---|---|---|---|---|---|---|---|---|---|---|---|---|
| | | Neural net | 34435 | 93 | 144 | 649 | 35321 | 0.818 | 0.997 | 0.875 | 0.993 | 0.816 | 0.843 | 0.846 | 0.842 | 0.997 | 43.532 | 19.673 |
| | | Neural net (bagging) | 34522 | 6 | 9 | 784 | 35321 | 0.989 | 1.000 | 0.992 | 1.000 | 0.988 | 0.990 | 0.991 | 0.990 | 1.000 | 44.541 | 20.001 |
| Mcl-1 | 5FC4 | Tree | 34065 | 82 | 856 | 139 | 35142 | 0.140 | 0.998 | 0.629 | 0.973 | 0.137 | 0.288 | 0.229 | 0.221 | 0.752 | 22.055 | 8.207 |
| | | Bagged forest | 34074 | 73 | 862 | 133 | 35142 | 0.134 | 0.998 | 0.646 | 0.973 | 0.132 | 0.286 | 0.221 | 0.214 | 0.578 | 16.208 | 3.915 |
| | | Random forest | 34144 | 3 | 822 | 173 | 35142 | 0.174 | 1.000 | 0.983 | 0.977 | 0.174 | 0.408 | 0.295 | 0.289 | 0.930 | 29.583 | 13.227 |
| | | Bayesian | 31061 | 3086 | 493 | 502 | 35142 | 0.505 | 0.910 | 0.140 | 0.898 | 0.414 | 0.227 | 0.219 | 0.183 | 0.811 | 19.420 | 7.619 |
| | | SVM | 34082 | 65 | 712 | 283 | 35142 | 0.284 | 0.998 | 0.813 | 0.978 | 0.283 | 0.473 | 0.421 | 0.413 | 0.835 | 28.476 | 11.538 |
| | | Logistic regression | 34144 | 3 | 989 | 6 | 35142 | 0.006 | 1.000 | 0.667 | 0.972 | 0.006 | 0.062 | 0.012 | 0.011 | 0.817 | 20.023 | 8.704 |
| | | Neural net | 34145 | 2 | 984 | 11 | 35142 | 0.011 | 1.000 | 0.846 | 0.972 | 0.011 | 0.095 | 0.022 | 0.021 | 0.842 | 22.238 | 9.448 |
| | | Neural net (bagging) | 34138 | 9 | 743 | 252 | 35142 | 0.253 | 1.000 | 0.966 | 0.979 | 0.253 | 0.489 | 0.401 | 0.394 | 0.956 | 32.602 | 14.131 |
| | 5MES | Tree | 34048 | 99 | 845 | 150 | 35142 | 0.151 | 0.997 | 0.602 | 0.973 | 0.148 | 0.292 | 0.241 | 0.232 | 0.780 | 21.735 | 9.548 |
| | | Bagged forest | 34051 | 96 | 848 | 147 | 35142 | 0.148 | 0.997 | 0.605 | 0.973 | 0.145 | 0.290 | 0.237 | 0.229 | 0.583 | 17.077 | 4.082 |
| | | Random forest | 34145 | 2 | 881 | 114 | 35142 | 0.115 | 1.000 | 0.983 | 0.975 | 0.115 | 0.331 | 0.205 | 0.200 | 0.921 | 27.269 | 12.463 |
| | | Bayesian | 32781 | 1366 | 605 | 390 | 35142 | 0.392 | 0.960 | 0.222 | 0.944 | 0.352 | 0.268 | 0.284 | 0.257 | 0.819 | 20.125 | 7.840 |
| | | SVM | 34089 | 58 | 628 | 367 | 35142 | 0.369 | 0.998 | 0.864 | 0.980 | 0.367 | 0.557 | 0.517 | 0.509 | 0.877 | 31.897 | 13.428 |
| | | Logistic regression | 34147 | 0 | 993 | 2 | 35142 | 0.002 | 1.000 | 1.000 | 0.972 | 0.002 | 0.044 | 0.004 | 0.004 | 0.817 | 18.213 | 8.583 |
| | | Neural net | 34147 | 0 | 993 | 2 | 35142 | 0.002 | 1.000 | 1.000 | 0.972 | 0.002 | 0.044 | 0.004 | 0.004 | 0.843 | 21.835 | 9.548 |
| | | Neural net (bagging) | 34139 | 8 | 768 | 227 | 35142 | 0.228 | 1.000 | 0.966 | 0.978 | 0.228 | 0.464 | 0.369 | 0.362 | 0.957 | 31.998 | 14.393 |
| Mdm2 | 4ODF | Tree | 2941 | 99 | 229 | 1330 | 4599 | 0.853 | 0.967 | 0.931 | 0.929 | 0.821 | 0.839 | 0.890 | 0.838 | 0.943 | 2.950 | 2.950 |
| | | Bagged forest | 2903 | 137 | 233 | 1326 | 4599 | 0.851 | 0.955 | 0.906 | 0.920 | 0.805 | 0.819 | 0.878 | 0.818 | 0.938 | 2.950 | 2.924 |
| | | Random forest | 2972 | 68 | 266 | 1293 | 4599 | 0.829 | 0.978 | 0.950 | 0.927 | 0.807 | 0.837 | 0.886 | 0.833 | 0.977 | 2.950 | 2.950 |
| | | Bayesian | 2656 | 384 | 198 | 1361 | 4599 | 0.873 | 0.874 | 0.780 | 0.873 | 0.747 | 0.728 | 0.824 | 0.726 | 0.932 | 2.950 | 2.937 |
| | | SVM | 2947 | 93 | 170 | 1389 | 4599 | 0.891 | 0.969 | 0.937 | 0.943 | 0.860 | 0.871 | 0.914 | 0.871 | 0.966 | 2.950 | 2.937 |
| | | Logistic regression | 2931 | 109 | 369 | 1190 | 4599 | 0.763 | 0.964 | 0.916 | 0.896 | 0.727 | 0.765 | 0.833 | 0.758 | 0.942 | 2.884 | 2.924 |
| | | Neural net | 2870 | 170 | 220 | 1339 | 4599 | 0.859 | 0.944 | 0.887 | 0.915 | 0.803 | 0.810 | 0.873 | 0.809 | 0.952 | 2.884 | 2.937 |
| | | Neural net (bagging) | 3029 | 11 | 60 | 1499 | 4599 | 0.962 | 0.996 | 0.993 | 0.985 | 0.958 | 0.966 | 0.977 | 0.965 | 0.999 | 2.949 | 2.950 |
| | 4ZFI | Tree | 2941 | 99 | 255 | 1304 | 4599 | 0.836 | 0.967 | 0.929 | 0.923 | 0.804 | 0.826 | 0.880 | 0.824 | 0.952 | 2.950 | 2.950 |
| | | Bagged forest | 2925 | 115 | 303 | 1256 | 4599 | 0.806 | 0.962 | 0.916 | 0.909 | 0.768 | 0.795 | 0.857 | 0.791 | 0.937 | 2.950 | 2.926 |
| | | Random forest | 2987 | 53 | 297 | 1262 | 4599 | 0.809 | 0.983 | 0.960 | 0.924 | 0.792 | 0.830 | 0.878 | 0.823 | 0.977 | 2.950 | 2.950 |

| | | | | | | | | | | | | | | | | | | |
|---|---|---|---|---|---|---|---|---|---|---|---|---|---|---|---|---|---|---|
| | | Bayesian | 2671 | 369 | 249 | 1310 | 4599 | 0.840 | 0.879 | 0.780 | 0.866 | 0.719 | 0.707 | 0.809 | 0.706 | 0.926 | 2.950 | 2.950 |
| | | SVM | 2925 | 115 | 198 | 1361 | 4599 | 0.873 | 0.962 | 0.922 | 0.932 | 0.835 | 0.847 | 0.897 | 0.846 | 0.962 | 2.950 | 2.950 |
| | | Logistic regression | 2942 | 98 | 327 | 1232 | 4599 | 0.790 | 0.968 | 0.926 | 0.908 | 0.758 | 0.791 | 0.853 | 0.786 | 0.942 | 2.950 | 2.950 |
| | | Neural net | 2881 | 159 | 227 | 1332 | 4599 | 0.854 | 0.948 | 0.893 | 0.916 | 0.802 | 0.811 | 0.873 | 0.811 | 0.956 | 2.950 | 2.950 |
| | | Neural net (bagging) | 3034 | 6 | 56 | 1503 | 4599 | 0.964 | 0.998 | 0.996 | 0.987 | 0.962 | 0.970 | 0.980 | 0.970 | 0.999 | 2.950 | 2.950 |
| Menin | 5DB2 | Tree | 22020 | 0 | 491 | 0 | 22511 | 0.000 | 1.000 | N/A | 0.978 | 0.000 | N/A | N/A | 0.000 | 0.500 | 1.000 | 1.000 |
| | | Bagged forest | 22020 | 0 | 491 | 0 | 22511 | 0.000 | 1.000 | N/A | 0.978 | 0.000 | N/A | N/A | 0.000 | 0.500 | 1.000 | 1.000 |
| | | Random forest | 22020 | 0 | 491 | 0 | 22511 | 0.000 | 1.000 | N/A | 0.978 | 0.000 | N/A | N/A | 0.000 | 0.823 | 14.060 | 6.928 |
| | | Bayesian | 21903 | 117 | 448 | 43 | 22511 | 0.088 | 0.995 | 0.269 | 0.975 | 0.082 | 0.143 | 0.132 | 0.123 | 0.758 | 10.800 | 5.013 |
| | | SVM | 22017 | 3 | 333 | 158 | 22511 | 0.322 | 1.000 | 0.981 | 0.985 | 0.322 | 0.558 | 0.485 | 0.479 | 0.903 | 41.772 | 12.267 |
| | | Logistic regression | 22020 | 0 | 491 | 0 | 22511 | 0.000 | 1.000 | N/A | 0.978 | 0.000 | N/A | N/A | 0.000 | 0.662 | 6.928 | 3.790 |
| | | Neural net | 22020 | 0 | 491 | 0 | 22511 | 0.000 | 1.000 | N/A | 0.978 | 0.000 | N/A | N/A | 0.000 | 0.680 | 4.483 | 3.219 |
| | | Neural net (bagging) | 22020 | 0 | 491 | 0 | 22511 | 0.000 | 1.000 | N/A | 0.978 | 0.000 | N/A | N/A | 0.000 | 0.969 | 34.640 | 15.242 |
| | 6B41 | Tree | 22020 | 0 | 491 | 0 | 22511 | 0.000 | 1.000 | N/A | 0.978 | 0.000 | N/A | N/A | 0.000 | 0.538 | 3.663 | 2.449 |
| | | Bagged forest | 22020 | 0 | 491 | 0 | 22511 | 0.000 | 1.000 | N/A | 0.978 | 0.000 | N/A | N/A | 0.000 | 0.500 | 1.000 | 1.000 |
| | | Random forest | 22020 | 0 | 491 | 0 | 22511 | 0.000 | 1.000 | N/A | 0.978 | 0.000 | N/A | N/A | 0.000 | 0.828 | 11.207 | 5.339 |
| | | Bayesian | 21938 | 82 | 457 | 34 | 22511 | 0.069 | 0.996 | 0.293 | 0.976 | 0.066 | 0.134 | 0.112 | 0.105 | 0.760 | 9.781 | 4.809 |
| | | SVM | 22016 | 4 | 341 | 150 | 22511 | 0.305 | 1.000 | 0.974 | 0.985 | 0.305 | 0.541 | 0.465 | 0.459 | 0.897 | 42.179 | 11.900 |
| | | Logistic regression | 22020 | 0 | 491 | 0 | 22511 | 0.000 | 1.000 | N/A | 0.978 | 0.000 | N/A | N/A | 0.000 | 0.617 | 1.223 | 0.652 |
| | | Neural net | 22020 | 0 | 491 | 0 | 22511 | 0.000 | 1.000 | N/A | 0.978 | 0.000 | N/A | N/A | 0.000 | 0.679 | 3.668 | 2.527 |
| | | Neural net (bagging) | 22020 | 0 | 491 | 0 | 22511 | 0.000 | 1.000 | N/A | 0.978 | 0.000 | N/A | N/A | 0.000 | 0.971 | 37.085 | 16.342 |

**Table S8:** External Statistics for the machine learning models attained on 10 PPI targets and the corresponding protein structures. The best value(s) corresponding to each target protein (across rows) is shown in bold and highlighted in green.

| Target | PDB ID | External statistics | Tree | Bagged forest | Random forest | Bayesian | SVM | Logistic regression | Neural net | Neural net (bagging) |
|---|---|---|---|---|---|---|---|---|---|---|
| BAZ2B | 4XUA | ROC score | 0.534 | 0.5 | 0.537 | 0.53 | 0.521 | 0.532 | 0.53 | **0.54** |
| | | Sensitivity | 0 | 0 | 0 | 0.002 | **0.047** | 0 | 0 | 0 |
| | | Specificity | **1.000** | **1.000** | **1.000** | 0.997 | 0.974 | **1.000** | **1.000** | **1.000** |
| | | Precision | N/A | N/A | N/A | 0.116 | **0.204** | 0 | N/A | N/A |
| | | Concordance | **0.878** | **0.878** | **0.878** | 0.876 | 0.862 | **0.878** | **0.878** | **0.878** |
| | 5.00E+73 | ROC score | 0.524 | 0.5 | 0.546 | 0.525 | 0.54 | 0.52 | 0.538 | **0.553** |
| | | Sensitivity | 0 | 0 | 0 | 0.006 | **0.057** | 0 | 0 | 0 |
| | | Specificity | **1.000** | **1.000** | **1.000** | 0.995 | 0.97 | **1.000** | **1.000** | **1.000** |
| | | Precision | N/A | N/A | N/A | 0.141 | **0.206** | N/A | N/A | N/A |
| | | Concordance | **0.878** | **0.878** | **0.878** | 0.874 | 0.858 | **0.878** | **0.878** | **0.878** |
| Bcl-2 | 2O21 | ROC score | 0.966 | 0.949 | **0.987** | 0.978 | 0.979 | 0.972 | 0.981 | 0.985 |
| | | Sensitivity | 0.868 | 0.893 | 0.894 | **0.906** | 0.896 | 0.661 | 0.851 | 0.896 |
| | | Specificity | **0.999** | **0.998** | **0.999** | 0.985 | **0.999** | **0.999** | **0.999** | **0.999** |
| | | Precision | 0.966 | 0.939 | 0.976 | 0.681 | 0.956 | 0.978 | 0.964 | **0.981** |
| | | Concordance | 0.994 | 0.994 | 0.995 | 0.982 | 0.995 | 0.988 | 0.994 | **0.996** |
| | 4LVT | ROC score | 0.948 | 0.948 | **0.992** | 0.982 | 0.976 | 0.974 | 0.988 | 0.991 |
| | | Sensitivity | 0.861 | 0.879 | 0.878 | 0.895 | **0.898** | 0.612 | 0.872 | **0.898** |
| | | Specificity | 0.999 | 0.998 | 0.999 | 0.986 | 0.998 | **1.000** | 0.999 | 0.999 |
| | | Precision | 0.968 | 0.949 | 0.969 | 0.704 | 0.95 | **0.982** | 0.955 | 0.962 |
| | | Concordance | 0.994 | 0.994 | **0.995** | 0.983 | **0.995** | 0.986 | 0.994 | **0.995** |
| Bcl-xL | 3INQ | ROC score | 0.964 | 0.91 | 0.989 | 0.982 | 0.983 | 0.985 | **0.99** | 0.986 |
| | | Sensitivity | 0.772 | 0.792 | 0.765 | **0.889** | 0.865 | 0.249 | 0.792 | 0.824 |
| | | Specificity | 0.998 | 0.997 | 0.999 | 0.982 | 0.999 | **1.000** | 0.999 | 0.999 |
| | | Precision | 0.896 | 0.854 | 0.94 | 0.491 | 0.923 | 0.923 | 0.935 | **0.944** |
| | | Concordance | 0.994 | 0.993 | 0.995 | 0.98 | **0.996** | 0.985 | 0.995 | **0.996** |
| | 3WIZ | ROC score | 0.979 | 0.916 | 0.989 | 0.975 | 0.981 | 0.986 | 0.988 | **0.993** |
| | | Sensitivity | 0.782 | 0.799 | 0.817 | **0.931** | 0.875 | 0.145 | 0.817 | 0.858 |
| | | Specificity | **1.000** | 0.998 | **1.000** | 0.901 | 0.991 | **1.000** | 0.999 | 0.999 |
| | | Precision | **0.983** | 0.906 | 0.971 | 0.156 | 0.661 | 0.875 | 0.959 | 0.969 |
| | | Concordance | 0.996 | 0.995 | 0.996 | 0.902 | 0.989 | 0.983 | 0.996 | **0.997** |
| BRD4-1 | 5D3L | ROC score | **0.926** | **0.926** | 0.918 | 0.897 | 0.915 | 0.893 | 0.917 | 0.918 |
| | | Sensitivity | 0.826 | 0.83 | 0.818 | 0.834 | **0.846** | 0.779 | 0.842 | **0.846** |
| | | Specificity | 0.881 | **0.891** | 0.861 | 0.823 | 0.854 | 0.874 | 0.854 | 0.861 |
| | | Precision | 0.857 | **0.868** | 0.835 | 0.802 | 0.833 | 0.842 | 0.832 | 0.839 |
| | | Concordance | 0.856 | **0.863** | 0.841 | 0.828 | 0.85 | 0.83 | 0.848 | 0.854 |

| | | | | | | | | | | |
|---|---|---|---|---|---|---|---|---|---|---|
| | 5KU3 | ROC score | 0.901 | 0.901 | 0.911 | 0.886 | 0.908 | 0.887 | 0.906 | **0.927** |
| | | Sensitivity | 0.87 | 0.858 | 0.881 | **0.889** | 0.866 | 0.794 | 0.866 | 0.866 |
| | | Specificity | 0.786 | 0.796 | 0.741 | 0.752 | 0.806 | 0.847 | 0.789 | **0.854** |
| | | Precision | 0.777 | 0.783 | 0.746 | 0.755 | 0.793 | 0.817 | 0.779 | **0.836** |
| | | Concordance | 0.824 | 0.824 | 0.806 | 0.815 | 0.834 | 0.823 | 0.824 | **0.859** |
| CREBBP | 5EIC | ROC score | 0.631 | 0.5 | 0.769 | 0.726 | 0.724 | 0.744 | 0.75 | **0.772** |
| | | Sensitivity | 0 | 0 | 0 | **0.205** | 0.179 | 0 | 0 | 0.003 |
| | | Specificity | **1.000** | **1.000** | **1.000** | 0.958 | 0.98 | **1.000** | **1.000** | **1.000** |
| | | Precision | N/A | N/A | N/A | 0.12 | 0.203 | N/A | N/A | **1.000** |
| | | Concordance | **0.973** | **0.973** | **0.973** | 0.937 | 0.958 | **0.973** | **0.973** | **0.973** |
| | 5MMG | ROC score | 0.689 | 0.5 | 0.691 | 0.712 | 0.732 | 0.721 | 0.746 | **0.788** |
| | | Sensitivity | 0 | 0 | 0 | **0.279** | 0.162 | 0 | 0 | 0.003 |
| | | Specificity | **1.000** | **1.000** | **1.000** | 0.916 | 0.976 | **1.000** | **1.000** | **1.000** |
| | | Precision | N/A | N/A | N/A | 0.086 | 0.157 | N/A | N/A | **1.000** |
| | | Concordance | **0.973** | **0.973** | **0.973** | 0.899 | 0.953 | **0.973** | **0.973** | **0.973** |
| HIV IN | 4CFD | ROC score | 0.822 | 0.827 | 0.801 | 0.74 | 0.805 | 0.742 | 0.784 | **0.832** |
| | | Sensitivity | 0.598 | 0.613 | 0.632 | 0.667 | **0.713** | 0.261 | 0.567 | **0.713** |
| | | Specificity | 0.863 | 0.869 | 0.806 | 0.678 | 0.779 | **0.934** | 0.822 | 0.822 |
| | | Precision | 0.757 | **0.769** | 0.699 | 0.596 | 0.697 | 0.739 | 0.695 | 0.741 |
| | | Concordance | 0.753 | 0.762 | 0.734 | 0.673 | 0.751 | 0.654 | 0.716 | **0.777** |
| | 4CHO | ROC score | 0.792 | 0.807 | 0.791 | 0.73 | 0.816 | 0.714 | 0.753 | **0.824** |
| | | Sensitivity | 0.544 | 0.579 | 0.544 | 0.663 | 0.655 | 0.261 | 0.506 | **0.67** |
| | | Specificity | 0.828 | 0.861 | 0.831 | 0.683 | 0.811 | **0.915** | 0.809 | 0.817 |
| | | Precision | 0.693 | **0.748** | 0.696 | 0.599 | 0.713 | 0.687 | 0.653 | 0.723 |
| | | Concordance | 0.71 | 0.743 | 0.711 | 0.675 | 0.746 | 0.643 | 0.683 | **0.756** |
| XIAP | 1TFT | ROC score | 0.97 | 0.972 | 0.996 | 0.979 | 0.99 | 0.981 | 0.994 | **0.997** |
| | | Sensitivity | 0.644 | 0.674 | 0.626 | **0.962** | 0.782 | 0.162 | 0.762 | 0.838 |
| | | Specificity | 0.997 | 0.995 | 0.999 | 0.866 | 0.997 | **1.000** | 0.998 | 0.999 |
| | | Precision | 0.836 | 0.774 | 0.926 | 0.142 | 0.864 | **0.948** | 0.912 | 0.928 |
| | | Concordance | 0.989 | 0.988 | 0.99 | 0.869 | 0.992 | 0.981 | 0.993 | **0.995** |
| | 5C3H | ROC score | 0.973 | 0.976 | 0.994 | 0.974 | 0.994 | 0.985 | **0.995** | **0.995** |
| | | Sensitivity | 0.709 | 0.718 | 0.571 | **0.862** | 0.788 | 0.141 | 0.776 | **0.862** |
| | | Specificity | 0.995 | 0.994 | 0.999 | 0.952 | 0.996 | **1.000** | 0.997 | 0.997 |
| | | Precision | 0.777 | 0.744 | 0.907 | 0.292 | 0.838 | **0.96** | 0.86 | 0.888 |
| | | Concordance | 0.989 | 0.988 | 0.989 | 0.95 | 0.992 | 0.981 | 0.992 | **0.994** |
| Mcl-1 | 5FC4 | ROC score | 0.742 | 0.586 | 0.854 | 0.783 | 0.776 | 0.813 | 0.843 | **0.859** |
| | | Sensitivity | 0.141 | 0.131 | 0.127 | **0.458** | 0.23 | 0.005 | 0.005 | 0.167 |
| | | Specificity | 0.997 | 0.998 | **1.000** | 0.907 | 0.997 | **1.000** | **1.000** | 0.999 |
| | | Precision | 0.6 | 0.609 | **0.947** | 0.125 | 0.671 | 0.5 | 0.667 | 0.78 |
| | | Concordance | 0.973 | 0.973 | **0.975** | 0.894 | **0.975** | 0.972 | 0.972 | **0.975** |
| | 5MES | ROC score | 0.755 | 0.573 | 0.845 | 0.786 | 0.715 | 0.809 | 0.835 | **0.846** |
| | | Sensitivity | 0.138 | 0.134 | 0.073 | **0.359** | 0.258 | 0 | 0 | 0.148 |
| | | Specificity | 0.997 | 0.997 | **1.000** | 0.957 | 0.994 | **1.000** | **1.000** | 0.999 |
| | | Precision | 0.578 | 0.576 | **0.939** | 0.196 | 0.547 | N/A | 0 | 0.797 |

| | | | | | | | | | | |
|---|---|---|---|---|---|---|---|---|---|---|
| | | Concordance | 0.973 | 0.973 | 0.974 | 0.94 | 0.973 | 0.972 | 0.972 | **0.975** |
| Mdm2 | 4ODF | ROC score | 0.947 | 0.947 | 0.958 | 0.922 | 0.957 | 0.946 | 0.95 | **0.963** |
| | | Sensitivity | 0.84 | 0.849 | 0.801 | 0.867 | **0.891** | 0.765 | 0.861 | 0.879 |
| | | Specificity | 0.968 | 0.951 | **0.975** | 0.853 | 0.952 | 0.97 | 0.943 | 0.961 |
| | | Precision | 0.93 | 0.899 | **0.944** | 0.751 | 0.904 | 0.929 | 0.886 | 0.92 |
| | | Concordance | 0.924 | 0.916 | 0.916 | 0.857 | 0.931 | 0.901 | 0.915 | **0.933** |
| | 4ZFI | ROC score | 0.941 | 0.94 | 0.949 | 0.919 | 0.954 | 0.943 | 0.951 | **0.961** |
| | | Sensitivity | 0.847 | 0.825 | 0.798 | 0.847 | 0.888 | 0.798 | 0.88 | **0.889** |
| | | Specificity | 0.951 | 0.957 | **0.965** | 0.875 | 0.952 | 0.954 | 0.94 | 0.955 |
| | | Precision | 0.898 | 0.908 | **0.922** | 0.776 | 0.904 | 0.899 | 0.883 | 0.91 |
| | | Concordance | 0.916 | 0.912 | 0.909 | 0.866 | 0.93 | 0.901 | 0.92 | **0.933** |
| Menin | 5DB2 | ROC score | 0.5 | 0.5 | **0.674** | 0.622 | 0.614 | 0.62 | 0.646 | 0.646 |
| | | Sensitivity | 0 | 0 | 0 | 0.033 | **0.071** | 0 | 0 | 0 |
| | | Specificity | **1.000** | **1.000** | **1.000** | 0.994 | 0.982 | **1.000** | **1.000** | **1.000** |
| | | Precision | N/A | N/A | N/A | **0.117** | 0.083 | N/A | N/A | N/A |
| | | Concordance | **0.978** | **0.978** | **0.978** | 0.973 | 0.962 | **0.978** | **0.978** | **0.978** |
| | 6B41 | ROC score | 0.536 | 0.5 | **0.669** | 0.601 | 0.608 | 0.633 | 0.656 | 0.637 |
| | | Sensitivity | 0 | 0 | 0 | 0.009 | **0.076** | 0 | 0 | 0 |
| | | Specificity | **1.000** | **1.000** | **1.000** | 0.994 | 0.983 | **1.000** | **1.000** | **1.000** |
| | | Precision | N/A | N/A | N/A | 0.037 | **0.09** | N/A | N/A | N/A |
| | | Concordance | **0.978** | **0.978** | **0.978** | 0.973 | 0.963 | **0.978** | **0.978** | **0.978** |

**Table S9.** Confusion matrices generated when the machine learning models were applied to the test sets of the different PPI targets.

| Target | PDB ID | ML techniques | TN | FP | FN | TP | Total |
|---|---|---|---|---|---|---|---|
| BAZ2B | 4XUA | Tree | 14827 | 0 | 2055 | 0 | 16882 |
| | | Bagged forest | 14827 | 0 | 2055 | 0 | 16882 |
| | | Random forest | 14827 | 0 | 2055 | 0 | 16882 |
| | | Bayesian | 14789 | 38 | 2050 | 5 | 16882 |
| | | SVM | 14448 | 379 | 1958 | 97 | 16882 |
| | | Logistic regression | 14825 | 2 | 2055 | 0 | 16882 |
| | | Neural net | 14827 | 0 | 2055 | 0 | 16882 |
| | | Neural net (bagging) | 14827 | 0 | 2055 | 0 | 16882 |
| | 5E73 | Tree | 14827 | 0 | 2055 | 0 | 16882 |
| | | Bagged forest | 14827 | 0 | 2055 | 0 | 16882 |
| | | Random forest | 14827 | 0 | 2055 | 0 | 16882 |
| | | Bayesian | 14748 | 79 | 2042 | 13 | 16882 |
| | | SVM | 14376 | 451 | 1938 | 117 | 16882 |
| | | Logistic regression | 14827 | 0 | 2055 | 0 | 16882 |
| | | Neural net | 14827 | 0 | 2055 | 0 | 16882 |
| | | Neural net (bagging) | 14827 | 0 | 2055 | 0 | 16882 |
| Bcl-2 | 2O21 | Tree | 14702 | 16 | 70 | 461 | 15249 |

| | | | | | | | |
|---|---|---|---|---|---|---|---|
| | | Bagged forest | 14687 | 31 | 57 | 474 | 15249 |
| | | Random forest | 14704 | 14 | 67 | 564 | 15349 |
| | | Bayesian | 14493 | 225 | 50 | 481 | 15249 |
| | | SVM | 14696 | 22 | 55 | 476 | 15249 |
| | | Logistic regression | 14710 | 8 | 180 | 351 | 15249 |
| | | Neural net | 14701 | 17 | 79 | 452 | 15249 |
| | | Neural net (bagging) | 14709 | 9 | 55 | 476 | 15249 |
| | 4LVT | Tree | 14703 | 15 | 74 | 457 | 15249 |
| | | Bagged forest | 14693 | 25 | 64 | 467 | 15249 |
| | | Random forest | 14703 | 15 | 65 | 466 | 15249 |
| | | Bayesian | 14518 | 200 | 56 | 475 | 15249 |
| | | SVM | 14693 | 25 | 54 | 477 | 15249 |
| | | Logistic regression | 14712 | 6 | 206 | 325 | 15249 |
| | | Neural net | 14696 | 22 | 68 | 463 | 15249 |
| | | Neural net (bagging) | 14699 | 19 | 54 | 477 | 15249 |
| Bcl-xL | 3INQ | Tree | 14725 | 26 | 66 | 223 | 15040 |
| | | Bagged forest | 14712 | 39 | 60 | 229 | 15040 |
| | | Random forest | 14737 | 14 | 68 | 221 | 15040 |
| | | Bayesian | 14485 | 266 | 32 | 257 | 15040 |
| | | SVM | 14730 | 21 | 39 | 250 | 15040 |
| | | Logistic regression | 14745 | 6 | 217 | 72 | 15040 |
| | | Neural net | 14735 | 16 | 60 | 229 | 15040 |
| | | Neural net (bagging) | 14737 | 14 | 51 | 238 | 15040 |
| | 3WIZ | Tree | 14747 | 4 | 63 | 226 | 15040 |
| | | Bagged forest | 14727 | 24 | 58 | 231 | 15040 |
| | | Random forest | 14744 | 7 | 53 | 236 | 15040 |
| | | Bayesian | 13295 | 1456 | 20 | 269 | 15040 |
| | | SVM | 14621 | 130 | 36 | 253 | 15040 |
| | | Logistic regression | 14745 | 6 | 247 | 42 | 15040 |
| | | Neural net | 14741 | 10 | 53 | 236 | 15040 |
| | | Neural net (bagging) | 14743 | 8 | 41 | 248 | 15040 |
| BRD4-1 | 5D3L | Tree | 259 | 35 | 44 | 209 | 547 |
| | | Bagged forest | 262 | 32 | 43 | 210 | 547 |
| | | Random forest | 253 | 41 | 46 | 207 | 547 |
| | | Bayesian | 242 | 52 | 42 | 211 | 547 |
| | | SVM | 251 | 43 | 39 | 214 | 547 |
| | | Logistic regression | 257 | 37 | 56 | 197 | 547 |
| | | Neural net | 251 | 43 | 40 | 213 | 547 |
| | | Neural net (bagging) | 253 | 41 | 39 | 214 | 547 |
| | 5KU3 | Tree | 231 | 63 | 33 | 220 | 547 |
| | | Bagged forest | 234 | 60 | 36 | 217 | 547 |
| | | Random forest | 218 | 76 | 30 | 223 | 547 |
| | | Bayesian | 221 | 73 | 28 | 225 | 547 |

| | | | | | | | |
|---|---|---|---|---|---|---|---|
| | | SVM | 237 | 57 | 34 | 219 | 547 |
| | | Logistic regression | 249 | 45 | 52 | 201 | 547 |
| | | Neural net | 232 | 62 | 34 | 219 | 547 |
| | | Neural net (bagging) | 251 | 43 | 34 | 219 | 547 |
| CREBBP | 5EIC | Tree | 13896 | 0 | 390 | 0 | 14286 |
| | | Bagged forest | 13896 | 0 | 390 | 0 | 14286 |
| | | Random forest | 13896 | 0 | 390 | 0 | 14286 |
| | | Bayesian | 13311 | 585 | 310 | 80 | 14286 |
| | | SVM | 13621 | 275 | 320 | 70 | 14286 |
| | | Logistic regression | 13896 | 0 | 390 | 0 | 14286 |
| | | Neural net | 13896 | 0 | 390 | 0 | 14286 |
| | | Neural net (bagging) | 13896 | 0 | 389 | 1 | 14286 |
| | 5MMG | Tree | 13897 | 0 | 390 | 0 | 14287 |
| | | Bagged forest | 13897 | 0 | 390 | 0 | 14287 |
| | | Random forest | 13896 | 0 | 390 | 0 | 14286 |
| | | Bayesian | 12736 | 1161 | 281 | 109 | 14287 |
| | | SVM | 13559 | 338 | 327 | 63 | 14287 |
| | | Logistic regression | 13897 | 0 | 390 | 0 | 14287 |
| | | Neural net | 13897 | 0 | 390 | 0 | 14287 |
| | | Neural net (bagging) | 13897 | 0 | 389 | 1 | 14287 |
| HIV IN | 4CFD | Tree | 316 | 50 | 105 | 156 | 627 |
| | | Bagged forest | 318 | 48 | 101 | 160 | 627 |
| | | Random forest | 295 | 71 | 96 | 165 | 627 |
| | | Bayesian | 248 | 118 | 87 | 174 | 627 |
| | | SVM | 285 | 81 | 75 | 186 | 627 |
| | | Logistic regression | 342 | 24 | 193 | 68 | 627 |
| | | Neural net | 301 | 65 | 113 | 148 | 627 |
| | | Neural net (bagging) | 301 | 65 | 75 | 186 | 627 |
| | 4CHO | Tree | 303 | 63 | 119 | 142 | 627 |
| | | Bagged forest | 315 | 51 | 110 | 151 | 627 |
| | | Random forest | 304 | 62 | 119 | 142 | 627 |
| | | Bayesian | 250 | 116 | 88 | 173 | 627 |
| | | SVM | 297 | 69 | 90 | 171 | 627 |
| | | Logistic regression | 335 | 31 | 193 | 68 | 627 |
| | | Neural net | 296 | 70 | 129 | 132 | 627 |
| | | Neural net (bagging) | 299 | 67 | 86 | 175 | 627 |
| XIAP | 1TFT | Tree | 14755 | 43 | 121 | 219 | 15138 |
| | | Bagged forest | 14731 | 67 | 111 | 229 | 15138 |
| | | Random forest | 14781 | 17 | 127 | 213 | 15138 |
| | | Bayesian | 12821 | 1977 | 13 | 327 | 15138 |
| | | SVM | 14756 | 42 | 74 | 266 | 15138 |
| | | Logistic regression | 14795 | 3 | 285 | 55 | 15138 |
| | | Neural net | 14773 | 25 | 81 | 259 | 15138 |

| | | | | | | | |
|---|---|---|---|---|---|---|---|
| | | Neural net (bagging) | 14776 | 22 | 55 | 285 | 15138 |
| | 5C3H | Tree | 14729 | 69 | 99 | 241 | 15138 |
| | | Bagged forest | 14714 | 84 | 96 | 244 | 15138 |
| | | Random forest | 14778 | 20 | 146 | 194 | 15138 |
| | | Bayesian | 14088 | 710 | 47 | 293 | 15138 |
| | | SVM | 14746 | 52 | 72 | 268 | 15138 |
| | | Logistic regression | 14796 | 2 | 292 | 48 | 15138 |
| | | Neural net | 14755 | 43 | 76 | 264 | 15138 |
| | | Neural net (bagging) | 14761 | 37 | 47 | 293 | 15138 |
| Mcl-1 | 5FC4 | Tree | 14595 | 40 | 366 | 60 | 15061 |
| | | Bagged forest | 14599 | 36 | 370 | 56 | 15061 |
| | | Random forest | 14632 | 3 | 372 | 54 | 15061 |
| | | Bayesian | 13273 | 1362 | 231 | 195 | 15061 |
| | | SVM | 14587 | 48 | 328 | 98 | 15061 |
| | | Logistic regression | 14633 | 2 | 424 | 2 | 15061 |
| | | Neural net | 14634 | 1 | 424 | 2 | 15061 |
| | | Neural net (bagging) | 14615 | 20 | 355 | 71 | 15061 |
| | 5MES | Tree | 14592 | 43 | 367 | 59 | 15061 |
| | | Bagged forest | 14593 | 42 | 369 | 57 | 15061 |
| | | Random forest | 14633 | 2 | 395 | 31 | 15061 |
| | | Bayesian | 14009 | 626 | 273 | 153 | 15061 |
| | | SVM | 14544 | 91 | 316 | 110 | 15061 |
| | | Logistic regression | 14635 | 0 | 426 | 0 | 15061 |
| | | Neural net | 14634 | 1 | 426 | 0 | 15061 |
| | | Neural net (bagging) | 14619 | 16 | 363 | 63 | 15061 |
| Mdm2 | 4ODF | Tree | 1261 | 42 | 107 | 561 | 1971 |
| | | Bagged forest | 1239 | 64 | 101 | 567 | 1971 |
| | | Random forest | 1271 | 32 | 133 | 535 | 1971 |
| | | Bayesian | 1111 | 192 | 89 | 579 | 1971 |
| | | SVM | 1240 | 63 | 73 | 595 | 1971 |
| | | Logistic regression | 1264 | 39 | 157 | 511 | 1971 |
| | | Neural net | 1229 | 74 | 93 | 575 | 1971 |
| | | Neural net (bagging) | 1252 | 51 | 81 | 587 | 1971 |
| | 4ZFI | Tree | 1239 | 64 | 102 | 566 | 1971 |
| | | Bagged forest | 1247 | 56 | 117 | 551 | 1971 |
| | | Random forest | 1258 | 45 | 135 | 533 | 1971 |
| | | Bayesian | 1140 | 163 | 102 | 566 | 1971 |
| | | SVM | 1240 | 63 | 75 | 593 | 1971 |
| | | Logistic regression | 1243 | 60 | 135 | 533 | 1971 |
| | | Neural net | 1225 | 78 | 80 | 588 | 1971 |
| | | Neural net (bagging) | 1244 | 59 | 74 | 594 | 1971 |
| Menin | 5DB2 | Tree | 9437 | 0 | 211 | 0 | 9648 |

| | | | | | | | |
|---|---|---|---|---|---|---|---|
| | | Bagged forest | 9437 | 0 | 211 | 0 | 9648 |
| | | Random forest | 9437 | 0 | 211 | 0 | 9648 |
| | | Bayesian | 9384 | 53 | 204 | 7 | 9648 |
| | | SVM | 9271 | 166 | 196 | 15 | 9648 |
| | | Logistic regression | 9437 | 0 | 211 | 0 | 9648 |
| | | Neural net | 9437 | 0 | 211 | 0 | 9648 |
| | | Neural net (bagging) | 9437 | 0 | 211 | 0 | 9648 |
| | 6B41 | Tree | 9437 | 0 | 211 | 0 | 9648 |
| | | Bagged forest | 9437 | 0 | 211 | 0 | 9648 |
| | | Random forest | 9437 | 0 | 211 | 0 | 9648 |
| | | Bayesian | 9385 | 52 | 209 | 2 | 9648 |
| | | SVM | 9276 | 161 | 195 | 16 | 9648 |
| | | Logistic regression | 9437 | 0 | 211 | 0 | 9648 |
| | | Neural net | 9437 | 0 | 211 | 0 | 9648 |
| | | Neural net (bagging) | 9437 | 0 | 211 | 0 | 9648 |

**Table S10.** External enrichment factors (EF1% and EF5%) for the machine learning models for the different PPI targets. The best enrichment score(s) corresponding to each target protein (across rows) is shown in bold and highlighted in green.

| Target | PDB ID | Enrichment factors | Tree | Bagged forest | Random forest | Bayesian | SVM | Logistic regression | Neural net | Neural net (bagging) |
|---|---|---|---|---|---|---|---|---|---|---|
| BAZ2B | 4XUA | EF 1% | 1.706 | 1.000 | 1.565 | 1.222 | 1.907 | 1.271 | 1.467 | **2.005** |
| | | EF 5% | **1.526** | 1.000 | 1.431 | 1.265 | 1.518 | 1.236 | 1.256 | 1.479 |
| | 5E73 | EF 1% | 1.530 | 1.000 | 1.956 | 1.320 | **2.200** | 0.880 | 1.467 | 1.858 |
| | | EF 5% | 1.397 | 1.000 | 1.450 | 1.139 | **1.489** | 1.119 | 1.363 | **1.489** |
| Bcl-2 | 2O21 | EF 1% | 28.459 | 27.752 | **28.718** | 28.602 | **28.718** | 27.584 | **28.718** | **28.718** |
| | | EF 5% | 18.399 | 18.048 | **19.107** | 18.241 | 18.730 | 18.278 | 18.806 | 18.844 |
| | 4LVT | EF 1% | 28.583 | 27.736 | **28.718** | **28.718** | **28.718** | 28.151 | **28.718** | **28.718** |
| | | EF 5% | 17.970 | 17.974 | 18.806 | 18.090 | 18.730 | 18.165 | 18.693 | **18.957** |
| Bcl-xL | 3INQ | EF 1% | 49.757 | 45.272 | 51.695 | 49.960 | 51.348 | 48.225 | 51.695 | **52.042** |
| | | EF 5% | 18.033 | 16.581 | **18.893** | 18.408 | **18.893** | 18.547 | 18.824 | 18.824 |
| | 3WIZ | EF 1% | 51.794 | 47.425 | **52.042** | 49.960 | 50.654 | 48.572 | **52.042** | **52.042** |
| | | EF 5% | 18.738 | 16.784 | 19.239 | 17.509 | 18.478 | 18.270 | 19.170 | **19.308** |
| BRD4-1 | 5D3L | EF 1% | **2.162** | **2.162** | **2.162** | 1.730 | **2.162** | **2.162** | **2.162** | **2.162** |
| | | EF 5% | 2.137 | 2.115 | 2.082 | 2.082 | **2.162** | 2.002 | **2.162** | **2.162** |
| | 5KU3 | EF 1% | **2.162** | **2.162** | **2.162** | **2.162** | **2.162** | **2.162** | **2.162** | **2.162** |
| | | EF 5% | 2.082 | **2.162** | **2.162** | 2.002 | 2.082 | 2.082 | **2.162** | **2.162** |
| CREBBP | 5EIC | EF 1% | 2.992 | 1.000 | 12.124 | 8.771 | 9.803 | 4.901 | 6.965 | **12.382** |
| | | EF 5% | 2.454 | 1.000 | 5.695 | 4.258 | 4.874 | 4.053 | 4.412 | **5.900** |
| | 5MMG | EF 1% | 4.161 | 1.000 | 1.548 | 4.128 | 8.513 | 5.160 | 5.676 | **9.029** |

| | | | | | | | | | | |
|---|---|---|---|---|---|---|---|---|---|---|
| | | EF 5% | 2.659 | 1.000 | 2.155 | 2.976 | 4.720 | 4.002 | 3.540 | **6.054** |
| HIV IN | 4CFD | EF 1% | **2.402** | **2.402** | **2.402** | **2.402** | **2.402** | **2.402** | **2.402** | **2.402** |
| | | EF 5% | **2.325** | 2.247 | 2.247 | 2.092 | 2.092 | 2.247 | 2.247 | **2.325** |
| | 4CHO | EF 1% | 2.002 | **2.402** | **2.402** | 1.602 | 1.602 | 2.002 | **2.402** | **2.402** |
| | | EF 5% | 2.170 | 2.092 | **2.247** | 2.015 | 2.015 | 1.627 | 2.015 | **2.247** |
| XIAP | 1TFT | EF 1% | 38.037 | 38.184 | **43.934** | 34.498 | 42.165 | 37.152 | 42.165 | 43.344 |
| | | EF 5% | 17.374 | 17.079 | 19.435 | 17.727 | 18.846 | 17.845 | 19.082 | **19.553** |
| | 5C3H | EF 1% | 40.690 | 38.332 | 43.049 | 33.909 | 42.754 | 36.857 | 43.344 | **43.934** |
| | | EF 5% | 17.939 | 17.842 | 19.082 | 15.901 | 19.140 | 18.080 | 19.317 | **19.435** |
| Mcl-1 | 5FC4 | EF 1% | 19.798 | 17.694 | 23.570 | 18.856 | **23.805** | 17.913 | 17.206 | 23.098 |
| | | EF 5% | 7.175 | 4.191 | 9.954 | 7.043 | 9.156 | 8.451 | 9.296 | **10.329** |
| | 5MES | EF 1% | 18.856 | 15.130 | 22.627 | 16.734 | 22.155 | 18.149 | 21.920 | **23.570** |
| | | EF 5% | 9.045 | 3.701 | **9.531** | 7.137 | 8.639 | 8.123 | 9.296 | 9.437 |
| Mdm2 | 4ODF | EF 1% | **2.951** | **2.951** | **2.951** | 2.795 | **2.951** | 2.795 | **2.951** | **2.951** |
| | | EF 5% | **2.951** | **2.951** | **2.951** | 2.860 | 2.920 | 2.920 | 2.920 | **2.951** |
| | 4ZFI | EF 1% | 2.816 | 2.795 | **2.951** | 2.640 | **2.951** | **2.951** | **2.951** | 2.795 |
| | | EF 5% | 2.899 | 2.885 | **2.920** | 2.800 | 2.890 | 2.890 | 2.890 | 2.890 |
| Menin | 5DB2 | EF 1% | 1.000 | 1.000 | 6.192 | 3.810 | **6.668** | 5.716 | 3.810 | 5.716 |
| | | EF 5% | 1.000 | 1.000 | 3.036 | 2.751 | 2.277 | 2.941 | 2.846 | **3.131** |
| | 6B41 | EF 1% | 3.045 | 1.000 | 3.334 | 1.905 | **6.668** | 1.905 | 2.382 | 3.334 |
| | | EF 5% | 2.403 | 1.000 | 2.751 | 2.751 | **3.036** | 1.138 | 2.467 | 2.561 |
